# Routine metabolic–adiposity indices and incident cardiometabolic multimorbidity among adults with CKM stages 0–3: a prospective cohort study

**DOI:** 10.1186/s12872-026-06385-5

**Published:** 2026-08-08

**Authors:** Zhenyu Geng, Yawei Rong, Jiahao Liu, Haochen Yang, Yulun Wang, Xunlu Yin, Xiangning He, Qian Chen, Fangfang Zhao, Guojun Wang

**Affiliations:** 1 Department of Brain Injury Laboratory, The Affiliated Taian City Central Hospital of Qingdao University, Taian, Shandong Province China; 2 Department of Neuroimmunology and Infectious Diseases, The Affiliated Taian City Central Hospital of Qingdao University, Taian, China; 3 Department of Neurosurgery, The Affiliated Taian City Central Hospital of Qingdao University, Taian, China; 4 Cerebrovascular Disease Screening and Follow-up Office, The Affiliated Taian City Central Hospital of Qingdao University, Taian, China

**Keywords:** CHG, TyG, Cardiovascular-kidney-metabolic syndrome, Cardiometabolic multimorbidity, CHARLS, Metabolic–adiposity phenotype, Cox regression

## Abstract

**Background:**

Cardiometabolic multimorbidity (CMM) is an increasing burden in ageing populations. Cardiovascular-kidney-metabolic (CKM) stages 0–3 provide an upstream framework for identifying metabolic, renal, and cardiovascular vulnerability before overt multimorbidity. We evaluated whether routinely derived metabolic–adiposity indices add useful risk-phenotype information beyond established clinical factors.

**Methods:**

This prospective CHARLS cohort included 7,062 adults aged ≥ 45 years who were free of CMM and had CKM stages 0–3 at baseline. Twenty-nine cholesterol–high-density lipoprotein cholesterol–glucose (CHG)-derived, triglyceride–glucose (TyG)-derived, and adiposity/body-shape indices were evaluated; the main text focused on RFM, CHG-RFM, TyG-RFM, BMI, and WC. Model 3 was used to anchor the primary association interpretation, while Model 4 was retained as an extended clinical-adjustment model to assess robustness after broader adjustment. Benjamini–Hochberg correction, inter-index correlations, paired CHG–TyG models, proportional-hazards diagnostics, prediction analyses, and sensitivity analyses were performed.

**Results:**

Among 7,062 participants, 613 developed incident CMM (8.7%). In Model 3, the per-SD HRs (95% CIs) were 1.85 (1.52–2.25) for RFM, 1.72 (1.47–2.03) for CHG-RFM, 1.67 (1.41–1.98) for TyG-RFM, 1.30 (1.20–1.41) for BMI, and 1.34 (1.23–1.46) for WC. The corresponding estimates remained broadly consistent in Model 4. After Benjamini–Hochberg correction, 28 of 29 Model 3 per-SD associations remained supported; ABSI was the exception. Correlations were substantial among structurally related indices, and paired CHG–TyG models showed marked mutual attenuation. The clinical baseline AUC was 0.787, whereas selected index-extended models achieved AUCs of approximately 0.799–0.800, indicating modest absolute improvement.

**Conclusions:**

Selected routine metabolic–adiposity indices were associated with incident CMM among adults with CKM stages 0–3. RFM-related indices showed stronger association signals, whereas BMI and WC remain more accessible measures. The broadly consistent Model 3 and Model 4 estimates supported the overall association pattern. However, because inter-index overlap was substantial and incremental predictive gains were modest, these indices should be viewed as candidate adjunctive risk markers rather than independent biomarkers or clinically validated decision tools.

**Supplementary Information:**

The online version contains supplementary material available at https://doi.org/10.1186/s12872-026-06385-5.

## Research insights

**Table:** 

Question	Response
What is currently known about this topic?	CMM is an increasing burden in ageing populations. CKM stages 0–3 represent an upstream risk window, while routinely derived metabolic–adiposity indices summarize overlapping rather than independent risk domains.
What is the key research question?	Can representative routine metabolic–adiposity indices identify adults with CKM stages 0–3 who are at higher risk of incident CMM, and how much information do they add beyond clinical factors?
What is new?	The study integrates CHG-derived, TyG-derived, and adiposity/body-shape constructs, quantifies their overlap, and distinguishes association strength from modest incremental prediction.
How might this study influence clinical practice?	These indices may support preliminary risk recognition as candidate adjunctive markers, but they should not replace established clinical assessment and require external validation.

## Introduction

With population ageing, multimorbidity has become one of the most prominent clinical and public health challenges among middle-aged and older adults. CMM, commonly defined as the coexistence of at least two cardiometabolic diseases such as diabetes, heart disease, and stroke, is associated with functional decline, reduced quality of life, impaired cognitive and physical health, increased healthcare resource use, and higher mortality risk [1–3]. Identifying individuals at elevated risk before the development of CMM is therefore important for prevention in ageing populations.

Cardiovascular-kidney-metabolic (CKM) syndrome provides a clinically meaningful framework for evaluating the development of CMM [4, 5]. CKM emphasizes the interplay among excess or dysfunctional adiposity, insulin resistance, glucose and lipid metabolic disorders, renal dysfunction, and cardiovascular injury [4, 5]. CKM stages 0–3 cover the period from no apparent CKM risk factors to adiposity abnormalities, metabolic or renal abnormalities, and subclinical high cardiovascular risk. These stages represent a window before overt clinical cardiovascular disease (CVD) in which risk identification and risk-factor management may be particularly relevant.

Routine metabolic and adiposity indices derived from anthropometric and laboratory measures may characterize complementary aspects of cardiometabolic risk. TyG-related indices reflect glucose–triglyceride metabolism, whereas CHG-related indices combine cholesterol, HDL-C, and glucose information. BMI, WC, WHtR, CVAI, BRI, ABSI, WWI, and RFM describe general, central, or body-shape-related adiposity [6–16]. Composite CHG- and TyG-derived indices may therefore integrate metabolic and adiposity information, but substantial structural overlap is expected.

Previous CHARLS studies have linked TyG-, CHG-, lipid-, and adiposity-related indices to CMM, cardiovascular outcomes, and CKM progression [17–31]. However, most evaluated individual index families and focused primarily on associations. We therefore compared CHG-derived, TyG-derived, and adiposity/body-shape indices within a common CKM stages 0–3 cohort, focusing on five representative indices and evaluating inter-index overlap and incremental information beyond clinical factors.

Accordingly, using the nationally representative longitudinal CHARLS cohort [32], we examined routine metabolic–adiposity risk phenotypes among adults with CKM stages 0–3 who were free of CMM at baseline. The primary objective was to estimate Model 3 associations for five representative indices—RFM, CHG-RFM, TyG-RFM, BMI, and WC—while retaining complete results for all 29 indices in the Supplementary Materials. Model 4 was retained as an extended clinical-adjustment analysis to assess whether the principal association pattern remained evident after broader adjustment for CKM stage and routine clinical indicators. Secondary analyses evaluated inter-index overlap, modest incremental prediction, CKM stage-stratified associations, exploratory mediation, and robustness.

## Methods

### Study design, data source, and study participants

This study was a nationally representative prospective cohort analysis based on the China Health and Retirement Longitudinal Study (CHARLS). The CHARLS baseline survey was initiated in 2011, followed by subsequent follow-ups in 2013, 2015, 2018, and 2020 [32].

Starting from the CHARLS 2011 baseline sample, participants were excluded based on age, baseline CMM status, availability of key exposure components, baseline CKM stage, follow-up information, and required covariates. The final analytic cohort comprised 7,062 adults aged ≥ 45 years who were free of CMM and had CKM stages 0–3 at baseline. Baseline characteristics of participants included in the final analytic cohort and those not included were compared using standardized mean differences (Supplementary Table S2A).

CKM staging in this study was based on the 2023 American Heart Association Presidential Advisory and Scientific Statement on CKM syndrome [4, 5], and was constructed according to the strategy used in published CHARLS studies of TyG-derived indices in CKM stages 0–3 [21]. The detailed operational definitions of each stage are provided in Supplementary Table S1.

Briefly, CKM stage 0 indicates the absence of CKM risk factors; stage 1 indicates excess or dysfunctional adiposity; stage 2 indicates metabolic risk factors and/or chronic kidney disease; stage 3 indicates subclinical high cardiovascular risk; and stage 4 indicates established clinical cardiovascular disease. Because stage 4 already includes overt clinical cardiovascular events, and because the outcome CMM in this study consisted of at least two of diabetes, heart disease, and stroke, including stage 4 participants in the baseline risk set would result in overlap with the outcome definition and weaken prospective interpretation. Therefore, the primary analysis was restricted to participants who were in CKM stages 0–3 and free of CMM at baseline.

### Outcome definition and construction of event time

The primary outcome was incident cardiometabolic multimorbidity (incident CMM), defined as the coexistence of at least two of diabetes, heart disease, and stroke during follow-up [1, 17, 21]. Participants with prevalent CMM at baseline were excluded from longitudinal analyses.

### Exposure indices

A total of 29 baseline indices were evaluated in three families: 10 CHG-derived indices, 9 adiposity/body-shape indices, and 10 TyG-derived indices. For primary reporting, five indices were selected to represent RFM-related constructs and routinely used adiposity measures: RFM, CHG-RFM, TyG-RFM, BMI, and WC. Results for all 29 indices are provided in the Supplementary Materials.

Key formulas are shown below; complete equations for all 29 indices are provided in Supplementary Table S2C.

$$\:\mathrm{C}\mathrm{H}\mathrm{G}\hspace{0.17em}=\hspace{0.17em}\mathrm{l}\mathrm{n}\:\left[\mathrm{T}\mathrm{C}\:\right(\mathrm{m}\mathrm{m}\mathrm{o}\mathrm{l}/\mathrm{L})\:\times\:\:\mathrm{F}\mathrm{B}\mathrm{G}\:(\mathrm{m}\mathrm{g}/\mathrm{d}\mathrm{L})\:/\:(2\:\times\:\:\mathrm{H}\mathrm{D}\mathrm{L}-\mathrm{C}\:(\mathrm{m}\mathrm{m}\mathrm{o}\mathrm{l}/\mathrm{L})\left)\right]$$

$$\:\mathrm{T}\mathrm{y}\mathrm{G}\hspace{0.17em}=\hspace{0.17em}\mathrm{l}\mathrm{n}\:\left[\mathrm{T}\mathrm{G}\:\right(\mathrm{m}\mathrm{g}/\mathrm{d}\mathrm{L})\:\times\:\:\mathrm{F}\mathrm{B}\mathrm{G}\:(\mathrm{m}\mathrm{g}/\mathrm{d}\mathrm{L})\:/\:2]$$

$$\:\mathrm{B}\mathrm{M}\mathrm{I}\hspace{0.17em}=\hspace{0.17em}\mathrm{w}\mathrm{e}\mathrm{i}\mathrm{g}\mathrm{h}\mathrm{t}\:\left(\mathrm{k}\mathrm{g}\right)\:/\:\left[\mathrm{h}\mathrm{e}\mathrm{i}\mathrm{g}\mathrm{h}\mathrm{t}\:\right(\mathrm{m}\left)\right]^2$$

$$\:\mathrm{R}\mathrm{F}\mathrm{M}\:\left(\mathrm{m}\mathrm{a}\mathrm{l}\mathrm{e}\right)\hspace{0.17em}=\hspace{0.17em}64\hspace{0.17em}-\hspace{0.17em}20\:\times\:\:\left[\mathrm{h}\mathrm{e}\mathrm{i}\mathrm{g}\mathrm{h}\mathrm{t}\:\right(\mathrm{m})\:/\:\mathrm{W}\mathrm{C}\:(\mathrm{m}\left)\right]$$

$$\:\mathrm{R}\mathrm{F}\mathrm{M}\:\left(\mathrm{f}\mathrm{e}\mathrm{m}\mathrm{a}\mathrm{l}\mathrm{e}\right)\hspace{0.17em}=\hspace{0.17em}76\hspace{0.17em}-\hspace{0.17em}20\:\times\:\:\left[\mathrm{h}\mathrm{e}\mathrm{i}\mathrm{g}\mathrm{h}\mathrm{t}\:\right(\mathrm{m})\:/\:\mathrm{W}\mathrm{C}\:(\mathrm{m}\left)\right]$$

Before constructing the composite indices, the original continuous variables used for index calculation and selected clinical covariates were winsorized at the 1st and 99th percentiles according to the analysis script. Indices involving sex-specific formulas, such as CVAI and RFM, were calculated according to the corresponding sex-specific equations. CHG, TyG, and their derived indices were calculated using published formulas and combined with prespecified anthropometric/body-shape measures [6–11, 17–21]. Adiposity/body-shape indices were computed using established equations, with sex-specific formulas applied for CVAI and RFM where appropriate [12–16]. All exposures were modeled both per 1-standard deviation (per-SD) increment and by quartiles (Q1–Q4).

### Covariates and model specification

At baseline, covariate information was collected using standardized questionnaires, physical examinations, and laboratory tests [33]. The covariates included in this study comprised sociodemographic characteristics, lifestyle factors, baseline chronic disease status, CKM stage, and hemodynamic and laboratory indicators. Sociodemographic and lifestyle variables included age, sex, education level, marital status, Hukou, smoking status, and drinking status.

Baseline chronic disease covariates included hypertension, diabetes, dyslipidemia, and kidney disease, all of which were ascertained by integrating self-reported medical history, medication information, and objective measurement results. Hypertension was defined as self-reported hypertension, use of antihypertensive medication, or mean systolic/diastolic blood pressure ≥ 140/90 mmHg [34]. Diabetes was defined as self-reported diabetes, use of glucose-lowering medication, fasting blood glucose ≥ 126 mg/dL, or HbA1c ≥ 6.5%. Dyslipidemia was defined as self-reported dyslipidemia, use of lipid-lowering medication, total cholesterol ≥ 240 mg/dL, triglycerides ≥ 150 mg/dL, LDL-C ≥ 160 mg/dL, or HDL-C < 40 mg/dL. Kidney disease was defined as self-reported kidney disease or eGFR < 60 mL/min/1.73 m² [35]. eGFR was calculated using the Chinese-modified Modification of Diet in Renal Disease (MDRD) equation [36].

Cox regression used a sequential adjustment strategy. Model 1 adjusted for age and sex. Model 2 further adjusted for education level, marital status, Hukou, smoking status, and drinking status. Model 3 additionally adjusted for baseline hypertension, diabetes, dyslipidemia, and kidney disease. Model 3 was used to anchor the primary association interpretation because it controlled for major demographic, lifestyle, and baseline disease-history factors without extensively conditioning on variables that were components or close correlates of the candidate indices.

Model 4 was retained as an extended clinical-adjustment model. In addition to the Model 3 covariates, Model 4 included baseline CKM stage, systolic and diastolic blood pressure, HDL-C, LDL-C, eGFR, uric acid, BUN, hemoglobin, MCV, PCV, platelet count, and white blood cell count. Model 4 was used to assess robustness after broader clinical adjustment. Because several additional covariates overlapped conceptually or mathematically with the index constructs or CKM staging, Model 4 estimates were interpreted as conditional associations under broader adjustment rather than as definitive independent or causal effects.

Each exposure index was entered separately in the Cox models. Quartile analyses used Q1 as the reference group, and P for trend was obtained by entering the ordinal quartile variable into the model.

### Statistical analysis

Baseline characteristics were presented as mean ± standard deviation, median (interquartile range, IQR), or n (%), according to the distribution of each variable. Across different CKM stages, approximately symmetrically distributed continuous variables were compared using Welch’s one-way analysis of variance, whereas skewed continuous variables were compared using the Kruskal–Wallis test. When stratified by whether CMM occurred during follow-up, approximately symmetrically distributed continuous variables were compared using Welch’s t test, whereas skewed continuous variables were compared using the Wilcoxon rank-sum test. Categorical variables were compared using Pearson’s chi-square test or Fisher’s exact test. P values in the baseline table were used only for descriptive comparisons.

Results were expressed as HRs and 95% CIs for each 1-SD increment and for quartiles. The five representative indices were emphasized in the main text, while full results for all 29 indices were reported in the Supplementary Materials. Benjamini–Hochberg false discovery rate (FDR) correction was applied to the 29 per-SD Model 3 P values; q < 0.05 was considered evidence that the association remained supported after multiplicity control. Model 4 results were used to evaluate the consistency of the associations under broader clinical adjustment and were not treated as a separate multiplicity-testing family.

Restricted cubic splines (RCS) were used to evaluate dose–response associations between continuous exposures and incident CMM [37], with knots placed at the 5th, 35th, 65th, and 95th percentiles and the median value of each index used as the reference. Kaplan–Meier curves were also plotted by baseline quartiles of each index to show the cumulative incidence of incident CMM during follow-up, and log-rank tests were used to compare survival distributions across quartile groups. Kaplan–Meier curves and RCS analyses were considered descriptive and exploratory, respectively; primary inference was based on multivariable Cox models.

Long-term predictive performance was evaluated using the time-dependent area under the receiver operating characteristic curve (AUC) at the 8-year prediction horizon. Time-dependent AUCs were estimated using marginal inverse-probability-of-censoring weighting. The clinical baseline model included age, sex, education, marital status, Hukou, smoking, drinking, baseline CKM stage, SBP, DBP, HDL-C, LDL-C, eGFR, uric acid, BUN, hemoglobin, MCV, PCV, platelet count, and white blood cell count. Each candidate index was added separately to the clinical baseline model. Differences between the AUCs of the clinical baseline and index-extended models were evaluated using the iid-representation-based comparison procedure for correlated time-dependent AUCs [38]. IDI, continuous net reclassification improvement (cNRI), survival NRI [39], landmark-based bootstrap internal validation [40], and calibration were evaluated as supplementary measures. Absolute changes in AUC were prioritized in interpretation, and statistical significance in reclassification measures was not equated with clinically decisive improvement.

CKM stage-stratified Cox analyses and multiplicative interaction tests were conducted as secondary analyses to evaluate whether the associations differed across baseline CKM stages. These analyses were reported separately from the sequential adjustment models.

CKM stage progression analysis used the change in CKM stage from 2011 to 2015 as an early-to-middle CKM progression outcome. We evaluated this outcome at three levels: any progression, degree of progression, and direction of progression. Any progression was defined as an increase in CKM stage and was analyzed using binary logistic regression. Degree of progression was classified as no worsening, progression by 1 stage, and progression by ≥ 2 stages. Ordinal logistic regression was used first, and the proportional odds assumption was assessed using the global Brant test; if this assumption was not met, multinomial logistic regression was used. Direction of progression was classified as stage improvement, stage stability, and stage progression, with stable stage used as the reference group for comparison.

Exploratory mediation analysis used a temporal framework of “2011 baseline metabolic/body-shape index → CKM stage progression from 2011 to 2015 → post-2015 incident CMM”. Parametric g-computation and 1,000 bootstrap resamples were used to estimate the total effect, natural direct effect, natural indirect effect, and proportion mediated. This analysis was used to evaluate whether CKM stage progression may partially explain the associations between baseline metabolic/body-shape indices and subsequent CMM. Because this mediation analysis was based on observational data, it relied on assumptions of no unmeasured exposure–mediator, mediator–outcome, and exposure–outcome confounding and was interpreted as exploratory.

Robustness analyses included multiple imputation for missing covariates, exclusion of participants with baseline diabetes, CKM stage-stratified analyses, Fine–Gray competing-risk models [41], and an exploratory CKD-inclusive CMM definition. All statistical analyses were performed using R software version 4.5.2 (R Foundation for Statistical Computing, Vienna, Austria). Tests were two-sided. Nominal *P* < 0.05 was used outside the BH-FDR family, with emphasis on effect estimates, confidence intervals, and consistency rather than isolated significance.

### Missing data, collinearity, and model diagnostics

Primary analyses used the final analytic cohort with the required exposure components, outcome information, and covariates. Multiple imputation by chained equations [42] was used only as a sensitivity analysis for missing baseline covariates under a missing-at-random assumption; derived exposures, CKM stage, follow-up time, and outcomes were not imputed.

To reduce the influence of extreme values on the estimates, after sample selection was completed, the original continuous variables used to construct CHG/TyG and adiposity/body-shape indices, as well as continuous covariates entering the Cox models, were winsorized at the 1st and 99th percentiles. No participants were additionally excluded because of abnormal exposure values or abnormal derived-index values.

Spearman coefficients were calculated among all 29 indices to quantify structural overlap. Selected paired CHG–TyG dual-entry Model 3 analyses (CHG with TyG and CHG-RFM with TyG-RFM) were used to examine mutual attenuation. These models were interpreted as overlap analyses rather than tests of definitive causal independence or superiority. GVIF diagnostics for Model 4 were retained as a secondary collinearity check for the extended clinical-adjustment model.

For the five representative indices, proportional-hazards assumptions in Model 3 were evaluated using Schoenfeld residuals. When the exposure-specific test was *P* < 0.05, a time-varying coefficient model including exposure×log(time) was fitted and HRs were estimated at years 1, 4, and 8. Accordingly, conventional Cox HRs were interpreted as average associations over follow-up rather than effects that were necessarily constant over time.

## Results

### Study population and baseline characteristics

**Fig. 1:**
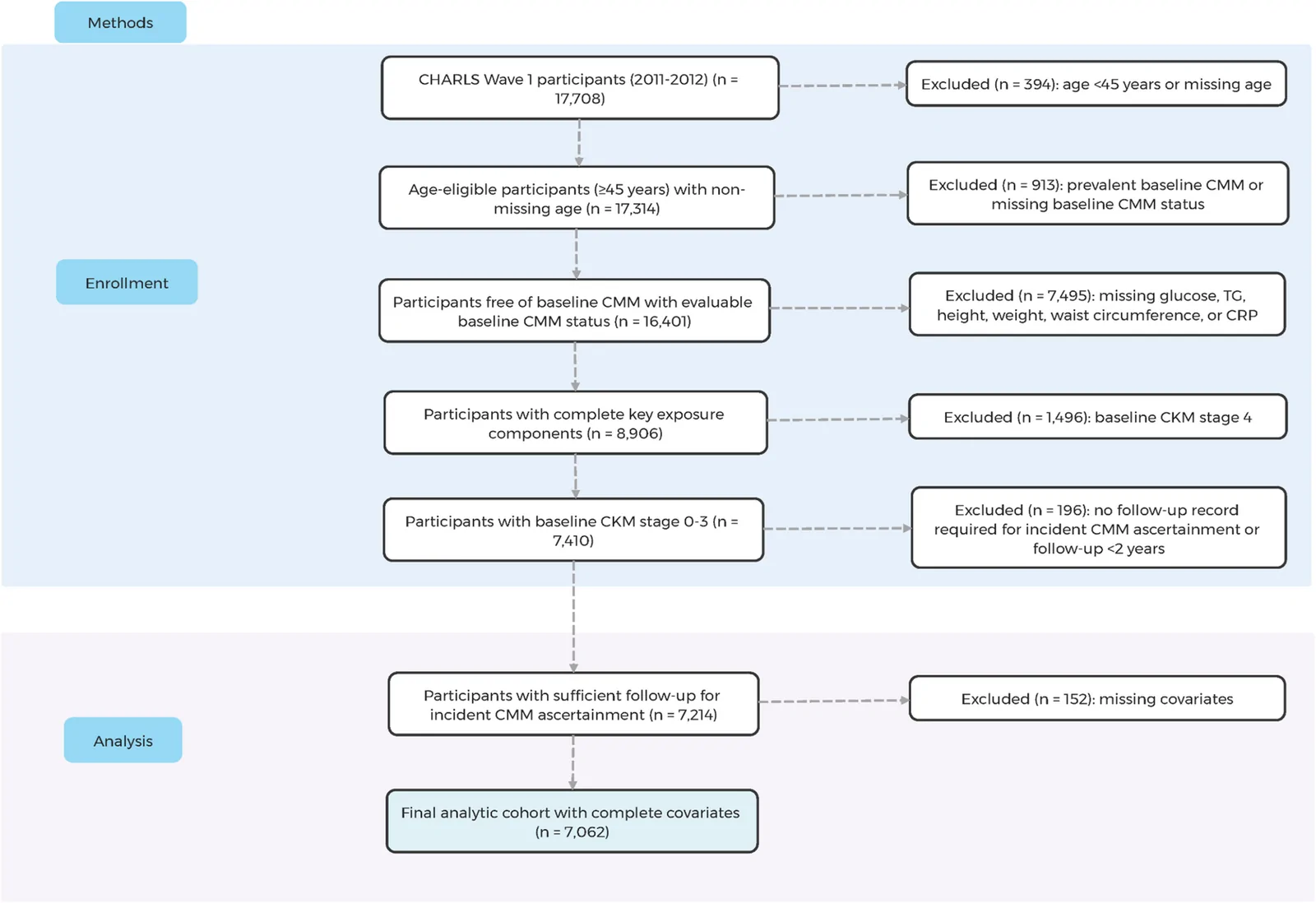
Flowchart of participant selection. The flowchart shows the selection process from the CHARLS 2011 baseline sample to the final analytic cohort. Exclusion criteria included age, baseline CMM status, missing key exposure components, baseline CKM stage, follow-up information, and required covariates. Abbreviations: CHARLS, China Health and Retirement Longitudinal Study; CKM, cardiovascular–kidney–metabolic; CMM, cardiometabolic multimorbidity; FBG, fasting blood glucose; TG, triglycerides; CRP, C-reactive protein

The participant selection process is shown in Fig. 1. The final descriptive cohort included 7,062 participants who were free of CMM and had CKM stages 0–3 at baseline; mean age was approximately 59 years and 48% were men. The median observed follow-up time was 9.0 years (IQR, 9.0–9.0 years), during which 613 participants developed incident CMM (8.7%). Baseline characteristics by CKM stage are shown in Table 1, and characteristics by incident CMM status are shown in Supplementary Table S2B.

Compared with participants not included in the final analytic cohort, included participants differed most in Hukou (SMD 0.35), dyslipidemia (0.31), education (0.20), HDL-C (0.20), triglycerides (robust SMD 0.12), and baseline diabetes (0.11) (Supplementary Table S2A). These differences indicate that availability of exposure and covariate data may have influenced cohort composition and should be considered when generalizing the findings.

**Table 1:** Baseline characteristics by baseline CKM stage

Variable	CKM stage					*P* value^b^
	Overall, *N* = 7062^a^	Stage 0, *N* = 666^a^	Stage 1, *N* = 1,070^a^	Stage 2, *N* = 3,093^a^	Stage 3, *N* = 2,233^a^	
N	7062	666	1070	3093	2233	
Age, years	59 ± 9	56 ± 8	56 ± 8	56 ± 8	65 ± 9	< 0.001
Sex						< 0.001
Male	3359 (48%)	375 (56%)	415 (39%)	827 (27%)	1742 (78%)	
Female	3703 (52%)	291 (44%)	655 (61%)	2266 (73%)	491 (22%)	
Marital status						< 0.001
Married	6270 (89%)	607 (91%)	969 (91%)	2796 (90%)	1898 (85%)	
Others	792 (11%)	59 (8.9%)	101 (9.4%)	297 (9.6%)	335 (15%)	
Education level						0.016
Secondary school or below	6410 (91%)	580 (87%)	973 (91%)	2810 (91%)	2047 (92%)	
High school	578 (8.2%)	79 (12%)	86 (8.0%)	253 (8.2%)	160 (7.2%)	
College or above	74 (1.0%)	7 (1.1%)	11 (1.0%)	30 (1.0%)	26 (1.2%)	
Hukou						< 0.001
Agriculture	6061 (86%)	600 (90%)	940 (88%)	2669 (86%)	1852 (83%)	
Others	1001 (14%)	66 (9.9%)	130 (12%)	424 (14%)	381 (17%)	
Smoking status						< 0.001
No	4252 (60%)	379 (57%)	747 (70%)	2493 (81%)	633 (28%)	
Yes	2810 (40%)	287 (43%)	323 (30%)	600 (19%)	1600 (72%)	
Drinking status						< 0.001
No	4811 (68%)	425 (64%)	756 (71%)	2434 (79%)	1196 (54%)	
Yes	2247 (32%)	241 (36%)	313 (29%)	656 (21%)	1037 (46%)	
Hypertension						< 0.001
No	4475 (63%)	666 (100%)	1070 (100%)	1870 (60%)	869 (39%)	
Yes	2587 (37%)	0 (0%)	0 (0%)	1223 (40%)	1364 (61%)	
Diabetes (%)						< 0.001
No	5966 (84%)	666 (100%)	1070 (100%)	2682 (87%)	1548 (69%)	
Yes	1096 (16%)	0 (0%)	0 (0%)	411 (13%)	685 (31%)	
Dyslipidemia (%)						< 0.001
No	3923 (56%)	666 (100%)	1070 (100%)	1257 (41%)	930 (42%)	
Yes	3139 (44%)	0 (0%)	0 (0%)	1836 (59%)	1303 (58%)	
Kidney disease (%)						< 0.001
No	6553 (93%)	666 (100%)	1070 (100%)	2784 (90%)	2033 (91%)	
Yes	509 (7.2%)	0 (0%)	0 (0%)	309 (10.0%)	200 (9.0%)	
SBP, mmHg	129 ± 21	114 ± 11	116 ± 11	127 ± 19	142 ± 22	< 0.001
DBP, mmHg	75 ± 12	68 ± 9	69 ± 8	75 ± 12	79 ± 12	< 0.001
FBG, mg/dL	102 (94, 113)	92 (87, 96)	102 (95, 108)	103 (95, 113)	107 (97, 128)	< 0.001
HbA1c, %	5.10 (4.90, 5.40)	5.00 (4.80, 5.20)	5.10 (4.80, 5.40)	5.10 (4.90, 5.40)	5.20 (4.90, 5.50)	< 0.001
TC, mg/dL	193 ± 37	176 ± 26	183 ± 26	196 ± 38	200 ± 39	< 0.001
HDL-C, mg/dL	52 ± 15	60 ± 13	60 ± 12	50 ± 15	48 ± 15	< 0.001
LDL-C, mg/dL	116 ± 34	103 ± 24	109 ± 24	118 ± 35	121 ± 36	< 0.001
TG, mg/dL	104 (73, 150)	74 (58, 94)	78 (61, 99)	121 (83, 170)	117 (81, 177)	< 0.001
eGFR, mL/min/1.73 m²	105 (90, 123)	109 (96, 127)	111 (97, 130)	107 (93, 124)	98 (84, 115)	< 0.001
Uric acid, mg/dL	4.43 ± 1.20	4.16 ± 1.06	4.07 ± 1.02	4.24 ± 1.13	4.94 ± 1.27	< 0.001
CRP, mg/L	0.99 (0.53, 2.06)	0.68 (0.39, 1.44)	0.74 (0.46, 1.48)	0.97 (0.53, 1.96)	1.28 (0.68, 2.70)	< 0.001
BUN, mg/dL	15.7 ± 4.3	16.1 ± 4.3	15.6 ± 4.2	15.2 ± 4.2	16.4 ± 4.4	< 0.001
Creatinine, mg/dL	0.78 ± 0.17	0.76 ± 0.16	0.73 ± 0.14	0.74 ± 0.16	0.86 ± 0.18	< 0.001

*CKM* cardiovascular–kidney–metabolic, *FBG* fasting blood glucose, *SBP* systolic blood pressure, *DBP* diastolic blood pressure, *TC* total cholesterol, *HDL-C* high-density lipoprotein cholesterol, *LDL-C* low-density lipoprotein cholesterol, *TG* triglycerides, *eGFR* estimated glomerular filtration rate, *CRP* C-reactive protein, *BUN* blood urea nitrogen, *IQR* interquartile range

^a^ Values are presented as mean ± SD, median (IQR), or n (%), as appropriate

^b^*P* values are descriptive and were calculated using Welch’s one-way ANOVA, Kruskal–Wallis test, Pearson’s chi-square test, or Fisher’s exact test, as appropriate

### Prospective associations of candidate metabolic/body-shape indices with incident CMM: results from sequential Cox models

In Model 3, most candidate indices were positively associated with incident CMM. After Benjamini–Hochberg correction across the 29 per-SD tests, 28 associations remained supported at q < 0.05; ABSI was the exception (Supplementary Table S9). Because many indices shared glucose, lipid, BMI, WC, height, age, or sex components, these findings were interpreted at the level of overlapping metabolic–adiposity constructs rather than as evidence for 28 independent biomarkers.

Among the five representative indices, the Model 3 per-SD HRs (95% CIs) were 1.85 (1.52–2.25) for RFM, 1.72 (1.47–2.03) for CHG-RFM, 1.67 (1.41–1.98) for TyG-RFM, 1.30 (1.20–1.41) for BMI, and 1.34 (1.23–1.46) for WC (Table 2). RFM-related indices therefore showed stronger association signals, while BMI and WC retained smaller but clinically accessible associations.

**Table 2:** Associations of five representative indices with incident CMM in Model 3 and Model 4

Index	Model 3 h per SD (95% CI)	BH q-value	Model 4 h per SD (95% CI)
RFM	1.85 (1.52–2.25)	< 0.001	1.79 (1.46–2.20)
CHG-RFM	1.72 (1.47–2.03)	< 0.001	1.78 (1.47–2.15)
TyG-RFM	1.67 (1.41–1.98)	< 0.001	1.73 (1.44–2.08)
BMI	1.30 (1.20–1.41)	< 0.001	1.30 (1.20–1.42)
WC	1.34 (1.23–1.46)	< 0.001	1.33 (1.21–1.46)

Each index was entered separately. Model 3 adjusted for age, sex, education, marital status, Hukou, smoking, drinking, hypertension, diabetes, dyslipidemia, and kidney disease and was used to anchor the primary association interpretation. Model 4 additionally included baseline CKM stage, SBP, DBP, HDL-C, LDL-C, eGFR, uric acid, BUN, hemoglobin, MCV, PCV, platelet count, and white blood cell count. Model 4 was retained as an extended clinical-adjustment model to assess robustness after broader adjustment; its estimates were not interpreted as definitive independent or causal effects. BH q-values were calculated across the 29 per-SD Model 3 tests

*Abbreviations*: *HR* hazard ratio, *CI* confidence interval, *BH* Benjamini–Hochberg, *CMM* cardiometabolic multimorbidity, *CKM* cardiovascular–kidney–metabolic, *CHG* cholesterol–HDL-C–glucose index, *TyG* triglyceride–glucose index, *RFM* relative fat mass, *BMI* body mass index, *WC* waist circumference

After broader adjustment in Model 4, the corresponding HRs (95% CIs) were 1.79 (1.46–2.20) for RFM, 1.78 (1.47–2.15) for CHG-RFM, 1.73 (1.44–2.08) for TyG-RFM, 1.30 (1.20–1.42) for BMI, and 1.33 (1.21–1.46) for WC. The broadly similar representative-index estimates in Model 3 and Model 4 supported the robustness of the overall association pattern. However, because Model 4 additionally conditioned on CKM stage and several lipid, renal, hemodynamic, and hematologic variables that overlapped with the index constructs, its estimates were not interpreted as definitive independent or causal effects.

**Fig. 2:**
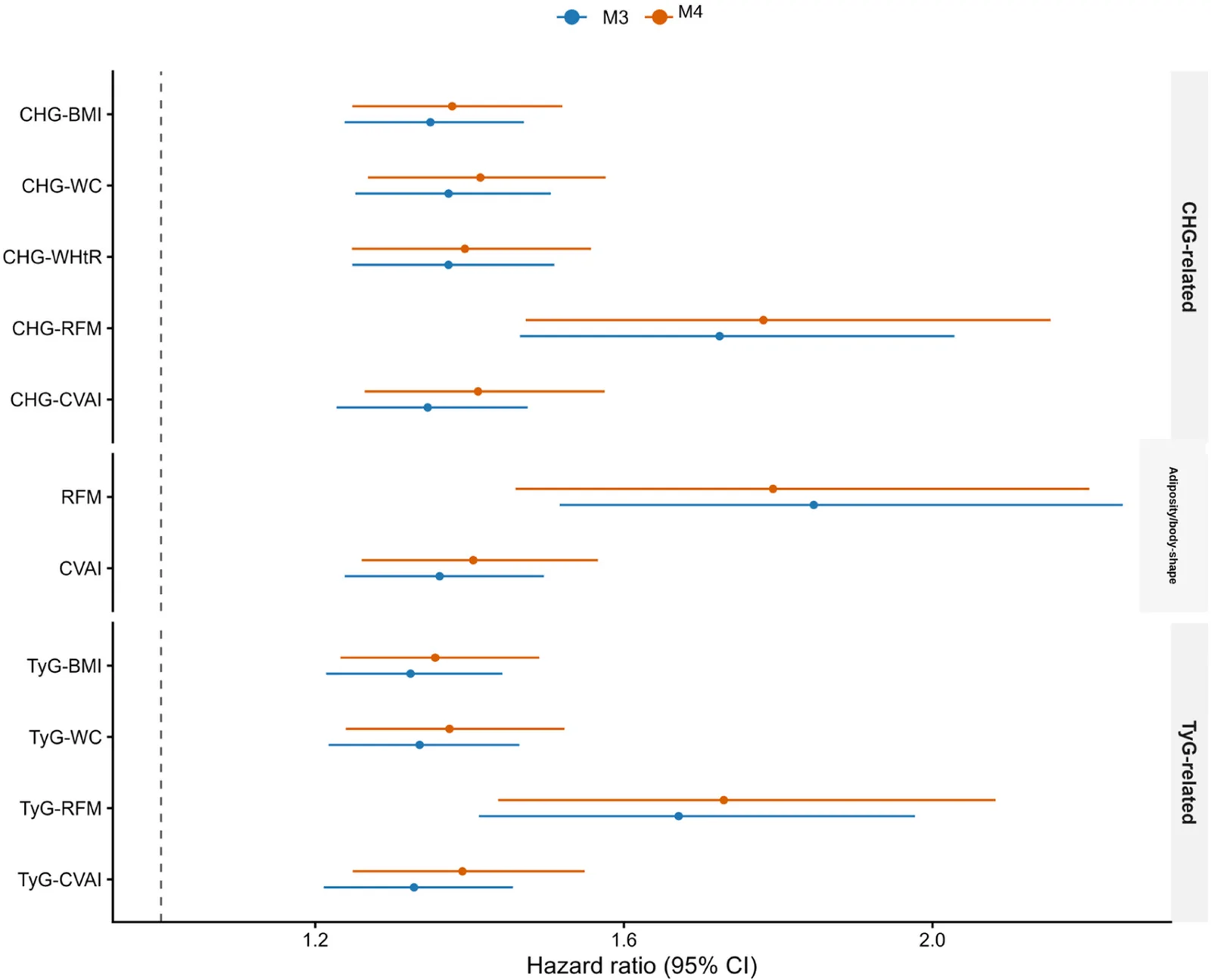
Forest plot for selected indices and incident CMM. The figure shows per-SD HRs and 95% CIs from Model 3 (blue) and Model 4 (orange) for the selected CHG-related, adiposity/body-shape, and TyG-related indices shown. Each index was entered separately, and the vertical dashed line indicates HR = 1. Model 3 adjusted for demographic characteristics, lifestyle factors, and baseline disease history. Model 4 additionally adjusted for CKM stage, blood pressure, lipid, renal, and hematological indicators and served as the extended clinical-adjustment analysis. Abbreviations: HR, hazard ratio; CI, confidence interval; CMM, cardiometabolic multimorbidity; CKM, cardiovascular–kidney–metabolic; CHG, cholesterol–HDL-C–glucose index; TyG, triglyceride–glucose index; RFM, relative fat mass; eGFR, estimated glomerular filtration rate; BUN, blood urea nitrogen

Model 3 and Model 4 estimates are compared in Fig. 2. Full per-SD and quartile results for all 29 indices in Models 1, 2, 3, and 4 are provided in Supplementary Tables S3A and S3B. CKM stage-stratified associations and interaction tests are reported separately in Supplementary Table S3C. Full index-specific quartile, RCS, and Kaplan–Meier results are provided in the Supplementary Materials.

**Fig. 3:**
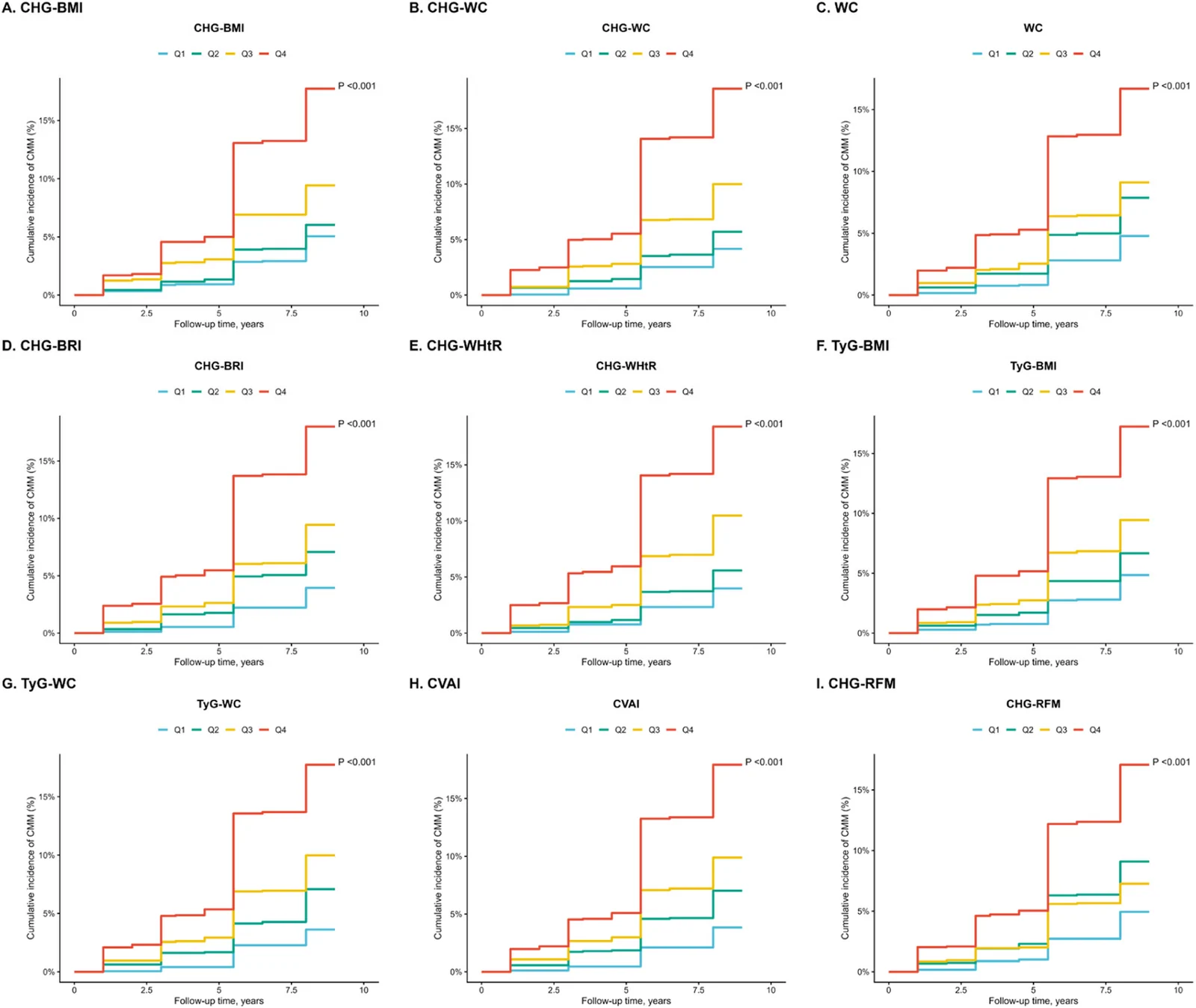
Kaplan–Meier curves for incident CMM. Participants were categorized into quartiles according to the baseline distribution of each index, with Q1 representing the lowest quartile. Curves show the cumulative incidence of incident CMM during follow-up, and between-group differences were assessed using the log-rank test. Panels A–I correspond to CHG-BMI, CHG-WC, WC, CHG-BRI, CHG-WHtR, TyG-BMI, TyG-WC, CVAI, and CHG-RFM, respectively. Abbreviations: CMM, cardiometabolic multimorbidity; CHG, cholesterol–HDL-C–glucose index; TyG, triglyceride–glucose index; BMI, body mass index; WC, waist circumference; BRI, body roundness index; WHtR, waist-to-height ratio; CVAI, Chinese visceral adiposity index; RFM, relative fat mass

Kaplan–Meier curves showed temporal separation in the cumulative incidence of incident CMM across quartile groups of the indices displayed in Fig. 3. The highest quartile group generally accumulated more events than the lowest quartile group, and the corresponding log-rank tests were statistically significant (all *P* < 0.001). These unadjusted curves were interpreted descriptively alongside the adjusted Cox estimates and were not used to establish an index ranking. Kaplan–Meier curves for all 29 candidate indices stratified by index family are shown in Supplementary Figs. S7A–S7C.

### Inter-index overlap and paired CHG–TyG models

Spearman analysis showed substantial overlap among structurally related indices (Supplementary Table S10). CHG correlated with TyG at ρ = 0.747; CHG-RFM correlated with TyG-RFM at ρ = 0.987; RFM correlated with CHG-RFM and TyG-RFM at ρ = 0.968 and 0.970, respectively; and WHtR and BRI were effectively collinear (ρ = 1.000). In paired Model 3 analyses, CHG remained associated after simultaneous entry with TyG (HR 1.22, 95% CI 1.06–1.40), whereas TyG was attenuated (HR 0.97, 95% CI 0.85–1.11). A similar pattern occurred for CHG-RFM and TyG-RFM. Given the very high correlations and shared components, these results indicate overlapping information and mutual attenuation; they do not establish that CHG is superior to TyG or that either index has an independent causal effect (Supplementary Table S11).

### Incremental prediction

**Fig. 4:**
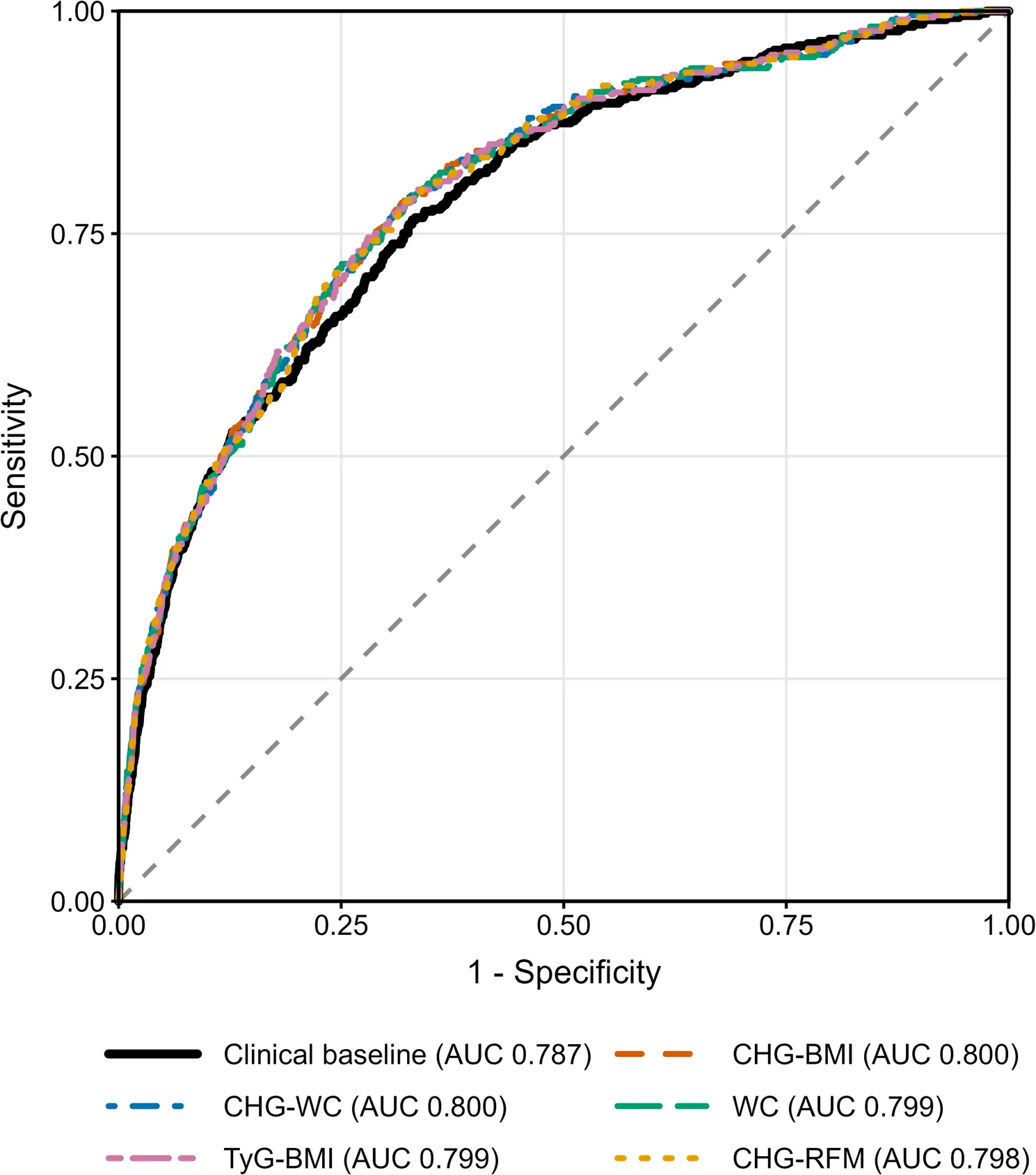
Long-term prediction using index-extended models. The figure shows time-dependent ROC curves for the clinical baseline model and representative index-extended models. Curves are shown for the 8-year prediction horizon. Each index was added separately to the baseline model. To avoid overplotting, only selected representative index-extended models are displayed. Abbreviations: ROC, receiver operating characteristic; AUC, area under the curve; CMM, cardiometabolic multimorbidity; CHG, cholesterol–HDL-C–glucose index; TyG, triglyceride–glucose index; CVAI, Chinese visceral adiposity index; WC, waist circumference; RFM, relative fat mass

The 8-year prediction analysis included 7,013 participants and 568 incident CMM events. The clinical baseline model had an AUC of 0.787; selected index-extended models achieved AUCs of approximately 0.799–0.800, corresponding to absolute increases of approximately 0.011–0.013 (Table 3; Fig. 4).

**Table 3:** Added value of representative indices for CMM prediction

Index	Family	AUC	ΔAUC	*P* for ΔAUC	IDI (95% CI)	cNRI (95% CI)	survival NRI (95% CI)	Corrected C-statistic	Corrected calibration slope
CHG-BMI	CHG-related	0.800	0.0129	< 0.001	0.0111 (0.0042 to 0.0200)	0.1120 (0.0669 to 0.1697)	0.2003 (0.1025 to 0.2909)	0.725	0.921
CHG-WC	CHG-related	0.800	0.0126	< 0.001	0.0113 (0.0048 to 0.0203)	0.1478 (0.0785 to 0.1896)	0.2362 (0.1017 to 0.3238)	0.726	0.920
WC	Adiposity/body-shape	0.799	0.0121	0.002	0.0115 (0.0052 to 0.0214)	0.1413 (0.0729 to 0.1882)	0.2195 (0.1081 to 0.3144)	0.724	0.920
CHG-BRI	CHG-related	0.799	0.0119	< 0.001	0.0088 (0.0033 to 0.0165)	0.1127 (0.0465 to 0.1598)	0.1877 (0.0693 to 0.2711)	0.725	0.920
CHG-WHtR	CHG-related	0.799	0.0119	< 0.001	0.0087 (0.0030 to 0.0163)	0.1159 (0.0469 to 0.1624)	0.1949 (0.0822 to 0.2878)	0.726	0.920
TyG-BMI	TyG-related	0.799	0.0117	< 0.001	0.0110 (0.0041 to 0.0199)	0.1442 (0.0755 to 0.1855)	0.2435 (0.1312 to 0.3191)	0.723	0.918
TyG-WC	TyG-related	0.798	0.0111	0.002	0.0107 (0.0044 to 0.0195)	0.1515 (0.0817 to 0.1996)	0.2588 (0.1328 to 0.3347)	0.725	0.920
CVAI	Adiposity/body-shape	0.798	0.0108	0.006	0.0115 (0.0052 to 0.0206)	0.1289 (0.0623 to 0.1838)	0.2202 (0.1103 to 0.3023)	0.723	0.919
CHG-RFM	CHG-related	0.798	0.0113	0.002	0.0092 (0.0036 to 0.0163)	0.1359 (0.0648 to 0.1784)	0.2410 (0.1177 to 0.3202)	0.726	0.920

Each index was added separately to the clinical baseline model. AUC was estimated using time-dependent ROC analysis at the 8-year prediction horizon with marginal inverse-probability-of-censoring weighting. ΔAUC indicates the difference between the AUC of the index-extended model and that of the clinical baseline model. P values for ΔAUC were obtained using the iid-representation-based comparison of correlated time-dependent AUCs [38]. IDI, cNRI, and survival NRI were used to evaluate incremental predictive performance. Corrected C-statistics and corrected calibration slopes were derived from the 2015 landmark-based bootstrap internal validation. CHG-RFM was retained as a comparator representing the strong-association signal group

*Abbreviations*: *CMM* cardiometabolic multimorbidity, *AUC* area under the curve, *IDI* integrated discrimination improvement, *cNRI* continuous net reclassification improvement, *NRI* net reclassification improvement, *CI* confidence interval, *CHG* cholesterol–HDL-C–glucose index, *TyG* triglyceride–glucose index, *BMI* body mass index, *WC* waist circumference, *BRI* body roundness index, *WHtR* waist-to-height ratio, *CVAI* Chinese visceral adiposity index, *RFM* relative fat mass

Several IDI and NRI estimates were statistically supported, but the absolute discrimination gains were modest. RFM-related indices showed stronger association estimates but did not produce the largest AUC increments, indicating that association strength and incremental prediction capture different properties.

Landmark bootstrap and calibration analyses provided internal validation only. The observed gains were insufficient to establish any index as a clinical prediction tool without external validation.

### CKM stage progression analysis from 2011 to 2015

Among 4,813 participants, 1,402 (29.1%) experienced CKM stage progression between 2011 and 2015; 881 (18.3%) progressed by one stage and 521 (10.8%) by at least two stages. Most indices were positively associated with stage worsening, and 21 showed directionally consistent associations across the progression definitions (Supplementary Fig. S1 and Table S6).

**Fig. 5:**
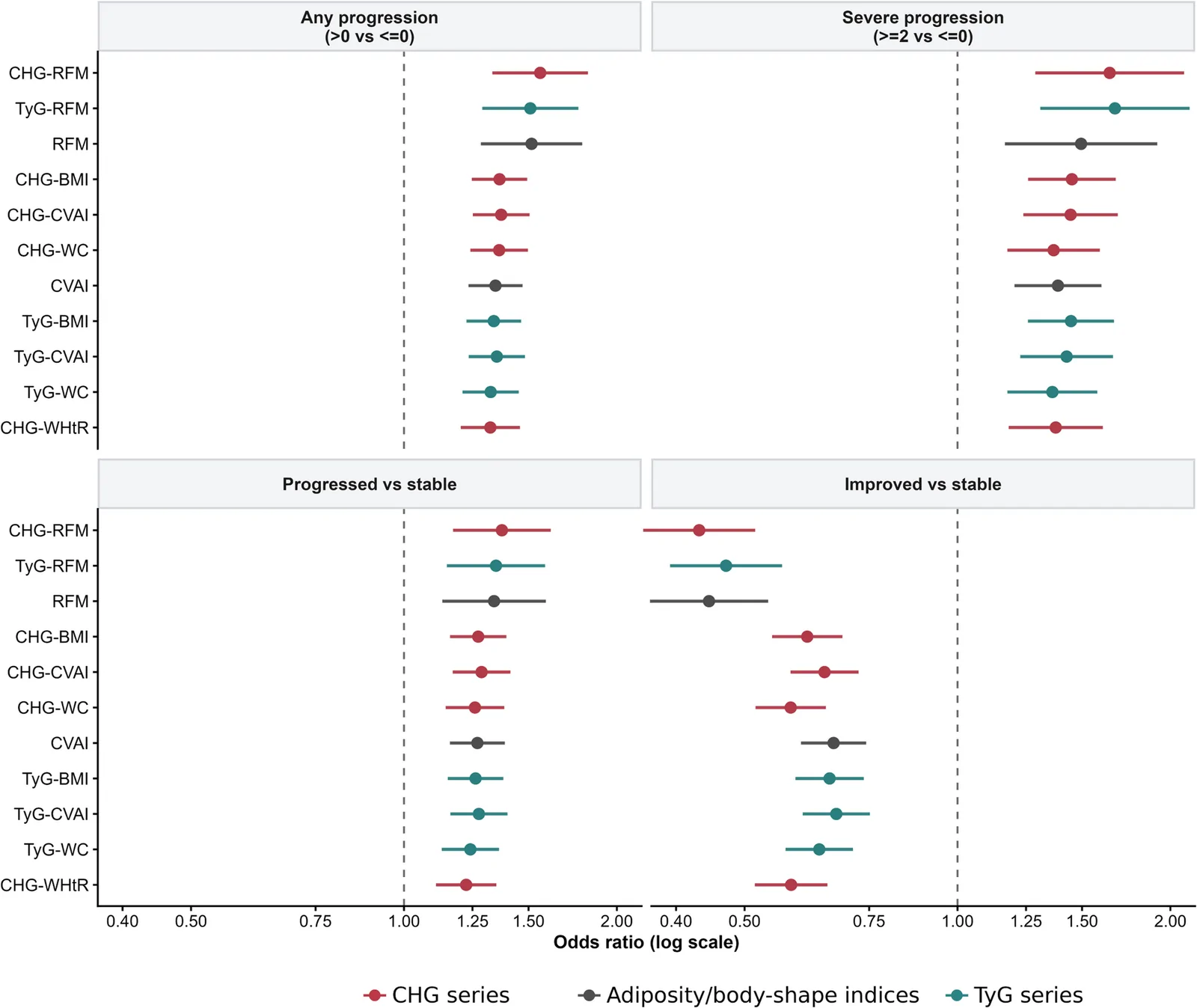
Representative indices and CKM stage progression. The forest plot shows ORs and 95% CIs for the associations between each baseline index and CKM stage progression per 1-SD increment. Any progression was defined as ΔCKM stage > 0. Severe progression was defined as ΔCKM stage ≥ + 2, with ΔCKM stage ≤ 0 as the reference. Directional progression was evaluated using multinomial logistic regression, with stable CKM stage as the reference. The adjusted CKM progression models included age, sex, baseline CKM stage in 2011, education level, marital status, Hukou, smoking status, drinking status, hypertension, diabetes, dyslipidemia, and kidney disease. The x-axis is shown on a logarithmic scale. Abbreviations: OR, odds ratio; CI, confidence interval; CKM, cardiovascular–kidney–metabolic

RFM, CHG-RFM, and TyG-RFM showed comparatively larger estimates across progression contrasts (Fig. 5). Because the indices and CKM staging share metabolic and adiposity components, these findings were considered exploratory rather than evidence of superior prediction or causality.

### Exploratory mediation by CKM stage progression

In the analysis using completed 2015 CKM stage as the mediator, approximately 3,900 participants who met the temporal-order and outcome-ascertainment requirements were included, among whom approximately one-quarter experienced CKM stage progression between 2011 and 2015, and approximately 9% subsequently developed incident CMM after 2015. After restricting the outcome to post-2015 incident CMM, positive associations between various baseline metabolic-related indices and subsequent CMM risk were generally retained.

Exploratory mediation estimates were generally small. CKM stage progression accounted for only a limited proportion of the observed associations and should be viewed as one possible intermediate clinical process rather than a dominant mechanism.

The analysis relied on strong no-unmeasured-confounding assumptions and was intended for hypothesis generation.

Because the mediator incorporated domains that overlap with the exposure indices, mediation estimates may partly reflect mathematical and clinical construct overlap.

**Fig. 6:**
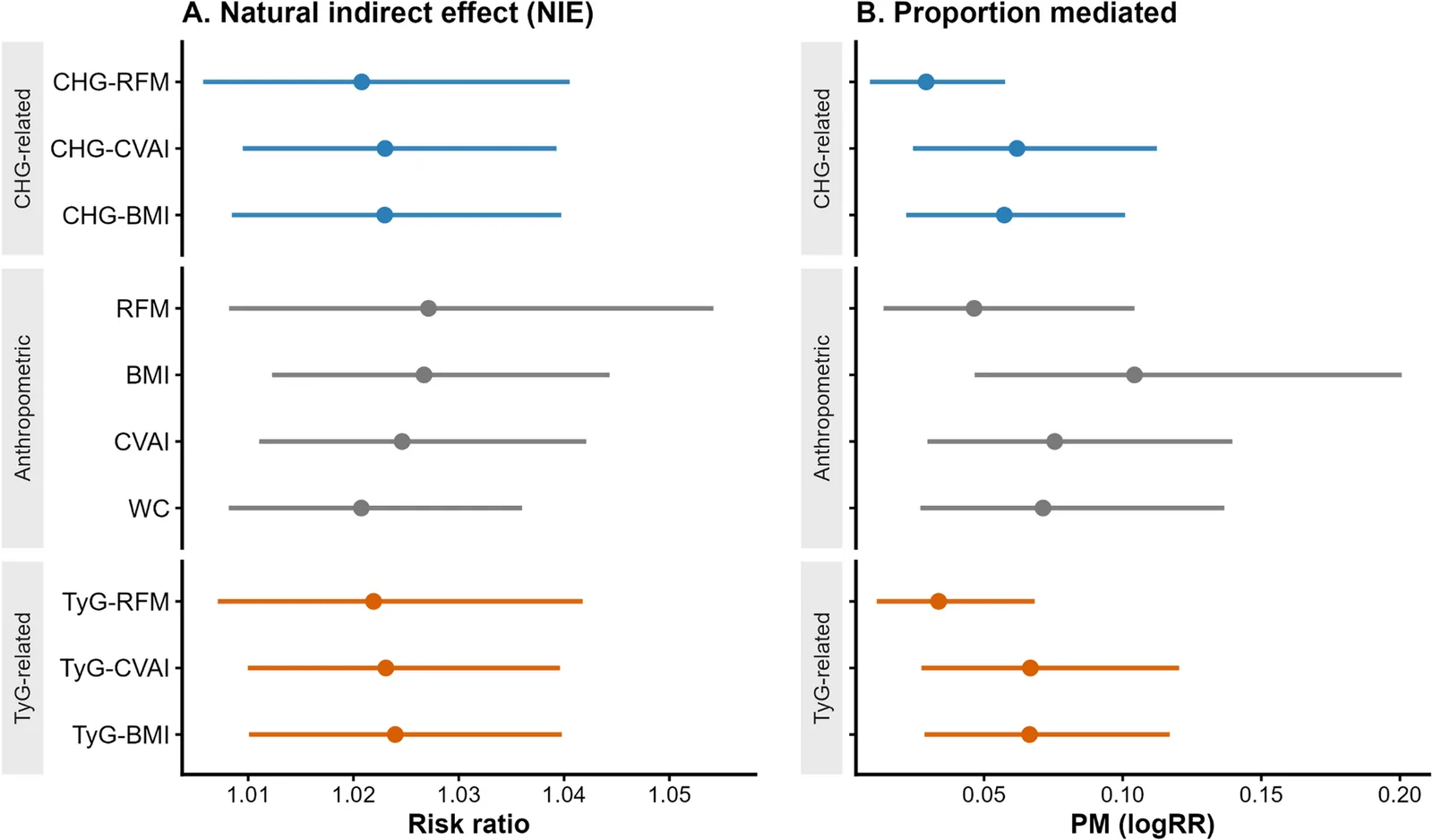
CKM progression mediation for post-2015 CMM. Panel **A** shows natural indirect effects (NIE RRs) and 95% CIs from the main mediation model using the completed 2015 CKM stage definition as the mediator. Panel **B** shows the proportion mediated (PM) on the log-RR scale. Only mediation-prioritized indices are displayed in the main figure; complete results for all 29 candidate indices are provided in Supplementary Fig. S4. Confidence intervals were estimated using 1,000 bootstrap resamples. Abbreviations: CKM, cardiovascular–kidney–metabolic; CMM, cardiometabolic multimorbidity; NIE, natural indirect effect; RR, risk ratio; PM, proportion mediated; CI, confidence interval

Further comparison of different CKM stage definition strategies showed that the completed 2015 CKM stage yielded more conservative estimates, while the overall direction of results and the relative pattern across indices remained similar (Supplementary Fig. S3). The mediation patterns for representative indices are summarized in Fig. 6, with detailed estimates provided in Table 4.

**Table 4:** CKM progression mediation analysis for representative indices

Index	Family	TE RR (95% CI)	NDE RR (95% CI)	NIE RR (95% CI)	PM, % (95% CI)
CHG-BMI	CHG-related	1.486 (1.351–1.652)	1.453 (1.323–1.616)	1.023 (1.008–1.040)	5.7 (2.2–10.1)
CHG-CVAI	CHG-related	1.444 (1.305–1.633)	1.412 (1.279–1.592)	1.023 (1.009–1.039)	6.2 (2.4–11.2)
CHG-RFM	CHG-related	2.027 (1.751–2.357)	1.986 (1.716–2.305)	1.021 (1.006–1.041)	2.9 (0.9–5.8)
WC	Adiposity/body-shape	1.334 (1.198–1.487)	1.307 (1.174–1.461)	1.021 (1.008–1.036)	7.1 (2.7–13.7)
CVAI	Adiposity/body-shape	1.380 (1.238–1.556)	1.347 (1.211–1.522)	1.025 (1.011–1.042)	7.5 (3.0–14.0)
BMI	Adiposity/body-shape	1.288 (1.169–1.424)	1.254 (1.138–1.390)	1.027 (1.012–1.044)	10.4 (4.7–20.1)
RFM	Adiposity/body-shape	1.780 (1.459–2.117)	1.733 (1.426–2.071)	1.027 (1.008–1.054)	4.6 (1.4–10.4)
TyG-BMI	TyG-related	1.428 (1.297–1.590)	1.395 (1.269–1.555)	1.024 (1.010–1.040)	6.6 (2.8–11.7)
TyG-CVAI	TyG-related	1.408 (1.271–1.590)	1.376 (1.244–1.552)	1.023 (1.010–1.040)	6.7 (2.7–12.0)
TyG-RFM	TyG-related	1.905 (1.636–2.224)	1.864 (1.600–2.185)	1.022 (1.007–1.042)	3.4 (1.1–6.8)

Unless otherwise specified, values are RRs and 95% CIs. Results were derived from the main mediation model using the completed 2015 CKM stage definition as the mediator. The model included 3,929 participants and 351 post-2015 incident CMM events. TE, NDE, and NIE represent the total effect, natural direct effect, and natural indirect effect, respectively. PM was calculated on the log-RR scale and is expressed as a percentage. Confidence intervals were estimated using 1,000 bootstrap resamples. Models adjusted for age, sex, education level, marital status, Hukou status, smoking status, drinking status, and baseline CKM stage in 2011

*Abbreviations*: *CMM* cardiometabolic multimorbidity, *CKM* cardiovascular–kidney–metabolic, *RR* risk ratio, *CI* confidence interval, *TE* total effect, *NDE* natural direct effect, *NIE* natural indirect effect, *PM* proportion mediated, *CHG* cholesterol–HDL-C–glucose index, *TyG* triglyceride–glucose index, *BMI* body mass index, *WC* waist circumference, *CVAI* Chinese visceral adiposity index, *RFM* relative fat mass

### Robustness analyses

**Fig. 7:**
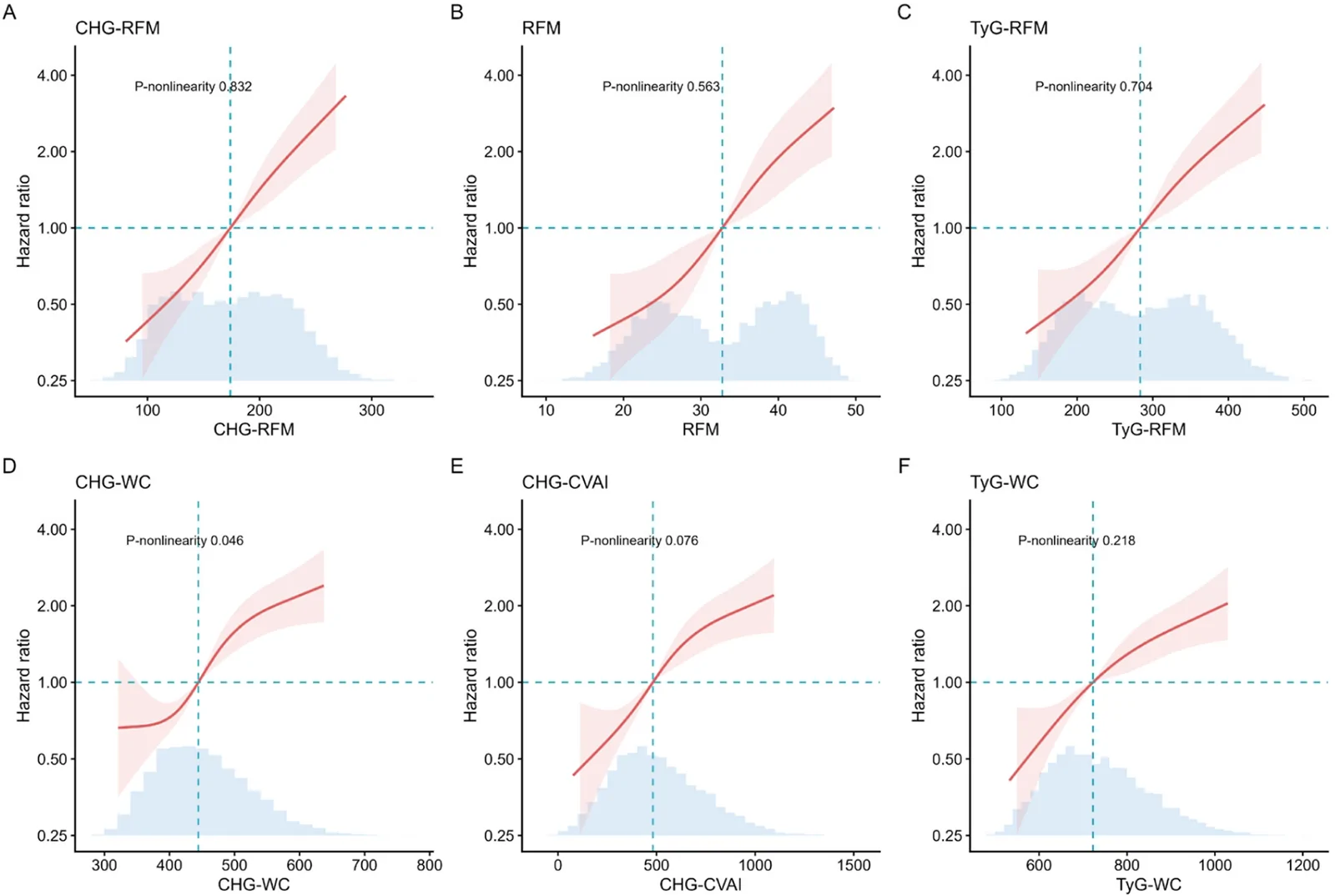
Restricted cubic spline curves for incident CMM. Panels **A**–**F** show CHG-RFM, RFM, TyG-RFM, CHG-WC, CHG-CVAI, and TyG-WC, respectively. Curves were fitted using the extended clinical-adjustment Model 4. The solid line represents the estimated HR, and the shaded area represents the 95% CI. The vertical dashed line indicates the median reference value of each index, and the horizontal dashed line indicates HR = 1. Histograms show the exposure distribution. P values for nonlinearity are nominal values; because none of the nonlinearity tests remained statistically significant after FDR correction, the curves should be interpreted as exploratory dose–response patterns rather than evidence for clinical thresholds. Abbreviations: RCS, restricted cubic spline; HR, hazard ratio; CI, confidence interval; CMM, cardiometabolic multimorbidity; CHG, cholesterol–HDL-C–glucose index; TyG, triglyceride–glucose index; RFM, relative fat mass; WC, waist circumference; CVAI, Chinese visceral adiposity index

In the robustness analyses, most candidate indices showed positive associations in the RCS analysis, with only the overall association for CHG-ABSI appearing slightly weaker. Core indices such as RFM, CHG-RFM, TyG-RFM, as well as CHG-CVAI, CVAI, TyG-BMI, TyG-WC, and TyG-CVAI, showed approximately linear or mildly nonlinear positive associations (Fig. 7), supporting broadly consistent dose–response patterns. Complete dose–response curves for all 29 candidate indices are provided in Supplementary Fig. S8. Although some indices showed borderline nonlinearity in nominal P for nonlinearity tests, none of the nonlinearity signals remained statistically significant after FDR correction. Therefore, these curves should primarily be interpreted as exploratory descriptions rather than confirmatory evidence of thresholds.

After excluding participants with baseline diabetes, all five representative indices remained positively associated with incident CMM among 5,930 participants with 318 events: HRs were 1.97 for RFM, 2.01 for CHG-RFM, 1.82 for TyG-RFM, 1.33 for BMI, and 1.36 for WC (all *P* < 0.001; Supplementary Table S12). In the exploratory CKD-inclusive CMM analysis, corresponding HRs ranged from 1.28 to 1.69 among 6,896 participants with 837 events (Supplementary Table S13).

CKM stage-stratified analyses showed broadly positive directions across baseline stages, although estimates in stage 0 were imprecise because of sparse events (Supplementary Table S3C and Supplementary Fig. S6). These analyses were treated as secondary and were not used to claim identical effects across stages.

Fine–Gray competing-risk analyses were directionally consistent with the main Cox results (Supplementary Table S8). Model 4 also produced broadly similar estimates for the representative indices after wider clinical adjustment. These analyses support the robustness of the overall association direction but do not remove residual confounding, selection, measurement error, or construct overlap.

### Model diagnostics and time-varying associations

Model 3 Schoenfeld diagnostics for the five representative indices are shown in Supplementary Table S7A. Exposure-specific proportional-hazards tests were not significant for RFM or CHG-RFM, but were significant for TyG-RFM, BMI, and WC. In time-varying models, associations were strongest early and attenuated over follow-up: for TyG-RFM, HRs were 2.06, 1.70, and 1.54 at years 1, 4, and 8; for BMI, 1.56, 1.32, and 1.21; and for WC, 1.70, 1.36, and 1.22, respectively (all exposure×log-time *P* < 0.05; Supplementary Table S7B). Thus, a constant HR throughout follow-up was not assumed for these indices; conventional Cox estimates represent average associations over the observed period.

## Discussion

In this prospective cohort of adults with CKM stages 0–3, RFM, CHG-RFM, TyG-RFM, BMI, and WC were associated with incident CMM. Estimates were broadly consistent in the extended Model 4, although its covariates overlap with several index components. Incremental prediction beyond the clinical baseline model was modest.

After BH correction, 28 of the 29 per-SD Model 3 associations remained supported; however, the correlation analysis showed substantial redundancy among structurally related indices. After BH correction, 28 per-SD Model 3 associations remained supported, but the correlation matrix showed that many indices were highly redundant. RFM, CHG-RFM, and TyG-RFM were strongly correlated, and WHtR and BRI were effectively collinear. Accordingly, the study does not provide evidence for 29 independent biomarkers; it describes a smaller number of overlapping metabolic–adiposity phenotypes.

RFM-related indices showed larger association estimates, possibly because RFM integrates waist, height, and sex. However, their high correlations with waist-based constructs and sex-specific formula structure limit claims of superiority; BMI and WC remain simpler to obtain and interpret.

CHG-derived and TyG-derived constructs also showed substantial overlap. In paired Model 3 analyses, CHG and CHG-RFM retained positive coefficients while the matched TyG terms attenuated, but the very high pairwise correlations make these coefficients sensitive to shared components and multicollinearity [43]. The findings are more appropriately interpreted as mutual attenuation within overlapping glucose–lipid phenotypes than as proof that CHG is superior to TyG. CHG remains less extensively validated and requires replication in independent cohorts.

These overlapping associations are biologically plausible but should not be interpreted as mechanistic evidence. TyG-related constructs may reflect insulin-resistance-related glucose–lipid dysfunction [44], whereas waist- and RFM-related measures mainly characterize central or abdominal adiposity [45]. Obesity-related inflammation and adipose-tissue remodeling may further contribute to metabolic deterioration [46, 47], while triglyceride-rich lipoprotein burden and atherosclerotic processes may link these metabolic phenotypes to cardiovascular disease [48, 49]. However, these pathways were not directly measured in CHARLS and should be regarded as contextual biological explanations rather than mechanisms demonstrated by the present study.

Huang et al. evaluated progression from CKM stages 0–2 to stage 3 and reported particularly strong performance for the waist-based TyG-WHtR and TyG-WC indices [30]. Fan et al. examined a CHG–frailty construct in relation to incident heart disease, stroke, diabetes, and CMM [31]. These studies provide relevant context for interpreting waist-based and CHG-derived composite indices in CKM- and CMM-related settings.

The baseline model already achieved an AUC of 0.787, and selected index-extended models reached approximately 0.799–0.800. Although several IDI and NRI estimates were statistically positive, an absolute ΔAUC of about 0.011–0.013 is modest and does not demonstrate a clinically transformative improvement. These indices may be candidate adjunctive markers for preliminary risk recognition, but no decision thresholds or net clinical consequences were validated. Recent studies have applied routinely available metabolic and clinical variables to risk assessment across diverse settings, including CKM progression, cognitive impairment, atrial fibrillation, and cardiovascular outcomes [30, 31, 50–53]. However, differences in populations, outcomes, and baseline models limit direct comparison with incident CMM in the present cohort. However, prediction performance is dependent on the study population, outcome definition, baseline model, and validation strategy. Therefore, the modest AUC improvements observed in the present study should be interpreted cautiously and do not establish these indices as standalone clinical prediction tools.

The model diagnostics further indicate that baseline associations were not necessarily constant. TyG-RFM, BMI, and WC showed exposure-specific proportional-hazards departures, with time-varying HRs declining from year 1 to year 8 while remaining above 1. The main Model 3 Cox estimates should therefore be read as averages across follow-up. Model 4, baseline-diabetes-free, CKD-inclusive, stage-stratified, and competing-risk analyses supported the overall direction of the core findings. CKM progression and mediation analyses remain exploratory because CKM stage shares components with the exposures and outcome, mediated proportions were small, and causal identification assumptions cannot be verified.

Several limitations remain. The observational design precludes causal inference and residual confounding is likely. Heart disease and stroke were largely self-reported, creating possible outcome misclassification. Participants included in the final analytic cohort differed from those not included, particularly in Hukou, education, dyslipidemia, HDL-C, triglycerides, and diabetes, so selection related to data availability may limit generalizability. Medication use was incorporated into disease definitions but treatment may also have modified glucose, lipid, and blood-pressure measurements. The indices, CKM stage, and CMM components overlap conceptually and mathematically. Model 4 further conditioned on several of these overlapping clinical domains and should therefore be interpreted as an extended conditional analysis rather than a definitive causal model. Single baseline measurements do not capture longitudinal changes; exploratory cut-offs were cohort-specific; CHG has limited external validation; and no independent validation cohort was available. These limitations support cautious use of the findings as risk-phenotype evidence rather than clinical decision guidance.

## Conclusions

In conclusion, among adults with CKM stages 0–3, RFM, CHG-RFM, TyG-RFM, BMI, and WC were associated with incident CMM in Model 3, and the overall association pattern remained broadly consistent in Model 4 after wider clinical adjustment. RFM-related indices showed stronger association signals, but the candidate indices were highly overlapping and provided only modest incremental prediction beyond clinical factors. They should be considered candidate adjunctive risk markers—not independent biomarkers or established clinical decision tools—pending external validation.

## Supplementary Information

Additional file 1: Supplementary Tables S1–S13 and Supplementary Figs. S1–S8, including CKM stage definitions, included-versus-not-included comparisons with standardized mean differences, baseline characteristics, formulas for all 29 indices, sequential Cox analyses using Models 1, 2, 3, and 4, CKM stage-stratified analyses, 8-year prediction metrics and exploratory cut-offs, landmark internal validation and calibration, CKM stage progression, Model 3 proportional-hazards diagnostics and time-varying coefficients, Fine–Gray competing-risk analyses, BH-FDR results, inter-index correlations, paired CHG–TyG models, baseline-diabetes-free and CKD-inclusive CMM sensitivity analyses, mediation analyses, subgroup analyses, Kaplan–Meier curves, and restricted cubic spline analyses.

## Data Availability

The datasets used and/or analysed during the current study are available from the China Health and Retirement Longitudinal Study (CHARLS) repository at https://charls.pku.edu.cn/. Access to CHARLS data is subject to registration and approval in accordance with the CHARLS data-use policy. The present study used de-identified publicly available data.

## Abbreviations

ABSI: A body shape index
AUC: Area under the curve
BMI: Body mass index
BRI: Body roundness index
BUN: Blood urea nitrogen
CHARLS: China Health and Retirement Longitudinal Study
CHG: Cholesterol–HDL-C–glucose index
CI: Confidence interval
CKM: Cardiovascular–kidney–metabolic
CMM: Cardiometabolic multimorbidity
cNRI: Continuous net reclassification improvement
CRP: C-reactive protein
CVAI: Chinese visceral adiposity index
DBP: Diastolic blood pressure
eGFR: Estimated glomerular filtration rate
FBG: Fasting blood glucose
GVIF: Generalized variance inflation factor
Hb: Hemoglobin
HbA1c: Hemoglobin A1c
HDL-C: High-density lipoprotein cholesterol
HR: Hazard ratio
IDI: Integrated discrimination improvement
LDL-C: Low-density lipoprotein cholesterol
MCV: Mean corpuscular volume
MICE: Multiple imputation by chained equations
NDE: Natural direct effect
NIE: Natural indirect effect
NRI: Net reclassification improvement
PCV: Packed cell volume
RCS: Restricted cubic spline
RFM: Relative fat mass
SBP: Systolic blood pressure
SD: Standard deviation
TC: Total cholesterol
TG: Triglycerides
TyG: Triglyceride–glucose index
UA: Uric acid
WC: Waist circumference
WBC: White blood cell count
WHtR: Waist-to-height ratio
WWI: Weight-adjusted waist index
