# Supplementary material for: Routine metabolic–adiposity indices and incident cardiometabolic multimorbidity among adults with CKM stages 0–3: a prospective cohort study
Source: BMC Cardiovasc Disord. 2026 Aug 8;26:1031. doi: 10.1186/s12872-026-06385-5 (PMC13732876; doi:10.1186/s12872-026-06385-5)
Supplement: Supplementary file 1 [file 12872_2026_6385_MOESM1_ESM.pdf]

Supplementary Materials

Supplementary Tables

Supplementary Table S1. Operational definition of CKM syndrome stages 0–3 in CHARLS.

Supplementary Table S2A. Baseline characteristics of participants included in the final analytic cohort and those not included.

Supplementary Table S2B. Baseline characteristics according to incident CMM status.

Supplementary Table S2C. Formulas for all 29 candidate indices.

Supplementary Table S3A. Cox summary and multiple-imputation consistency.

Supplementary Table S3B. Sequential Cox analyses using Models 1, 2, 3, and 4.

Supplementary Table S3C. CKM stage-stratified Cox associations.

Supplementary Table S4. Multicollinearity diagnostics for the index-specific Cox models.

Supplementary Table S4A. AUC and IDI at 8 years.

Supplementary Table S4B. NRI and internal-validation metrics.

Supplementary Table S4C. Exploratory cut-offs for 8-year CMM.

Supplementary Table S5. Construction of the landmark cohort.

Supplementary Table S6. CKM stage progression associations.

Supplementary Table S7A. Model 3 Schoenfeld proportional-hazards diagnostics for five representative indices.

Supplementary Table S7B. Model 3 time-varying associations for indices with exposure-specific PH departures.

Supplementary Table S8. Fine–Gray competing-risk analysis.

Supplementary Table S9. BH-FDR-adjusted Model 3 associations across 29 indices.

Supplementary Table S10. Spearman correlation matrix among 29 indices.

Supplementary Table S11. Paired CHG–TyG dual-entry Model 3 analyses.

Supplementary Table S12. Sensitivity analysis excluding baseline diabetes.

Supplementary Table S13. Exploratory CKD-inclusive CMM sensitivity analysis.

Supplementary Figures

Supplementary Fig. S1. CKM progression association heatmap.

Supplementary Fig. S2. Decile-based calibration plots.

Supplementary Fig. S3. Mediation estimates by CKM definition.

Supplementary Fig. S4. Natural indirect effects for all indices.

Supplementary Fig. S5. Age-stratified associations.

Supplementary Fig. S6. CKM stage-stratified associations.

Supplementary Fig. S7A. Kaplan–Meier curves for CHG-related indices.

Supplementary Fig. S7B. Kaplan–Meier curves for adiposity/body-shape indices.

Supplementary Fig. S7C. Kaplan–Meier curves for TyG-related indices.

Supplementary Fig. S8. Dose–response curves for all indices.

Supplementary Table S1. Operational definition of CKM syndrome stages 0–3 in CHARLS

Stage	Operational definition in this study
Stage 0	No CKM risk factors.<br>Defined as not meeting Stage 1, Stage 2, or Stage 3 under the study's strict-completeness rule.
Stage 1	Excess or dysfunctional adiposity (any one of the following):<br>BMI ≥ 24 kg/m^2;<br>Waist circumference ≥ 90 cm in males or ≥ 80 cm in females;<br>Fasting blood glucose 100–125 mg/dL;<br>HbA1c 5.7%–6.4%.
Stage 2	Metabolic risk factors and/or chronic kidney disease (presence of ≥1 of the following five domains):<br>1. Diabetes: fasting blood glucose ≥ 126 mg/dL, HbA1c ≥ 6.5%, self-reported diabetes, or glucose-lowering treatment;<br>2. Hypertension: self-reported hypertension, antihypertensive medication use, or average systolic/diastolic blood pressure ≥ 140/90 mmHg;<br>3. Metabolic syndrome: at least three of the following five components: central obesity (waist circumference ≥ 90 cm in males or ≥ 80 cm in females), fasting blood glucose ≥ 100 mg/dL, blood pressure ≥ 130/80 mmHg or antihypertensive treatment, triglycerides ≥ 150 mg/dL, and low HDL-C (<40 mg/dL in males or <50 mg/dL in females);<br>4. Dyslipidemia: self-reported dyslipidemia, lipid-lowering treatment, or any of the following: total cholesterol ≥ 240 mg/dL, triglycerides ≥ 135 mg/dL, LDL-C ≥ 160 mg/dL, or HDL-C < 40 mg/dL;<br>5. Chronic kidney disease: self-reported kidney disease or eGFR < 60 mL/min/1.73 m^2.
Stage 3	Subclinical cardiovascular disease risk equivalents (any one of the following):<br>eGFR < 30 mL/min/1.73 m^2;<br>10-year Framingham general cardiovascular disease risk ≥ 20%.

Abbreviations: AHA, American Heart Association; CHARLS, China Health and Retirement Longitudinal Study; CKM, cardiovascular–kidney–metabolic; CMM, cardiometabolic multimorbidity; eGFR, estimated glomerular filtration rate; FBG, fasting blood glucose; HbA1c, hemoglobin A1c; HDL-C, high-density lipoprotein cholesterol; LDL-C, low-density lipoprotein cholesterol.

Note. Baseline dyslipidemia used as a model-adjustment covariate was defined using the clinical covariate threshold of TG ≥150 mg/dL, whereas the dyslipidemia component used to operationalize CKM stage 2 followed the CKM staging algorithm and used TG ≥135 mg/dL. These definitions served different analytical purposes and should not be conflated. CKM staging was based on the 2023 American Heart Association CKM framework and was operationalized according to the variables available in CHARLS, with reference to a published CHARLS application among participants with CKM stages 0–3 [1,2].

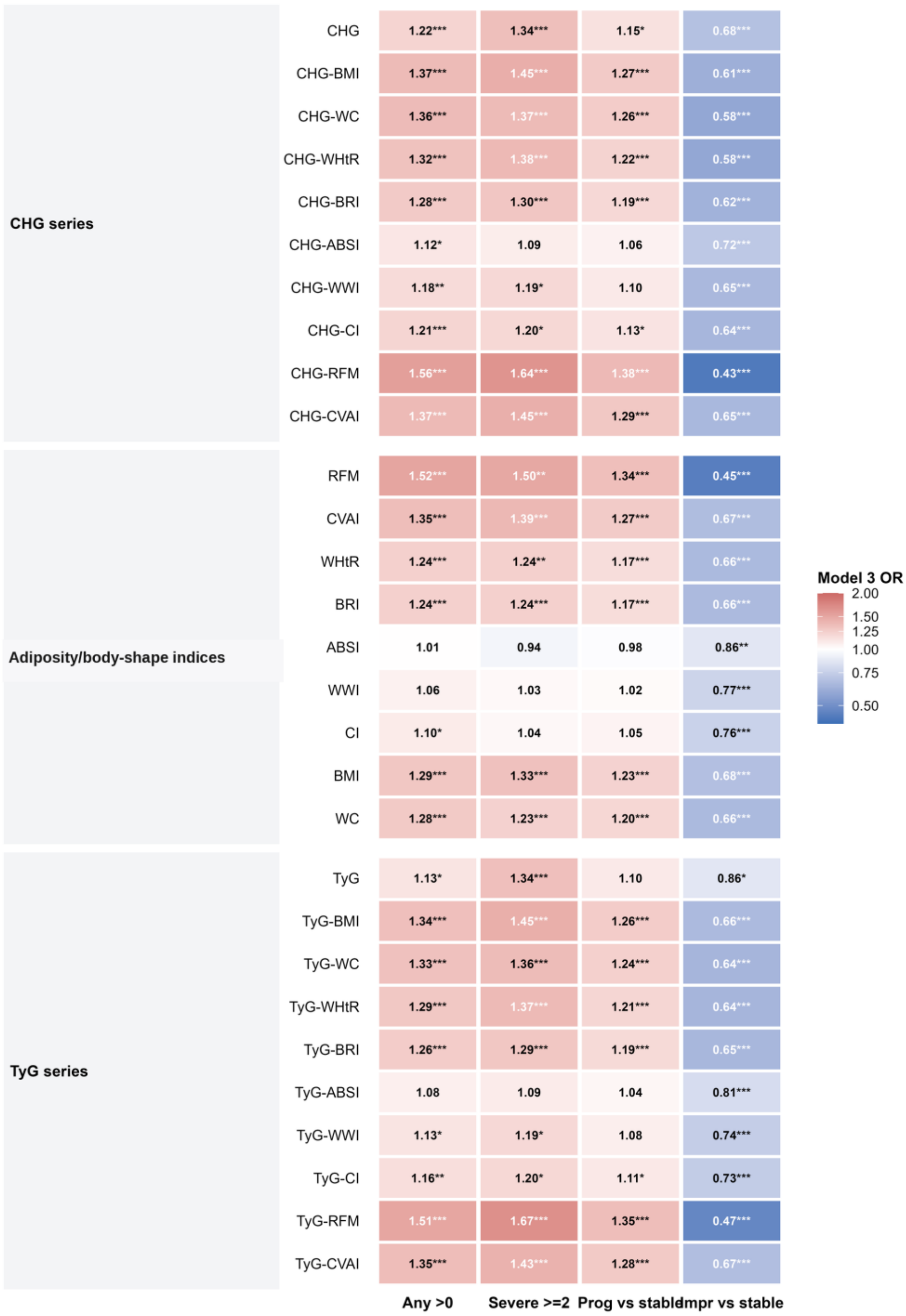

Supplementary Fig. S1. CKM progression association heatmap.

Note. This heatmap summarizes the direction, magnitude, and statistical evidence of the associations between each index and four CKM stage progression contrasts, including any progression, severe progression, progressed vs stable, and improved vs stable. The CKM progression models adjusted for age, sex, baseline CKM stage, education level, marital status, Hukou status, smoking status, drinking status, hypertension, diabetes, dyslipidemia, and kidney disease. Abbreviations: CKM, cardiovascular–kidney–metabolic; OR, odds ratio.

Supplementary Note. Before fitting ordinal logistic regression models, the proportional odds assumption was assessed using the Brant omnibus test. Because all 87 exposure-model combinations violated the proportional odds assumption, extent progression was analyzed primarily using multinomial logistic regression.

Supplementary Table S2A. Baseline characteristics of participants included in the final analytic cohort and those not included

Variable	Total baseline sample, N=17,708	Not included, N=10,646	Final analytic cohort, N=7,062	SMD
N	17,708	10,646	7,062	
Age, years	59 ± 10	59 ± 11	59 ± 9	0.00
**Sex**				**0.01**
Male	8,471 (48%)	5,112 (48%)	3,359 (48%)	
Female	9,221 (52%)	5,518 (52%)	3,703 (52%)	
**Marital status**				**0.08**
Married	15,417 (87%)	9,147 (86%)	6,270 (89%)	
Others	2,258 (13%)	1,466 (14%)	792 (11%)	
**Education level**				**0.20**
Secondary school or below	15,407 (87%)	8,997 (85%)	6,410 (91%)	
High school	1,817 (10%)	1,239 (12%)	578 (8.2%)	
College or above	429 (2.4%)	355 (3.4%)	74 (1.0%)	
**Hukou**				**0.35**
Agriculture	13,694 (77%)	7,633 (72%)	6,061 (86%)	
Others	3,979 (23%)	2,978 (28%)	1,001 (14%)	
**Smoking status (%)**				**0.01**
No	10,619 (60%)	6,367 (61%)	4,252 (60%)	
Yes	6,940 (40%)	4,130 (39%)	2,810 (40%)	
**Drinking status (%)**				**0.03**
No	12,116 (69%)	7,305 (70%)	4,811 (68%)	
Yes	5,426 (31%)	3,179 (30%)	2,247 (32%)	
**Hypertension (%)**				**0.01**
No	11,015 (63%)	6,540 (63%)	4,475 (63%)	
Yes	6,484 (37%)	3,897 (37%)	2,587 (37%)	
**Diabetes (%)**				**0.11**
No	15,103 (87%)	9,137 (88%)	5,966 (84%)	
Yes	2,330 (13%)	1,234 (12%)	1,096 (16%)	
**Dyslipidemia (%)**				**0.31**
No	11,165 (64%)	7,242 (70%)	3,923 (56%)	
Yes	6,185 (36%)	3,046 (30%)	3,139 (44%)	
**Kidney disease (%)**				**0.02**
No	16,148 (93%)	9,595 (92%)	6,553 (93%)	
Yes	1,306 (7.5%)	797 (7.7%)	509 (7.2%)	
SBP, mmHg	130 ± 21	130 ± 22	129 ± 21	0.05
DBP, mmHg	75 ± 12	76 ± 12	75 ± 12	0.08
FBG, mg/dL	102 (94, 114)	103 (95, 115)	102 (94, 113)	0.07
HbA1c, %	5.10 (4.90, 5.40)	5.10 (4.90, 5.40)	5.10 (4.90, 5.40)	0.00
TC, mg/dL	193 ± 39	192 ± 40	193 ± 37	0.03
HDL-C, mg/dL	51 ± 15	49 ± 15	52 ± 15	0.20
LDL-C, mg/dL	116 ± 34	116 ± 34	116 ± 34	0.00
TG, mg/dL	106 (75, 157)	111 (79, 164)	104 (73, 150)	0.12

Variable	Total baseline sample, N=17,708	Not included, N=10,646	Final analytic cohort, N=7,062	SMD
eGFR, mL/min/1.73 m²	105 (90, 123)	104 (88, 122)	105 (90, 123)	0.04
Uric acid, mg/dL	4.46 ± 1.27	4.51 ± 1.31	4.43 ± 1.20	0.06
CRP, mg/L	1.04 (0.55, 2.19)	1.11 (0.58, 2.40)	0.99 (0.53, 2.06)	0.10
BUN, mg/dL	15.7 ± 4.6	15.7 ± 4.9	15.7 ± 4.3	0.00
Creatinine, mg/dL	0.78 ± 0.24	0.79 ± 0.25	0.78 ± 0.17	0.05

Note. Values are mean ± SD, median (IQR), or n (%). SMD compares the final analytic cohort with participants not included. For variables summarized as mean ± SD, SMD used the pooled SD; for skewed variables summarized as median (IQR), a robust SMD was approximated using IQR/1.349; for categorical variables, a multilevel standardized difference was calculated from category proportions. Absolute SMD ≥0.10 indicates potentially meaningful imbalance. Denominators vary slightly because of item-level missingness. Comparisons are descriptive and do not imply hypothesis testing. Abbreviations: SMD, standardized mean difference; SD, standard deviation; IQR, interquartile range; SBP, systolic blood pressure; DBP, diastolic blood pressure; FBG, fasting blood glucose; HbA1c, hemoglobin A1c; TC, total cholesterol; HDL-C, high-density lipoprotein cholesterol; LDL-C, low-density lipoprotein cholesterol; TG, triglycerides; eGFR, estimated glomerular filtration rate; CRP, C-reactive protein; BUN, blood urea nitrogen. The source population was derived from the 2011 baseline wave of the China Health and Retirement Longitudinal Study [3].

Supplementary Table S2B. Baseline characteristics according to incident CMM status

	Incident CMM during follow-up			
**Variable**	**Overall, N = 7062[a]**	**No, N = 6,449[a]**	**Yes, N = 613[a]**	**P value[b]**
N	7062	6449	613	
Age, years	59 ± 9	59 ± 9	60 ± 8	<0.001
Sex				0.006
Male	3359 (48%)	3100 (48%)	259 (42%)	
Female	3703 (52%)	3349 (52%)	354 (58%)	
Marital status				0.973
Married	6270 (89%)	5726 (89%)	544 (89%)	
Others	792 (11%)	723 (11%)	69 (11%)	
Education level				0.219
Secondary school or below	6410 (91%)	5857 (91%)	553 (90%)	
High school	578 (8.2%)	521 (8.1%)	57 (9.3%)	
College or above	74 (1.0%)	71 (1.1%)	3 (0.5%)	
Hukou				0.326
Agriculture	6061 (86%)	5543 (86%)	518 (85%)	
Others	1001 (14%)	906 (14%)	95 (15%)	
Smoking status (%)				0.671
No	4252 (60%)	3878 (60%)	374 (61%)	
Yes	2810 (40%)	2571 (40%)	239 (39%)	
Drinking status (%)				0.357
No	4811 (68%)	4383 (68%)	428 (70%)	
Yes	2247 (32%)	2062 (32%)	185 (30%)	
Hypertension (%)				<0.001
No	4475 (63%)	4213 (65%)	262 (43%)	
Yes	2587 (37%)	2236 (35%)	351 (57%)	
Diabetes (%)				<0.001
No	5966 (84%)	5616 (87%)	350 (57%)	
Yes	1096 (16%)	833 (13%)	263 (43%)	
Dyslipidemia (%)				<0.001
No	3923 (56%)	3686 (57%)	237 (39%)	
Yes	3139 (44%)	2763 (43%)	376 (61%)	
Kidney disease (%)				0.036
No	6553 (93%)	5997 (93%)	556 (91%)	
Yes	509 (7.2%)	452 (7.0%)	57 (9.3%)	
SBP, mmHg	129 ± 21	128 ± 21	136 ± 21	<0.001
DBP, mmHg	75 ± 12	75 ± 12	78 ± 12	<0.001

	Incident CMM during follow-up			
**Variable**	**Overall, N = 7062**[a]	**No, N = 6,449**[a]	**Yes, N = 613**[a]	**P value**[b]
FBG, mg/dL	102 (94, 113)	102 (94, 111)	113 (100, 137)	<0.001
HbA1c, %	5.10 (4.90, 5.40)	5.10 (4.90, 5.40)	5.30 (5.00, 5.80)	<0.001
TC, mg/dL	193 ± 37	192 ± 36	202 ± 39	<0.001
HDL-C, mg/dL	52 ± 15	52 ± 15	48 ± 15	<0.001
LDL-C, mg/dL	116 ± 34	116 ± 33	122 ± 37	<0.001
TG, mg/dL	104 (73, 150)	102 (73, 148)	125 (89, 198)	<0.001
eGFR, mL/min/1.73 m²	105 (90, 123)	105 (90, 123)	104 (90, 118)	0.018
Uric acid, mg/dL	4.43 ± 1.20	4.42 ± 1.20	4.48 ± 1.28	0.325
CRP, mg/L	0.99 (0.53, 2.06)	0.95 (0.53, 1.97)	1.40 (0.66, 2.75)	<0.001
BUN, mg/dL	15.7 ± 4.3	15.7 ± 4.3	15.6 ± 4.3	0.304
Creatinine, mg/dL	0.78 ± 0.17	0.78 ± 0.17	0.78 ± 0.17	0.731

[a] Values are presented as mean ± SD, median (IQR), or n (%), as appropriate.

[b] Age, SBP, DBP, TC, HDL-C, LDL-C, uric acid, BUN, and creatinine were compared using Welch's t-test. FBG, HbA1c, TG, eGFR, and CRP were compared using the Wilcoxon rank-sum test. Categorical variables were compared using Pearson's chi-square test or Fisher's exact test, as appropriate. CMM, cardiometabolic multimorbidity; FBG, fasting blood glucose; SBP, systolic blood pressure; DBP, diastolic blood pressure; TC, total cholesterol; HDL-C, high-density lipoprotein cholesterol; LDL-C, low-density lipoprotein cholesterol; TG, triglycerides; eGFR, estimated glomerular filtration rate; CRP, C-reactive protein; BUN, blood urea nitrogen; IQR, interquartile range.

Supplementary Table S2C. Formulas for all 29 candidate indices

Index	Formula
CHG	ln[TC (mmol/L) × FBG (mg/dL)/(2 × HDL-C (mmol/L))]
CHG-BMI	CHG × BMI
CHG-WC	CHG × WC
CHG-WHtR	CHG × WHtR
CHG-BRI	CHG × BRI
CHG-ABSI	CHG × ABSI
CHG-WWI	CHG × WWI
CHG-CI	CHG × CI
CHG-RFM	CHG × RFM
CHG-CVAI	CHG × CVAI
RFM	Male: 64 − 20 × height/WC; Female: 76 − 20 × height/WC (height and WC in m)
CVAI	Male: −267.93 + 0.68 × age (years) + 0.03 × BMI (kg/m^2) + 4.00 × WC (cm) + 22.00 × log10[TG (mmol/L)] − 16.32 × HDL-C (mmol/L); Female: −187.32 + 1.71 × age (years) + 4.23 × BMI (kg/m^2) + 1.12 × WC (cm) + 39.76 × log10[TG (mmol/L)] − 11.66 × HDL-C (mmol/L)
WHtR	WC (cm)/height (cm)
BRI	364.2 − 365.5 × √{1 − [WC (m)/(π × height (m))]^2}
ABSI	WC (m)/[BMI^(2/3) × height (m)^(1/2)]
WWI	WC (cm)/√weight (kg)
CI	WC (m)/[0.109 × √(weight (kg)/height (m))]
BMI	weight (kg)/height (m)^2
WC	Measured waist circumference (cm)
TyG	ln[TG (mg/dL) × FBG (mg/dL)/2]
TyG-BMI	TyG × BMI
TyG-WC	TyG × WC
TyG-WHtR	TyG × WHtR
TyG-BRI	TyG × BRI
TyG-ABSI	TyG × ABSI
TyG-WWI	TyG × WWI
TyG-CI	TyG × CI
TyG-RFM	TyG × RFM
TyG-CVAI	TyG × CVAI

Note. CVAI was calculated using the published sex-specific equations. Before index construction, continuous components were winsorized at the 1st and 99th percentiles. Abbreviations: CHG, cholesterol–HDL-C–glucose index; TyG, triglyceride–glucose index; TC, total cholesterol; FBG, fasting blood glucose; HDL-C, high-density lipoprotein cholesterol; TG, triglycerides; BMI, body mass index; WC, waist circumference; WHtR, waist-to-height ratio; BRI, body roundness index; ABSI, a body shape index; WWI, weight-adjusted waist index; CI, conicity index; RFM, relative fat mass; CVAI, Chinese visceral adiposity index.

S3. Sequential Cox associations and CKM stage-stratified analyses

Supplementary Tables S3A–S3C provide the Cox summary and multiple-imputation consistency results, sequential Cox results for all candidate indices using Models 1, 2, 3, and 4, and CKM stage-stratified results, respectively.

Supplementary Table S3A. Model 3 and Model 4 Cox estimates and multiple-imputation consistency

Family	Marker	Model 3 CC per-SD HR (95% CI)	Model 4 CC per-SD HR (95% CI)	Model 4 MI per-SD HR (95% CI)	Presentation group
**CHG-related**					
CHG-related	CHG	1.196 (1.082–1.321)	1.172 (1.025–1.340)	1.170 (1.023–1.339)	Supplementary marker
CHG-related	CHG-ABSI	1.160 (1.057–1.273)	1.115 (1.002–1.242)	1.115 (1.002–1.242)	Supplementary marker
CHG-related	CHG-BMI	1.349 (1.238–1.470)	1.377 (1.248–1.520)	1.376 (1.246–1.519)	Consistent association marker
CHG-related	CHG-BRI	1.327 (1.217–1.448)	1.315 (1.197–1.444)	1.314 (1.196–1.444)	Supplementary marker
CHG-related	CHG-CI	1.250 (1.136–1.375)	1.231 (1.100–1.378)	1.231 (1.100–1.377)	Supplementary marker
CHG-related	CHG-CVAI	1.346 (1.228–1.475)	1.411 (1.264–1.575)	1.409 (1.262–1.573)	Consistent association marker
CHG-related	CHG-RFM	1.724 (1.465–2.028)	1.781 (1.473–2.153)	1.779 (1.471–2.152)	Representative index
CHG-related	CHG-WC	1.373 (1.252–1.505)	1.414 (1.268–1.576)	1.412 (1.266–1.575)	Consistent association marker
CHG-related	CHG-WHtR	1.373 (1.248–1.510)	1.394 (1.248–1.557)	1.393 (1.247–1.557)	Consistent association marker
CHG-related	CHG-WWI	1.243 (1.126–1.372)	1.210 (1.079–1.357)	1.210 (1.079–1.357)	Supplementary marker
**Adiposity/body-shape**					
Adiposity/body-shape	ABSI	1.054 (0.965–1.151)	1.043 (0.954–1.141)	1.043 (0.954–1.141)	Supplementary marker
Adiposity/body-shape	BMI	1.303 (1.203–1.412)	1.302 (1.195–1.419)	1.301 (1.194–1.418)	Representative index
Adiposity/body-shape	BRI	1.313 (1.203–1.432)	1.293 (1.181–1.416)	1.293 (1.181–1.416)	Supplementary marker
Adiposity/body-shape	CI	1.172 (1.072–1.281)	1.149 (1.050–1.258)	1.149 (1.050–1.258)	Supplementary marker
Adiposity/body-shape	CVAI	1.361 (1.238–1.496)	1.405 (1.260–1.566)	1.403 (1.258–1.565)	Consistent association marker
Adiposity/body-shape	RFM	1.846 (1.517–2.247)	1.793 (1.460–2.203)	1.793 (1.459–2.203)	Representative index
Adiposity/body-shape	WC	1.337 (1.225–1.459)	1.329 (1.212–1.457)	1.328 (1.211–1.457)	Representative index
Adiposity/body-shape	WHtR	1.335 (1.218–1.464)	1.315 (1.195–1.448)	1.315 (1.195–1.448)	Supplementary marker
Adiposity/body-shape	WWI	1.160 (1.054–1.278)	1.135 (1.029–1.251)	1.135 (1.029–1.252)	Supplementary marker
**TyG-related**					
TyG-related	TyG	1.112 (1.008–1.226)	1.139 (1.019–1.273)	1.133 (1.013–1.266)	Supplementary marker
TyG-related	TyG-ABSI	1.104 (1.007–1.210)	1.105 (1.002–1.219)	1.102 (0.999–1.216)	Supplementary marker
TyG-related	TyG-BMI	1.323 (1.214–1.443)	1.355 (1.233–1.490)	1.352 (1.230–1.487)	Consistent association marker
TyG-related	TyG-BRI	1.310 (1.201–1.430)	1.306 (1.190–1.433)	1.305 (1.189–1.432)	Supplementary marker
TyG-related	TyG-CI	1.191 (1.084–1.309)	1.200 (1.084–1.330)	1.197 (1.080–1.326)	Supplementary marker
TyG-related	TyG-CVAI	1.328 (1.211–1.456)	1.391 (1.248–1.549)	1.388 (1.246–1.547)	Consistent association marker
TyG-related	TyG-RFM	1.671 (1.412–1.977)	1.729 (1.437–2.082)	1.724 (1.432–2.076)	Representative index

Family	Marker	Model 3 CC per-SD HR (95% CI)	Model 4 CC per-SD HR (95% CI)	Model 4 MI per-SD HR (95% CI)	Presentation group
TyG-related	TyG-WC	1.335 (1.217–1.465)	1.374 (1.240–1.523)	1.370 (1.236–1.518)	Consistent association marker
TyG-related	TyG-WHtR	1.334 (1.212–1.468)	1.357 (1.222–1.507)	1.354 (1.219–1.504)	Supplementary marker
TyG-related	TyG-WWI	1.184 (1.073–1.306)	1.185 (1.067–1.317)	1.182 (1.064–1.313)	Supplementary marker

Note. This table summarizes per-SD associations for all 29 candidate indices in Model 3 and Model 4. The descriptive cohort comprised 7,062 participants with 613 incident CMM events, whereas the complete-case Model 3 Cox analyses comprised 7,014 participants with 569 events after applying the complete-case requirements of the Cox analysis. Complete-case estimates were derived from the corresponding Cox proportional hazards models, whereas multiple-imputation estimates were used only to assess the sensitivity of Model 4 results to missing baseline covariates. Each index was entered separately. Model 3 adjusted for age, sex, education level, marital status, Hukou, smoking status, drinking status, hypertension, diabetes, dyslipidemia, and kidney disease. Model 4 additionally adjusted for baseline CKM stage, SBP, DBP, HDL-C, LDL-C, eGFR, uric acid, BUN, hemoglobin, MCV, PCV, platelet count, and white blood cell count. Model 3 anchored the primary association interpretation, whereas Model 4 was retained as an extended clinical-adjustment analysis. Presentation groups were used to organize the results and were not intended to imply formal superiority testing.

Supplementary Table S3B. Sequential Cox analyses using Models 1, 2, 3, and 4

Family	Marker	Scale / category	Model 1	Model 2	Model 3	Model 4
**CHG-related**						
CHG-related	CHG	Per SD	1.74 (1.63–1.86)<br>P < 0.001	1.74 (1.63–1.86)<br>P < 0.001	1.20 (1.08–1.32)<br>P < 0.001	1.17 (1.02–1.34)<br>P = 0.021
		Q1	1.00 (Ref)	1.00 (Ref)	1.00 (Ref)	1.00 (Ref)
		Q2	1.15 (0.83–1.58)<br>P = 0.401	1.15 (0.84–1.59)<br>P = 0.378	1.06 (0.77–1.46)<br>P = 0.731	0.95 (0.67–1.35)<br>P = 0.788
		Q3	1.94 (1.45–2.59)<br>P < 0.001	1.96 (1.47–2.61)<br>P < 0.001	1.42 (1.04–1.93)<br>P = 0.027	1.22 (0.84–1.76)<br>P = 0.303
		Q4	4.23 (3.25–5.50)<br>P < 0.001	4.27 (3.28–5.56)<br>P < 0.001	1.88 (1.34–2.63)<br>P < 0.001	1.58 (1.02–2.46)<br>P = 0.042
		Trend test	1.72 (1.59–1.87)<br>P for trend < 0.001	1.73 (1.59–1.88)<br>P for trend < 0.001	1.26 (1.13–1.40)<br>P for trend < 0.001	1.20 (1.04–1.39)<br>P for trend = 0.012
CHG-related	CHG-BMI	Per SD	1.72 (1.61–1.85)<br>P < 0.001	1.74 (1.62–1.86)<br>P < 0.001	1.35 (1.24–1.47)<br>P < 0.001	1.38 (1.25–1.52)<br>P < 0.001
		Q1	1.00 (Ref)	1.00 (Ref)	1.00 (Ref)	1.00 (Ref)
		Q2	1.31 (0.95–1.79)<br>P = 0.095	1.34 (0.98–1.84)<br>P = 0.069	1.09 (0.79–1.50)<br>P = 0.586	1.06 (0.77–1.48)<br>P = 0.713
		Q3	2.16 (1.62–2.88)<br>P < 0.001	2.23 (1.67–2.98)<br>P < 0.001	1.48 (1.10–2.01)<br>P = 0.011	1.44 (1.04–2.00)<br>P = 0.028
		Q4	4.40 (3.37–5.74)<br>P < 0.001	4.60 (3.51–6.02)<br>P < 0.001	2.23 (1.64–3.02)<br>P < 0.001	2.23 (1.57–3.15)<br>P < 0.001
		Trend test	1.71 (1.57–1.85)<br>P for trend < 0.001	1.73 (1.59–1.88)<br>P for trend < 0.001	1.35 (1.22–1.48)<br>P for trend < 0.001	1.36 (1.22–1.51)<br>P for trend < 0.001
CHG-related	CHG-WC	Per SD	1.84 (1.71–1.98)<br>P < 0.001	1.85 (1.72–1.99)<br>P < 0.001	1.37 (1.25–1.51)<br>P < 0.001	1.41 (1.27–1.58)<br>P < 0.001
		Q1	1.00 (Ref)	1.00 (Ref)	1.00 (Ref)	1.00 (Ref)
		Q2	1.30 (0.94–1.81)<br>P = 0.116	1.32 (0.95–1.84)<br>P = 0.101	1.11 (0.79–1.55)<br>P = 0.547	1.05 (0.74–1.49)<br>P = 0.775
		Q3	2.45 (1.82–3.29)<br>P < 0.001	2.50 (1.86–3.36)<br>P < 0.001	1.79 (1.31–2.44)<br>P < 0.001	1.69 (1.20–2.37)<br>P = 0.003
		Q4	4.70 (3.57–6.19)<br>P < 0.001	4.80 (3.64–6.34)<br>P < 0.001	2.32 (1.69–3.18)<br>P < 0.001	2.29 (1.59–3.29)<br>P < 0.001
		Trend test	1.76 (1.62–1.91)<br>P for trend < 0.001	1.77 (1.62–1.92)<br>P for trend < 0.001	1.36 (1.24–1.50)<br>P for trend < 0.001	1.37 (1.23–1.54)<br>P for trend < 0.001

Family	Marker	Scale / category	Model 1	Model 2	Model 3	Model 4
CHG-related	CHG-WHtR	Per SD	1.87 (1.74–2.02)<br>P < 0.001	1.89 (1.75–2.04)<br>P < 0.001	1.37 (1.25–1.51)<br>P < 0.001	1.39 (1.25–1.56)<br>P < 0.001
		Q1	1.00 (Ref)	1.00 (Ref)	1.00 (Ref)	1.00 (Ref)
		Q2	1.48 (1.05–2.08)<br>P = 0.024	1.51 (1.07–2.12)<br>P = 0.018	1.24 (0.88–1.75)<br>P = 0.215	1.19 (0.83–1.70)<br>P = 0.343
		Q3	2.85 (2.09–3.88)<br>P < 0.001	2.92 (2.14–3.97)<br>P < 0.001	1.98 (1.44–2.73)<br>P < 0.001	1.85 (1.30–2.64)<br>P < 0.001
		Q4	5.22 (3.89–7.01)<br>P < 0.001	5.40 (4.02–7.26)<br>P < 0.001	2.50 (1.79–3.49)<br>P < 0.001	2.44 (1.67–3.58)<br>P < 0.001
		Trend test	1.79 (1.64–1.95)<br>P for trend < 0.001	1.81 (1.66–1.97)<br>P for trend < 0.001	1.38 (1.25–1.52)<br>P for trend < 0.001	1.38 (1.23–1.55)<br>P for trend < 0.001
CHG-related	CHG-BRI	Per SD	1.72 (1.59–1.85)<br>P < 0.001	1.74 (1.61–1.87)<br>P < 0.001	1.33 (1.22–1.45)<br>P < 0.001	1.31 (1.20–1.44)<br>P < 0.001
		Q1	1.00 (Ref)	1.00 (Ref)	1.00 (Ref)	1.00 (Ref)
		Q2	1.82 (1.32–2.52)<br>P < 0.001	1.84 (1.33–2.53)<br>P < 0.001	1.56 (1.13–2.16)<br>P = 0.007	1.50 (1.08–2.09)<br>P = 0.017
		Q3	2.50 (1.83–3.40)<br>P < 0.001	2.54 (1.87–3.47)<br>P < 0.001	1.75 (1.28–2.41)<br>P < 0.001	1.66 (1.19–2.31)<br>P = 0.003
		Q4	4.97 (3.70–6.67)<br>P < 0.001	5.12 (3.81–6.88)<br>P < 0.001	2.59 (1.89–3.56)<br>P < 0.001	2.49 (1.77–3.50)<br>P < 0.001
		Trend test	1.69 (1.55–1.84)<br>P for trend < 0.001	1.71 (1.57–1.86)<br>P for trend < 0.001	1.34 (1.22–1.48)<br>P for trend < 0.001	1.33 (1.20–1.47)<br>P for trend < 0.001
CHG-related	CHG-ABSI	Per SD	1.67 (1.56–1.80)<br>P < 0.001	1.67 (1.55–1.79)<br>P < 0.001	1.16 (1.06–1.27)<br>P = 0.002	1.12 (1.00–1.24)<br>P = 0.046
		Q1	1.00 (Ref)	1.00 (Ref)	1.00 (Ref)	1.00 (Ref)
		Q2	1.95 (1.41–2.69)<br>P < 0.001	1.96 (1.42–2.71)<br>P < 0.001	1.66 (1.20–2.31)<br>P = 0.002	1.53 (1.10–2.14)<br>P = 0.012
		Q3	2.59 (1.90–3.53)<br>P < 0.001	2.62 (1.92–3.57)<br>P < 0.001	1.81 (1.31–2.49)<br>P < 0.001	1.54 (1.09–2.17)<br>P = 0.014
		Q4	4.70 (3.50–6.31)<br>P < 0.001	4.72 (3.51–6.34)<br>P < 0.001	1.98 (1.41–2.77)<br>P < 0.001	1.67 (1.15–2.42)<br>P = 0.007
		Trend test	1.63 (1.50–1.77)<br>P for trend < 0.001	1.63 (1.50–1.77)<br>P for trend < 0.001	1.20 (1.09–1.32)<br>P for trend < 0.001	1.13 (1.01–1.26)<br>P for trend = 0.028
CHG-related	CHG-WWI	Per SD	1.80 (1.67–1.94)<br>P < 0.001	1.80 (1.67–1.94)<br>P < 0.001	1.24 (1.13–1.37)<br>P < 0.001	1.21 (1.08–1.36)<br>P = 0.001
		Q1	1.00 (Ref)	1.00 (Ref)	1.00 (Ref)	1.00 (Ref)

Family	Marker	Scale / category	Model 1	Model 2	Model 3	Model 4
		Q2	1.69 (1.21–2.37)<br>P = 0.002	1.71 (1.22–2.39)<br>P = 0.002	1.38 (0.98–1.94)<br>P = 0.062	1.28 (0.90–1.82)<br>P = 0.165
		Q3	2.91 (2.13–3.98)<br>P < 0.001	2.96 (2.16–4.04)<br>P < 0.001	2.00 (1.44–2.77)<br>P < 0.001	1.75 (1.23–2.49)<br>P = 0.002
		Q4	5.22 (3.86–7.06)<br>P < 0.001	5.29 (3.91–7.16)<br>P < 0.001	2.15 (1.52–3.04)<br>P < 0.001	1.88 (1.28–2.77)<br>P = 0.001
		Trend test	1.74 (1.60–1.90)<br>P for trend < 0.001	1.75 (1.60–1.91)<br>P for trend < 0.001	1.28 (1.15–1.41)<br>P for trend < 0.001	1.22 (1.09–1.37)<br>P for trend < 0.001
CHG-related	CHG-CI	Per SD	1.78 (1.66–1.92)<br>P < 0.001	1.78 (1.65–1.91)<br>P < 0.001	1.25 (1.14–1.38)<br>P < 0.001	1.23 (1.10–1.38)<br>P < 0.001
		Q1	1.00 (Ref)	1.00 (Ref)	1.00 (Ref)	1.00 (Ref)
		Q2	1.54 (1.10–2.17)<br>P = 0.013	1.56 (1.10–2.19)<br>P = 0.011	1.32 (0.93–1.86)<br>P = 0.120	1.22 (0.86–1.75)<br>P = 0.269
		Q3	2.87 (2.10–3.91)<br>P < 0.001	2.90 (2.13–3.96)<br>P < 0.001	2.01 (1.45–2.78)<br>P < 0.001	1.81 (1.27–2.58)<br>P = 0.001
		Q4	5.22 (3.88–7.01)<br>P < 0.001	5.25 (3.90–7.06)<br>P < 0.001	2.36 (1.68–3.31)<br>P < 0.001	2.15 (1.46–3.17)<br>P < 0.001
		Trend test	1.77 (1.63–1.93)<br>P for trend < 0.001	1.78 (1.63–1.93)<br>P for trend < 0.001	1.33 (1.20–1.47)<br>P for trend < 0.001	1.30 (1.16–1.46)<br>P for trend < 0.001
CHG-related	CHG-RFM	Per SD	2.95 (2.58–3.37)<br>P < 0.001	2.98 (2.60–3.41)<br>P < 0.001	1.72 (1.47–2.03)<br>P < 0.001	1.78 (1.47–2.15)<br>P < 0.001
		Q1	1.00 (Ref)	1.00 (Ref)	1.00 (Ref)	1.00 (Ref)
		Q2	2.41 (1.79–3.23)<br>P < 0.001	2.47 (1.84–3.32)<br>P < 0.001	1.68 (1.24–2.29)<br>P < 0.001	1.67 (1.21–2.30)<br>P = 0.002
		Q3	5.04 (3.51–7.24)<br>P < 0.001	5.31 (3.68–7.66)<br>P < 0.001	2.32 (1.58–3.41)<br>P < 0.001	2.29 (1.51–3.46)<br>P < 0.001
		Q4	16.28 (10.86–24.41)<br>P < 0.001	17.32 (11.49–26.09)<br>P < 0.001	4.84 (3.07–7.63)<br>P < 0.001	4.66 (2.81–7.71)<br>P < 0.001
		Trend test	2.57 (2.25–2.93)<br>P for trend < 0.001	2.62 (2.29–3.00)<br>P for trend < 0.001	1.70 (1.46–1.97)<br>P for trend < 0.001	1.67 (1.42–1.97)<br>P for trend < 0.001
CHG-related	CHG-CVAI	Per SD	1.75 (1.62–1.89)<br>P < 0.001	1.76 (1.63–1.90)<br>P < 0.001	1.35 (1.23–1.48)<br>P < 0.001	1.41 (1.26–1.58)<br>P < 0.001
		Q1	1.00 (Ref)	1.00 (Ref)	1.00 (Ref)	1.00 (Ref)
		Q2	1.57 (1.12–2.21)<br>P = 0.009	1.58 (1.12–2.22)<br>P = 0.008	1.40 (1.00–1.98)<br>P = 0.052	1.37 (0.96–1.95)<br>P = 0.086
		Q3	2.99 (2.19–4.07)<br>P < 0.001	3.03 (2.22–4.14)<br>P < 0.001	2.26 (1.63–3.11)<br>P < 0.001	2.26 (1.59–3.22)<br>P < 0.001

Family	Marker	Scale / category	Model 1	Model 2	Model 3	Model 4
		Q4	4.89 (3.63–6.58)<br>P < 0.001	5.00 (3.71–6.73)<br>P < 0.001	2.69 (1.93–3.75)<br>P < 0.001	2.90 (1.98–4.25)<br>P < 0.001
		Trend test	1.72 (1.58–1.87)<br>P for trend < 0.001	1.73 (1.59–1.89)<br>P for trend < 0.001	1.38 (1.26–1.53)<br>P for trend < 0.001	1.44 (1.28–1.61)<br>P for trend < 0.001
**Adiposity/body-shape**						
Adiposity/body-shape	BMI	Per SD	1.49 (1.39–1.59)<br>P < 0.001	1.50 (1.40–1.61)<br>P < 0.001	1.30 (1.20–1.41)<br>P < 0.001	1.30 (1.20–1.42)<br>P < 0.001
		Q1	1.00 (Ref)	1.00 (Ref)	1.00 (Ref)	1.00 (Ref)
		Q2	1.59 (1.20–2.11)<br>P = 0.001	1.63 (1.23–2.16)<br>P < 0.001	1.35 (1.02–1.79)<br>P = 0.039	1.32 (0.99–1.75)<br>P = 0.057
		Q3	1.64 (1.24–2.17)<br>P < 0.001	1.69 (1.28–2.25)<br>P < 0.001	1.25 (0.94–1.67)<br>P = 0.128	1.20 (0.90–1.62)<br>P = 0.220
		Q4	3.34 (2.58–4.32)<br>P < 0.001	3.46 (2.67–4.49)<br>P < 0.001	2.12 (1.61–2.78)<br>P < 0.001	2.03 (1.53–2.71)<br>P < 0.001
		Trend test	1.47 (1.36–1.59)<br>P for trend < 0.001	1.49 (1.37–1.61)<br>P for trend < 0.001	1.27 (1.16–1.38)<br>P for trend < 0.001	1.25 (1.14–1.37)<br>P for trend < 0.001
Adiposity/body-shape	WC	Per SD	1.59 (1.47–1.73)<br>P < 0.001	1.61 (1.48–1.74)<br>P < 0.001	1.34 (1.23–1.46)<br>P < 0.001	1.33 (1.21–1.46)<br>P < 0.001
		Q1	1.00 (Ref)	1.00 (Ref)	1.00 (Ref)	1.00 (Ref)
		Q2	1.70 (1.27–2.29)<br>P < 0.001	1.71 (1.27–2.30)<br>P < 0.001	1.51 (1.13–2.03)<br>P = 0.006	1.47 (1.09–1.98)<br>P = 0.012
		Q3	1.89 (1.41–2.52)<br>P < 0.001	1.91 (1.43–2.55)<br>P < 0.001	1.43 (1.07–1.93)<br>P = 0.017	1.39 (1.02–1.88)<br>P = 0.035
		Q4	3.71 (2.85–4.83)<br>P < 0.001	3.78 (2.90–4.92)<br>P < 0.001	2.29 (1.73–3.02)<br>P < 0.001	2.20 (1.64–2.94)<br>P < 0.001
		Trend test	1.52 (1.41–1.65)<br>P for trend < 0.001	1.53 (1.41–1.66)<br>P for trend < 0.001	1.29 (1.18–1.40)<br>P for trend < 0.001	1.27 (1.17–1.39)<br>P for trend < 0.001
Adiposity/body-shape	WHtR	Per SD	1.61 (1.48–1.75)<br>P < 0.001	1.63 (1.50–1.78)<br>P < 0.001	1.34 (1.22–1.46)<br>P < 0.001	1.32 (1.20–1.45)<br>P < 0.001
		Q1	1.00 (Ref)	1.00 (Ref)	1.00 (Ref)	1.00 (Ref)
		Q2	1.67 (1.23–2.27)<br>P < 0.001	1.68 (1.24–2.28)<br>P < 0.001	1.46 (1.07–1.99)<br>P = 0.016	1.43 (1.05–1.95)<br>P = 0.024
		Q3	2.25 (1.67–3.02)<br>P < 0.001	2.29 (1.70–3.07)<br>P < 0.001	1.64 (1.22–2.22)<br>P = 0.001	1.60 (1.17–2.18)<br>P = 0.003
		Q4	3.99 (3.01–5.30)<br>P < 0.001	4.11 (3.09–5.47)<br>P < 0.001	2.44 (1.81–3.28)<br>P < 0.001	2.35 (1.73–3.21)<br>P < 0.001

Family	Marker	Scale / category	Model 1	Model 2	Model 3	Model 4
		Trend test	1.57 (1.44–1.71)<br>P for trend < 0.001	1.59 (1.46–1.73)<br>P for trend < 0.001	1.33 (1.21–1.45)<br>P for trend < 0.001	1.31 (1.20–1.44)<br>P for trend < 0.001
Adiposity/body-shape	BRI	Per SD	1.57 (1.45–1.70)<br>P < 0.001	1.59 (1.46–1.72)<br>P < 0.001	1.31 (1.20–1.43)<br>P < 0.001	1.29 (1.18–1.42)<br>P < 0.001
		Q1	1.00 (Ref)	1.00 (Ref)	1.00 (Ref)	1.00 (Ref)
		Q2	1.67 (1.23–2.27)<br>P < 0.001	1.68 (1.24–2.28)<br>P < 0.001	1.46 (1.07–1.99)<br>P = 0.016	1.43 (1.05–1.95)<br>P = 0.024
		Q3	2.25 (1.67–3.02)<br>P < 0.001	2.29 (1.70–3.07)<br>P < 0.001	1.64 (1.22–2.22)<br>P = 0.001	1.60 (1.17–2.18)<br>P = 0.003
		Q4	3.99 (3.01–5.30)<br>P < 0.001	4.11 (3.09–5.47)<br>P < 0.001	2.44 (1.81–3.28)<br>P < 0.001	2.35 (1.73–3.21)<br>P < 0.001
		Trend test	1.57 (1.44–1.71)<br>P for trend < 0.001	1.59 (1.46–1.73)<br>P for trend < 0.001	1.33 (1.21–1.45)<br>P for trend < 0.001	1.31 (1.20–1.44)<br>P for trend < 0.001
Adiposity/body-shape	ABSI	Per SD	1.14 (1.05–1.24)<br>P = 0.002	1.14 (1.05–1.24)<br>P = 0.002	1.05 (0.96–1.15)<br>P = 0.245	1.04 (0.95–1.14)<br>P = 0.353
		Q1	1.00 (Ref)	1.00 (Ref)	1.00 (Ref)	1.00 (Ref)
		Q2	1.50 (1.17–1.94)<br>P = 0.002	1.50 (1.17–1.94)<br>P = 0.002	1.39 (1.07–1.79)<br>P = 0.012	1.37 (1.06–1.77)<br>P = 0.016
		Q3	1.40 (1.08–1.82)<br>P = 0.010	1.41 (1.09–1.83)<br>P = 0.009	1.15 (0.88–1.49)<br>P = 0.301	1.10 (0.85–1.43)<br>P = 0.468
		Q4	1.60 (1.24–2.08)<br>P < 0.001	1.60 (1.24–2.08)<br>P < 0.001	1.26 (0.97–1.63)<br>P = 0.089	1.22 (0.94–1.59)<br>P = 0.141
		Trend test	1.13 (1.05–1.23)<br>P for trend = 0.002	1.13 (1.05–1.23)<br>P for trend = 0.002	1.04 (0.96–1.13)<br>P for trend = 0.316	1.03 (0.95–1.12)<br>P for trend = 0.470
Adiposity/body-shape	WWI	Per SD	1.34 (1.22–1.47)<br>P < 0.001	1.35 (1.23–1.48)<br>P < 0.001	1.16 (1.05–1.28)<br>P = 0.003	1.13 (1.03–1.25)<br>P = 0.011
		Q1	1.00 (Ref)	1.00 (Ref)	1.00 (Ref)	1.00 (Ref)
		Q2	1.75 (1.33–2.32)<br>P < 0.001	1.76 (1.33–2.33)<br>P < 0.001	1.46 (1.10–1.94)<br>P = 0.008	1.42 (1.07–1.88)<br>P = 0.015
		Q3	2.02 (1.53–2.66)<br>P < 0.001	2.04 (1.55–2.69)<br>P < 0.001	1.47 (1.11–1.95)<br>P = 0.007	1.41 (1.06–1.87)<br>P = 0.018
		Q4	2.35 (1.76–3.13)<br>P < 0.001	2.39 (1.79–3.20)<br>P < 0.001	1.55 (1.15–2.08)<br>P = 0.004	1.47 (1.09–1.98)<br>P = 0.011
		Trend test	1.28 (1.18–1.40)<br>P for trend < 0.001	1.29 (1.19–1.41)<br>P for trend < 0.001	1.12 (1.03–1.23)<br>P for trend = 0.011	1.10 (1.01–1.21)<br>P for trend = 0.032
Adiposity/body-shape	CI	Per SD	1.35 (1.24–1.47)<br>P < 0.001	1.35 (1.24–1.47)<br>P < 0.001	1.17 (1.07–1.28)<br>P < 0.001	1.15 (1.05–1.26)<br>P = 0.003

Family	Marker	Scale / category	Model 1	Model 2	Model 3	Model 4
		Q1	1.00 (Ref)	1.00 (Ref)	1.00 (Ref)	1.00 (Ref)
		Q2	1.50 (1.14–1.99)<br>P = 0.004	1.51 (1.14–2.00)<br>P = 0.004	1.35 (1.02–1.79)<br>P = 0.036	1.30 (0.98–1.72)<br>P = 0.069
		Q3	1.77 (1.35–2.32)<br>P < 0.001	1.79 (1.36–2.35)<br>P < 0.001	1.30 (0.99–1.72)<br>P = 0.059	1.24 (0.94–1.63)<br>P = 0.137
		Q4	2.58 (1.98–3.35)<br>P < 0.001	2.59 (1.99–3.37)<br>P < 0.001	1.73 (1.32–2.26)<br>P < 0.001	1.64 (1.24–2.15)<br>P < 0.001
		Trend test	1.35 (1.24–1.46)<br>P for trend < 0.001	1.35 (1.25–1.46)<br>P for trend < 0.001	1.17 (1.08–1.28)<br>P for trend < 0.001	1.16 (1.06–1.26)<br>P for trend < 0.001
Adiposity/body-shape	RFM	Per SD	2.75 (2.28–3.31)<br>P < 0.001	2.82 (2.34–3.41)<br>P < 0.001	1.85 (1.52–2.25)<br>P < 0.001	1.79 (1.46–2.20)<br>P < 0.001
		Q1	1.00 (Ref)	1.00 (Ref)	1.00 (Ref)	1.00 (Ref)
		Q2	1.98 (1.52–2.59)<br>P < 0.001	2.01 (1.54–2.63)<br>P < 0.001	1.36 (1.03–1.80)<br>P = 0.028	1.30 (0.98–1.72)<br>P = 0.074
		Q3	3.39 (1.98–5.80)<br>P < 0.001	3.59 (2.09–6.19)<br>P < 0.001	1.89 (1.11–3.22)<br>P = 0.018	1.69 (0.98–2.89)<br>P = 0.057
		Q4	6.93 (3.98–12.07)<br>P < 0.001	7.38 (4.21–12.95)<br>P < 0.001	2.83 (1.62–4.94)<br>P < 0.001	2.45 (1.38–4.33)<br>P = 0.002
		Trend test	1.97 (1.69–2.30)<br>P for trend < 0.001	2.00 (1.71–2.33)<br>P for trend < 0.001	1.43 (1.22–1.68)<br>P for trend < 0.001	1.37 (1.16–1.62)<br>P for trend < 0.001
Adiposity/body-shape	CVAI	Per SD	1.69 (1.56–1.84)<br>P < 0.001	1.71 (1.57–1.85)<br>P < 0.001	1.36 (1.24–1.50)<br>P < 0.001	1.40 (1.26–1.57)<br>P < 0.001
		Q1	1.00 (Ref)	1.00 (Ref)	1.00 (Ref)	1.00 (Ref)
		Q2	1.89 (1.37–2.62)<br>P < 0.001	1.91 (1.38–2.65)<br>P < 0.001	1.70 (1.23–2.36)<br>P = 0.001	1.64 (1.17–2.29)<br>P = 0.004
		Q3	2.59 (1.90–3.55)<br>P < 0.001	2.64 (1.93–3.61)<br>P < 0.001	1.94 (1.40–2.68)<br>P < 0.001	1.92 (1.36–2.70)<br>P < 0.001
		Q4	4.73 (3.52–6.36)<br>P < 0.001	4.83 (3.59–6.50)<br>P < 0.001	2.78 (2.01–3.84)<br>P < 0.001	2.90 (2.03–4.14)<br>P < 0.001
		Trend test	1.64 (1.51–1.79)<br>P for trend < 0.001	1.65 (1.52–1.80)<br>P for trend < 0.001	1.36 (1.24–1.49)<br>P for trend < 0.001	1.39 (1.25–1.54)<br>P for trend < 0.001
**TyG-related**						
TyG-related	TyG	Per SD	1.66 (1.54–1.78)<br>P < 0.001	1.65 (1.54–1.78)<br>P < 0.001	1.11 (1.01–1.23)<br>P = 0.033	1.14 (1.02–1.27)<br>P = 0.022
		Q1	1.00 (Ref)	1.00 (Ref)	1.00 (Ref)	1.00 (Ref)

Family	Marker	Scale / category	Model 1	Model 2	Model 3	Model 4
		Q2	1.65 (1.23–2.23)<br>P < 0.001	1.64 (1.21–2.21)<br>P = 0.001	1.39 (1.03–1.88)<br>P = 0.030	1.30 (0.96–1.77)<br>P = 0.093
		Q3	2.02 (1.52–2.70)<br>P < 0.001	2.02 (1.51–2.69)<br>P < 0.001	1.33 (0.98–1.80)<br>P = 0.063	1.18 (0.86–1.61)<br>P = 0.310
		Q4	3.73 (2.85–4.88)<br>P < 0.001	3.72 (2.84–4.87)<br>P < 0.001	1.39 (1.00–1.92)<br>P = 0.050	1.28 (0.91–1.80)<br>P = 0.156
		Trend test	1.53 (1.42–1.66)<br>P for trend < 0.001	1.53 (1.42–1.66)<br>P for trend < 0.001	1.08 (0.98–1.20)<br>P for trend = 0.121	1.06 (0.95–1.17)<br>P for trend = 0.317
TyG-related	TyG-BMI	Per SD	1.67 (1.56–1.79)<br>P < 0.001	1.69 (1.57–1.81)<br>P < 0.001	1.32 (1.21–1.44)<br>P < 0.001	1.36 (1.23–1.49)<br>P < 0.001
		Q1	1.00 (Ref)	1.00 (Ref)	1.00 (Ref)	1.00 (Ref)
		Q2	1.52 (1.11–2.07)<br>P = 0.008	1.55 (1.13–2.11)<br>P = 0.006	1.33 (0.97–1.82)<br>P = 0.074	1.29 (0.94–1.77)<br>P = 0.119
		Q3	2.22 (1.65–2.97)<br>P < 0.001	2.28 (1.70–3.06)<br>P < 0.001	1.59 (1.17–2.16)<br>P = 0.003	1.56 (1.13–2.14)<br>P = 0.007
		Q4	4.53 (3.45–5.94)<br>P < 0.001	4.70 (3.58–6.19)<br>P < 0.001	2.39 (1.76–3.24)<br>P < 0.001	2.42 (1.74–3.37)<br>P < 0.001
		Trend test	1.68 (1.55–1.83)<br>P for trend < 0.001	1.70 (1.57–1.85)<br>P for trend < 0.001	1.34 (1.22–1.47)<br>P for trend < 0.001	1.35 (1.22–1.50)<br>P for trend < 0.001
TyG-related	TyG-WC	Per SD	1.78 (1.65–1.91)<br>P < 0.001	1.79 (1.66–1.93)<br>P < 0.001	1.34 (1.22–1.46)<br>P < 0.001	1.37 (1.24–1.52)<br>P < 0.001
		Q1	1.00 (Ref)	1.00 (Ref)	1.00 (Ref)	1.00 (Ref)
		Q2	1.83 (1.32–2.53)<br>P < 0.001	1.83 (1.32–2.54)<br>P < 0.001	1.59 (1.15–2.22)<br>P = 0.005	1.51 (1.08–2.12)<br>P = 0.016
		Q3	2.70 (1.98–3.67)<br>P < 0.001	2.73 (2.01–3.72)<br>P < 0.001	1.97 (1.43–2.71)<br>P < 0.001	1.87 (1.34–2.62)<br>P < 0.001
		Q4	5.03 (3.77–6.71)<br>P < 0.001	5.11 (3.82–6.83)<br>P < 0.001	2.56 (1.85–3.54)<br>P < 0.001	2.55 (1.80–3.62)<br>P < 0.001
		Trend test	1.70 (1.56–1.84)<br>P for trend < 0.001	1.71 (1.57–1.85)<br>P for trend < 0.001	1.33 (1.21–1.47)<br>P for trend < 0.001	1.34 (1.21–1.49)<br>P for trend < 0.001
TyG-related	TyG-WHtR	Per SD	1.81 (1.67–1.95)<br>P < 0.001	1.83 (1.69–1.98)<br>P < 0.001	1.33 (1.21–1.47)<br>P < 0.001	1.36 (1.22–1.51)<br>P < 0.001
		Q1	1.00 (Ref)	1.00 (Ref)	1.00 (Ref)	1.00 (Ref)
		Q2	1.91 (1.37–2.66)<br>P < 0.001	1.93 (1.39–2.68)<br>P < 0.001	1.65 (1.18–2.30)<br>P = 0.003	1.62 (1.15–2.29)<br>P = 0.005
		Q3	2.80 (2.05–3.83)<br>P < 0.001	2.85 (2.08–3.89)<br>P < 0.001	1.94 (1.41–2.69)<br>P < 0.001	1.86 (1.32–2.62)<br>P < 0.001

Family	Marker	Scale / category	Model 1	Model 2	Model 3	Model 4
		Q4	5.25 (3.89–7.08)<br>P < 0.001	5.38 (3.98–7.27)<br>P < 0.001	2.54 (1.82–3.54)<br>P < 0.001	2.52 (1.76–3.61)<br>P < 0.001
		Trend test	1.71 (1.57–1.86)<br>P for trend < 0.001	1.72 (1.58–1.88)<br>P for trend < 0.001	1.32 (1.20–1.45)<br>P for trend < 0.001	1.32 (1.19–1.46)<br>P for trend < 0.001
TyG-related	TyG-BRI	Per SD	1.68 (1.55–1.81)<br>P < 0.001	1.70 (1.57–1.84)<br>P < 0.001	1.31 (1.20–1.43)<br>P < 0.001	1.31 (1.19–1.43)<br>P < 0.001
		Q1	1.00 (Ref)	1.00 (Ref)	1.00 (Ref)	1.00 (Ref)
		Q2	1.73 (1.26–2.37)<br>P < 0.001	1.74 (1.27–2.39)<br>P < 0.001	1.48 (1.07–2.04)<br>P = 0.017	1.42 (1.03–1.97)<br>P = 0.033
		Q3	2.39 (1.76–3.23)<br>P < 0.001	2.43 (1.79–3.29)<br>P < 0.001	1.68 (1.23–2.30)<br>P = 0.001	1.60 (1.16–2.22)<br>P = 0.004
		Q4	4.73 (3.54–6.32)<br>P < 0.001	4.89 (3.65–6.54)<br>P < 0.001	2.53 (1.85–3.46)<br>P < 0.001	2.45 (1.76–3.41)<br>P < 0.001
		Trend test	1.67 (1.54–1.82)<br>P for trend < 0.001	1.69 (1.55–1.85)<br>P for trend < 0.001	1.34 (1.22–1.48)<br>P for trend < 0.001	1.34 (1.21–1.48)<br>P for trend < 0.001
TyG-related	TyG-ABSI	Per SD	1.58 (1.46–1.70)<br>P < 0.001	1.57 (1.46–1.69)<br>P < 0.001	1.10 (1.01–1.21)<br>P = 0.036	1.10 (1.00–1.22)<br>P = 0.046
		Q1	1.00 (Ref)	1.00 (Ref)	1.00 (Ref)	1.00 (Ref)
		Q2	1.72 (1.26–2.35)<br>P < 0.001	1.72 (1.26–2.34)<br>P < 0.001	1.44 (1.05–1.96)<br>P = 0.024	1.36 (0.99–1.87)<br>P = 0.060
		Q3	2.50 (1.86–3.35)<br>P < 0.001	2.50 (1.86–3.36)<br>P < 0.001	1.66 (1.22–2.25)<br>P = 0.001	1.51 (1.10–2.07)<br>P = 0.010
		Q4	3.80 (2.86–5.05)<br>P < 0.001	3.77 (2.84–5.01)<br>P < 0.001	1.56 (1.13–2.15)<br>P = 0.007	1.47 (1.05–2.05)<br>P = 0.023
		Trend test	1.53 (1.41–1.66)<br>P for trend < 0.001	1.53 (1.41–1.66)<br>P for trend < 0.001	1.13 (1.02–1.24)<br>P for trend = 0.015	1.11 (1.00–1.22)<br>P for trend = 0.043
TyG-related	TyG-WWI	Per SD	1.71 (1.58–1.85)<br>P < 0.001	1.71 (1.58–1.85)<br>P < 0.001	1.18 (1.07–1.31)<br>P < 0.001	1.19 (1.07–1.32)<br>P = 0.001
		Q1	1.00 (Ref)	1.00 (Ref)	1.00 (Ref)	1.00 (Ref)
		Q2	1.85 (1.34–2.56)<br>P < 0.001	1.86 (1.35–2.57)<br>P < 0.001	1.47 (1.06–2.04)<br>P = 0.020	1.43 (1.02–1.99)<br>P = 0.038
		Q3	2.92 (2.15–3.97)<br>P < 0.001	2.95 (2.17–4.00)<br>P < 0.001	1.85 (1.35–2.55)<br>P < 0.001	1.72 (1.23–2.39)<br>P = 0.001
		Q4	4.55 (3.37–6.14)<br>P < 0.001	4.59 (3.40–6.20)<br>P < 0.001	1.84 (1.31–2.58)<br>P < 0.001	1.73 (1.21–2.47)<br>P = 0.002
		Trend test	1.62 (1.49–1.77)<br>P for trend < 0.001	1.63 (1.49–1.77)<br>P for trend < 0.001	1.19 (1.08–1.31)<br>P for trend < 0.001	1.17 (1.05–1.29)<br>P for trend = 0.004

Family	Marker	Scale / category	Model 1	Model 2	Model 3	Model 4
TyG-related	TyG-CI	Per SD	1.69 (1.57–1.82)<br>P < 0.001	1.69 (1.57–1.82)<br>P < 0.001	1.19 (1.08–1.31)<br>P < 0.001	1.20 (1.08–1.33)<br>P < 0.001
		Q1	1.00 (Ref)	1.00 (Ref)	1.00 (Ref)	1.00 (Ref)
		Q2	1.97 (1.41–2.74)<br>P < 0.001	1.96 (1.41–2.73)<br>P < 0.001	1.63 (1.16–2.27)<br>P = 0.004	1.55 (1.10–2.18)<br>P = 0.013
		Q3	3.07 (2.25–4.20)<br>P < 0.001	3.09 (2.26–4.22)<br>P < 0.001	2.08 (1.50–2.87)<br>P < 0.001	1.92 (1.37–2.69)<br>P < 0.001
		Q4	4.82 (3.57–6.52)<br>P < 0.001	4.81 (3.56–6.50)<br>P < 0.001	2.07 (1.47–2.91)<br>P < 0.001	1.96 (1.37–2.81)<br>P < 0.001
		Trend test	1.64 (1.51–1.78)<br>P for trend < 0.001	1.64 (1.51–1.78)<br>P for trend < 0.001	1.22 (1.11–1.35)<br>P for trend < 0.001	1.20 (1.09–1.33)<br>P for trend < 0.001
TyG-related	TyG-RFM	Per SD	2.86 (2.48–3.29)<br>P < 0.001	2.90 (2.52–3.34)<br>P < 0.001	1.67 (1.41–1.98)<br>P < 0.001	1.73 (1.44–2.08)<br>P < 0.001
		Q1	1.00 (Ref)	1.00 (Ref)	1.00 (Ref)	1.00 (Ref)
		Q2	2.13 (1.60–2.83)<br>P < 0.001	2.17 (1.63–2.88)<br>P < 0.001	1.47 (1.09–1.99)<br>P = 0.011	1.47 (1.08–2.00)<br>P = 0.014
		Q3	4.70 (3.25–6.79)<br>P < 0.001	5.00 (3.44–7.25)<br>P < 0.001	2.15 (1.46–3.18)<br>P < 0.001	2.18 (1.45–3.29)<br>P < 0.001
		Q4	14.59 (9.60–22.18)<br>P < 0.001	15.65 (10.25–23.89)<br>P < 0.001	4.46 (2.80–7.12)<br>P < 0.001	4.45 (2.70–7.34)<br>P < 0.001
		Trend test	2.48 (2.16–2.85)<br>P for trend < 0.001	2.54 (2.21–2.92)<br>P for trend < 0.001	1.67 (1.43–1.94)<br>P for trend < 0.001	1.67 (1.41–1.97)<br>P for trend < 0.001
TyG-related	TyG-CVAI	Per SD	1.72 (1.59–1.85)<br>P < 0.001	1.73 (1.60–1.87)<br>P < 0.001	1.33 (1.21–1.46)<br>P < 0.001	1.39 (1.25–1.55)<br>P < 0.001
		Q1	1.00 (Ref)	1.00 (Ref)	1.00 (Ref)	1.00 (Ref)
		Q2	1.72 (1.23–2.41)<br>P = 0.002	1.73 (1.23–2.42)<br>P = 0.001	1.55 (1.11–2.18)<br>P = 0.011	1.49 (1.05–2.12)<br>P = 0.026
		Q3	3.08 (2.25–4.21)<br>P < 0.001	3.12 (2.28–4.26)<br>P < 0.001	2.30 (1.66–3.18)<br>P < 0.001	2.24 (1.58–3.18)<br>P < 0.001
		Q4	4.80 (3.56–6.48)<br>P < 0.001	4.90 (3.62–6.62)<br>P < 0.001	2.63 (1.89–3.68)<br>P < 0.001	2.75 (1.90–3.99)<br>P < 0.001
		Trend test	1.68 (1.54–1.82)<br>P for trend < 0.001	1.69 (1.55–1.84)<br>P for trend < 0.001	1.35 (1.22–1.49)<br>P for trend < 0.001	1.38 (1.24–1.54)<br>P for trend < 0.001

Note. This supplementary table presents the sequential Cox regression results for all 29 candidate indices. Each index was entered into the Cox model separately. Per-SD estimates indicate the HR associated with a 1-SD increase in the corresponding index. Quartile analyses used Q1 as the reference group, and P for trend was calculated by modeling quartiles as an ordinal variable. Model 1 adjusted for age and sex. Model 2 further adjusted for education level, marital status, Hukou, smoking status, and drinking status. Model 3 further adjusted for hypertension, diabetes, dyslipidemia, and kidney disease and was used to anchor the primary association interpretation. Model 4 additionally adjusted for baseline CKM stage, SBP, DBP, HDL-C, LDL-C, eGFR, uric acid, BUN, hemoglobin, MCV, PCV, platelet count, and white blood cell count and was retained as an extended clinical-adjustment analysis. Model 4 estimates were interpreted as conditional associations after broader adjustment rather than as definitive independent or causal effects.

Abbreviations: HR, hazard ratio; CI, confidence interval; CMM, cardiometabolic multimorbidity; CKM, cardiovascular–kidney–metabolic.

Supplementary Table S3C. CKM stage-stratified Cox associations

Family	Marker	CKM stage 0 HR (95% CI)	CKM stage 1 HR (95% CI)	CKM stage 2 HR (95% CI)	CKM stage 3 HR (95% CI)	P for multiplicative interaction term
CHG-related	CHG-RFM	1.14 (0.21–6.24)	2.27 (0.94–5.45)	2.66 (2.09–3.39)	1.92 (1.54–2.39)	Stage 1: P = 0.759<br>Stage 2: P = 0.125<br>Stage 3: P = 0.233
Adiposity/body-shape	RFM	1.40 (0.28–7.07)	1.90 (0.82–4.38)	2.46 (1.82–3.32)	1.74 (1.31–2.31)	Stage 1: P = 0.901<br>Stage 2: P = 0.087<br>Stage 3: P = 0.132
TyG-related	TyG-RFM	2.16 (0.38–12.41)	2.18 (0.90–5.27)	2.40 (1.89–3.06)	1.85 (1.48–2.33)	Stage 1: P = 0.980<br>Stage 2: P = 0.232<br>Stage 3: P = 0.354
CHG-related	CHG-BMI	0.61 (0.18–2.07)	1.56 (0.92–2.64)	1.71 (1.52–1.93)	1.34 (1.19–1.50)	Stage 1: P = 0.236<br>Stage 2: P = 0.064<br>Stage 3: P = 0.111
CHG-related	CHG-WC	1.08 (0.31–3.78)	1.59 (0.94–2.71)	1.79 (1.56–2.06)	1.43 (1.28–1.61)	Stage 1: P = 0.714<br>Stage 2: P = 0.455<br>Stage 3: P = 0.646
CHG-related	CHG-WHtR	1.34 (0.39–4.56)	1.64 (0.95–2.82)	1.79 (1.55–2.06)	1.46 (1.29–1.65)	Stage 1: P = 0.674<br>Stage 2: P = 0.347<br>Stage 3: P = 0.501
CHG-related	CHG-CVAI	1.27 (0.39–4.12)	1.56 (0.94–2.60)	1.76 (1.52–2.04)	1.32 (1.18–1.48)	Stage 1: P = 0.745<br>Stage 2: P = 0.645<br>Stage 3: P = 0.965
Adiposity/body-shape	CVAI	1.26 (0.46–3.43)	1.44 (0.92–2.27)	1.67 (1.44–1.94)	1.27 (1.13–1.44)	Stage 1: P = 0.873<br>Stage 2: P = 0.713<br>Stage 3: P = 0.830
TyG-related	TyG-BMI	0.89 (0.26–3.12)	1.50 (0.90–2.53)	1.62 (1.44–1.83)	1.31 (1.17–1.47)	Stage 1: P = 0.639<br>Stage 2: P = 0.300<br>Stage 3: P = 0.407
TyG-related	TyG-WC	1.83 (0.52–6.39)	1.55 (0.92–2.61)	1.66 (1.45–1.90)	1.39 (1.24–1.57)	Stage 1: P = 0.530<br>Stage 2: P = 0.689<br>Stage 3: P = 0.542
TyG-related	TyG-CVAI	1.42 (0.45–4.48)	1.52 (0.92–2.53)	1.68 (1.45–1.94)	1.30 (1.16–1.46)	Stage 1: P = 0.934<br>Stage 2: P = 0.865<br>Stage 3: P = 0.771

Note. CKM stage-stratified Cox models and multiplicative interaction tests were conducted as separate secondary analyses. HRs indicate the associations of each 1-SD increment in the corresponding index with incident CMM within baseline CKM stages 0, 1, 2, and 3. These analyses were not treated as an additional step in the sequential adjustment hierarchy. Interaction estimates were used to explore potential heterogeneity across CKM stages. Estimates from strata with sparse events, particularly CKM stage 0, should be interpreted cautiously. Abbreviations: HR, hazard ratio; CI, confidence interval; CKM, cardiovascular–kidney–metabolic; CMM, cardiometabolic multimorbidity.

Supplementary Table S4. Multicollinearity diagnostics for the index-specific Cox models

Panel A. Model-level summary

Model	Complete-case N/events	Maximum adjusted GVIF	Term producing maximum	Index models with moderate values	Adjusted GVIF ≥5 / errors
Model 1	7,018/569	2.195	RFM in the RFM model	RFM	0/0
Model 2	7,014/569	2.436	Sex in the RFM model	CHG-RFM, RFM, TyG-RFM	0/0
Model 3	7,014/569	2.474	Sex in the RFM model	CHG-RFM, RFM, TyG-RFM	0/0
Model 4	7,013/568	2.704	Sex in the RFM model	CHG-RFM, RFM, TyG-RFM	0/0

Panel B. Moderate GVIF findings in Model 4

Index-specific model	Adjusted GVIF for index	Adjusted GVIF for sex	Maximum adjusted GVIF
CHG-RFM	2.439	2.552	2.552
RFM	2.425	2.704	2.704
TyG-RFM	2.336	2.557	2.557
Other 26 indices	1.104–1.947	—	≤1.947

S4. Added value of all candidate indices for long-term CMM risk identification

For clarity of presentation, the main prediction results highlighted representative indices that showed more consistent added risk information across complementary metrics. AUC, ΔAUC, and IDI at the 8-year prediction horizon are presented in Supplementary Table S4A; cNRI, survival NRI, and landmark internal-validation metrics are presented in Supplementary Table S4B; and exploratory optimal cut-off values with corresponding classification performance are presented in Supplementary Table S4C. The added predictive ability of each candidate index was evaluated using complementary measures of discrimination and reclassification in accordance with established principles for evaluating new risk markers [4]. Time-dependent AUCs and their comparisons at the 8-year prediction horizon were estimated using methods for censored time-to-event outcomes [8].

Supplementary Tables S4A and S4B summarize the added value of all 29 candidate indices beyond the literature-style extended clinical baseline model. Supplementary Table S4C provides exploratory optimal cut-off values and classification performance for representative indices at the 8-year prediction horizon. To avoid excessive table width, AUC/IDI metrics, reclassification/internal-validation metrics, and threshold-based classification metrics are presented separately.

Supplementary Table S4A. AUC and IDI at 8 years

Index	Family	AUC	ΔAUC	P for ΔAUC	IDI (95% CI)	P for IDI
CHG-BMI	CHG-related	0.800	0.0129	<0.001	0.0111 (0.0042 to 0.0200)	<0.001
CHG-WC	CHG-related	0.800	0.0126	<0.001	0.0113 (0.0048 to 0.0203)	<0.001
WC	Adiposity/body-shape	0.799	0.0121	0.002	0.0115 (0.0052 to 0.0214)	<0.001
CHG-BRI	CHG-related	0.799	0.0119	<0.001	0.0088 (0.0033 to 0.0165)	<0.001
CHG-WHtR	CHG-related	0.799	0.0119	<0.001	0.0087 (0.0030 to 0.0163)	<0.001
TyG-BMI	TyG-related	0.799	0.0117	<0.001	0.0110 (0.0041 to 0.0199)	<0.001
BMI	Adiposity/body-shape	0.799	0.0117	<0.001	0.0103 (0.0037 to 0.0195)	<0.001
BRI	Adiposity/body-shape	0.798	0.0114	<0.001	0.0084 (0.0035 to 0.0160)	<0.001
TyG-BRI	TyG-related	0.798	0.0114	<0.001	0.0086 (0.0033 to 0.0162)	<0.001
WHtR	Adiposity/body-shape	0.798	0.0113	0.002	0.0087 (0.0037 to 0.0162)	<0.001
CHG-RFM	CHG-related	0.798	0.0113	0.002	0.0092 (0.0036 to 0.0163)	<0.001
CHG-CVAI	CHG-related	0.798	0.0111	0.003	0.0112 (0.0048 to 0.0203)	<0.001
TyG-WC	TyG-related	0.798	0.0111	0.002	0.0107 (0.0044 to 0.0195)	<0.001
CVAI	Adiposity/body-shape	0.798	0.0108	0.006	0.0115 (0.0052 to 0.0206)	<0.001
RFM	Adiposity/body-shape	0.798	0.0107	0.004	0.0087 (0.0041 to 0.0156)	<0.001
TyG-WHtR	TyG-related	0.798	0.0106	0.002	0.0083 (0.0028 to 0.0152)	<0.001
TyG-CVAI	TyG-related	0.797	0.0102	0.005	0.0108 (0.0045 to 0.0201)	<0.001
TyG-RFM	TyG-related	0.797	0.0101	0.004	0.0087 (0.0037 to 0.0157)	<0.001
CHG-CI	CHG-related	0.791	0.0043	0.043	0.0032 (0.0003 to 0.0081)	0.020
CHG-WWI	CHG-related	0.791	0.0035	0.062	0.0022 (-0.0002 to 0.0061)	0.068

Index	Family	AUC	ΔAUC	P for ΔAUC	IDI (95% CI)	P for IDI
CI	Adiposity/body-shape	0.790	0.0034	0.091	0.0027 (0.0007 to 0.0065)	0.008
TyG-CI	TyG-related	0.790	0.0031	0.131	0.0028 (0.0004 to 0.0067)	0.028
TyG-WWI	TyG-related	0.790	0.0026	0.158	0.0020 (0.0000 to 0.0051)	0.044
WWI	Adiposity/body-shape	0.790	0.0026	0.121	0.0016 (0.0002 to 0.0044)	0.024
CHG-ABSI	CHG-related	0.788	0.0011	0.317	0.0008 (-0.0003 to 0.0036)	0.148
CHG	CHG-related	0.788	0.0010	0.348	0.0008 (-0.0009 to 0.0053)	0.419
TyG-ABSI	TyG-related	0.788	0.0006	0.588	0.0008 (-0.0003 to 0.0029)	0.156
ABSI	Adiposity/body-shape	0.787	0.0005	0.441	0.0003 (-0.0003 to 0.0023)	0.184
TyG	TyG-related	0.787	-0.0001	0.949	0.0008 (-0.0008 to 0.0048)	0.263

Note. Analyses included 7,013 participants with complete information for the 8-year time-dependent prediction analysis, among whom 568 developed incident CMM within 8 years. This prediction cohort was defined separately from the descriptive and Model 3 complete-case cohorts according to the availability of the prediction-model variables and 8-year outcome information. Time-dependent AUCs were estimated at the 8-year prediction horizon using marginal inverse-probability-of-censoring weighting. ΔAUC was calculated as the AUC of the index-extended model minus that of the clinical baseline model. P values for ΔAUC were obtained using the iid-representation-based comparison of correlated time-dependent AUCs [8]. The AUC of the clinical baseline model was 0.787. Each candidate index was added separately to the baseline model. The table is sorted by ΔAUC in descending order. Abbreviations: AUC, area under the curve; ROC, receiver operating characteristic; IDI, integrated discrimination improvement; CMM, cardiometabolic multimorbidity; CI, confidence interval.

Supplementary Table S4B. NRI and internal-validation metrics

Index	Family	cNRI (95% CI)	P for cNRI	survival NRI (95% CI)	Corrected C-statistic	Corrected calibration slope
CHG-BMI	CHG-related	0.1120 (0.0669 to 0.1697)	<0.001	0.2003 (0.1025 to 0.2909)	0.725	0.921
CHG-WC	CHG-related	0.1478 (0.0785 to 0.1896)	<0.001	0.2362 (0.1017 to 0.3238)	0.726	0.920
WC	Adiposity/body-shape	0.1413 (0.0729 to 0.1882)	<0.001	0.2195 (0.1081 to 0.3144)	0.724	0.920
CHG-BRI	CHG-related	0.1127 (0.0465 to 0.1598)	<0.001	0.1877 (0.0693 to 0.2711)	0.725	0.920
CHG-WHtR	CHG-related	0.1159 (0.0469 to 0.1624)	<0.001	0.1949 (0.0822 to 0.2878)	0.726	0.920
TyG-BMI	TyG-related	0.1442 (0.0755 to 0.1855)	<0.001	0.2435 (0.1312 to 0.3191)	0.723	0.918
BMI	Adiposity/body-shape	0.1226 (0.0619 to 0.1687)	<0.001	0.1824 (0.0703 to 0.2588)	0.722	0.919
BRI	Adiposity/body-shape	0.1173 (0.0536 to 0.1682)	<0.001	0.1916 (0.0767 to 0.2799)	0.723	0.919
TyG-BRI	TyG-related	0.1040 (0.0514 to 0.1548)	<0.001	0.1818 (0.0824 to 0.2675)	0.724	0.919
WHtR	Adiposity/body-shape	0.1152 (0.0598 to 0.1745)	<0.001	0.1975 (0.0892 to 0.2867)	0.723	0.918
CHG-RFM	CHG-related	0.1359 (0.0648 to 0.1784)	<0.001	0.2410 (0.1177 to 0.3202)	0.726	0.920
CHG-CVAI	CHG-related	0.1226 (0.0577 to 0.1729)	<0.001	0.2099 (0.0998 to 0.2950)	0.724	0.919
TyG-WC	TyG-related	0.1515 (0.0817 to 0.1996)	<0.001	0.2588 (0.1328 to 0.3347)	0.725	0.920
CVAI	Adiposity/body-shape	0.1289 (0.0623 to 0.1838)	<0.001	0.2202 (0.1103 to 0.3023)	0.723	0.919
RFM	Adiposity/body-shape	0.1207 (0.0594 to 0.1739)	<0.001	0.2128 (0.0947 to 0.2968)	0.723	0.917
TyG-WHtR	TyG-related	0.1285 (0.0668 to 0.1769)	<0.001	0.2312 (0.1262 to 0.3206)	0.724	0.919
TyG-CVAI	TyG-related	0.1229 (0.0612 to 0.1797)	<0.001	0.2082 (0.0994 to 0.2906)	0.723	0.917
TyG-RFM	TyG-related	0.1480 (0.0723 to 0.1927)	<0.001	0.2460 (0.1328 to 0.3276)	0.724	0.918
CHG-CI	CHG-related	0.0641 (-0.0093 to 0.1127)	0.084	0.1478 (0.0551 to 0.2418)	0.720	0.913
CHG-WWI	CHG-related	0.0389 (-0.0335 to 0.0900)	0.248	0.1006 (-0.0027 to 0.1989)	0.721	0.918
CI	Adiposity/body-shape	0.0708 (0.0024 to 0.1208)	0.044	0.1094 (0.0228 to 0.1945)	0.718	0.915
TyG-CI	TyG-related	0.0641 (-0.0080 to 0.1104)	0.084	0.1393 (0.0330 to 0.2371)	0.719	0.916
TyG-WWI	TyG-related	0.0410 (-0.0316 to 0.0904)	0.200	0.0949 (-0.0087 to 0.1988)	0.720	0.919
WWI	Adiposity/body-shape	0.0535 (-0.0150 to 0.1012)	0.120	0.0894 (-0.0050 to 0.1726)	0.717	0.914
CHG-ABSI	CHG-related	0.0283 (-0.0605 to 0.0842)	0.431	0.0959 (-0.0172 to 0.1778)	0.718	0.915
CHG	CHG-related	0.0229 (-0.0448 to 0.0767)	0.487	0.0732 (-0.0288 to 0.1617)	0.718	0.914
TyG-ABSI	TyG-related	-0.0037 (-0.0642 to 0.0540)	0.766	0.0349 (-0.0417 to 0.1459)	0.717	0.917
ABSI	Adiposity/body-shape	0.0130 (-0.0545 to 0.0514)	0.750	0.0243 (-0.0485 to 0.1055)	0.715	0.915

Index	Family	cNRI (95% CI)	P for cNRI	survival NRI (95% CI)	Corrected C-statistic	Corrected calibration slope
TyG	TyG-related	0.0291 (-0.0317 to 0.0796)	0.315	0.1008 (0.0015 to 0.1847)	0.717	0.918

Note. cNRI and survival NRI were used to compare each index-extended model with the clinical baseline model. Corrected C-statistics and corrected calibration slopes were obtained using 2015 landmark-based bootstrap internal validation. Survival NRI was considered statistically supported when its 95% CI was entirely above 0. These reclassification measures were interpreted alongside AUC, IDI, internal validation, calibration, and clinical interpretability. Abbreviations: cNRI, continuous net reclassification improvement; NRI, net reclassification improvement; CMM, cardiometabolic multimorbidity; CI, confidence interval.

Supplementary Table S4C. Exploratory cut-offs for 8-year CMM

Family	Index	N	Events within 8 years	Event rate, %	Optimal cut-off	Sensitivity	Specificity	PPV	NPV	Youden index
CHG-related	CHG-BMI	5935	569	9.6	131.1842	0.605	0.670	0.163	0.941	0.275
CHG-related	CHG-WC	5935	569	9.6	467.0881	0.649	0.647	0.163	0.946	0.295
Adiposity/body-shape	WC	5935	569	9.6	89.15	0.515	0.703	0.155	0.932	0.218
CHG-related	CHG-BRI	5935	569	9.6	24.5394	0.583	0.669	0.158	0.938	0.253
CHG-related	CHG-WHtR	5935	569	9.6	2.9680	0.654	0.642	0.162	0.946	0.296
TyG-related	TyG-BMI	5935	569	9.6	210.0050	0.615	0.644	0.155	0.940	0.259
TyG-related	TyG-WC	5935	569	9.6	771.9097	0.599	0.678	0.165	0.941	0.277
Adiposity/body-shape	CVAI	5935	569	9.6	94.8279	0.687	0.568	0.144	0.945	0.256
CHG-related	CHG-RFM	5935	569	9.6	213.1649	0.464	0.761	0.171	0.931	0.225

Note. Optimal cut-off values were derived from exploratory fixed-time binary ROC analyses for 8-year incident CMM. This fixed-time classification cohort included 5,935 participants and 569 events. It differed from the 7,013-participant time-dependent prediction cohort because participants without CMM who were censored before 8 years were excluded and because the fixed-time threshold analysis and the time-dependent prediction analysis applied different analytic eligibility requirements. Participants who developed CMM within 8 years were classified as events, and participants followed for at least 8 years without CMM were classified as non-events. For WC, the underlying threshold of 0.8915 m was converted to 89.15 cm for consistency with the unit used in Supplementary Table S2C; this unit conversion does not alter sensitivity, specificity, PPV, NPV, or the Youden index. Sensitivity, specificity, positive predictive value (PPV), negative predictive value (NPV), and Youden index were calculated at the selected threshold. The Youden index was calculated as sensitivity + specificity − 1. PPV and NPV are prevalence-dependent and should be interpreted in the context of the 8-year event rate in this cohort. These thresholds are study-specific exploratory cut-off values for risk stratification and should not be interpreted as clinically validated diagnostic criteria. Abbreviations: CMM, cardiometabolic multimorbidity; CHG, cholesterol–HDL-C–glucose index; TyG, triglyceride–glucose index; BMI, body mass index; WC, waist circumference; BRI, body roundness index; WHtR, waist-to-height ratio; CVAI, Chinese visceral adiposity index; RFM, relative fat mass; PPV, positive predictive value; NPV, negative predictive value.

Supplementary Table S5. Construction of the landmark cohort

Step	Number
Baseline analytic cohort in 2011	7,062
Free of CMM before or at the 2015 landmark and with assessable landmark status	6,029
With post-2015 follow-up information	5,630
Included in the landmark prediction model	5626
Post-2015 incident CMM events in the landmark model	411

Note. The 2015 landmark cohort was used only for post-landmark bootstrap optimism correction and calibration assessment and did not replace the 8-year prediction analysis based on the baseline cohort. The landmark cohort was used for post-landmark bootstrap optimism correction and calibration assessment following established principles for internal model validation [6].

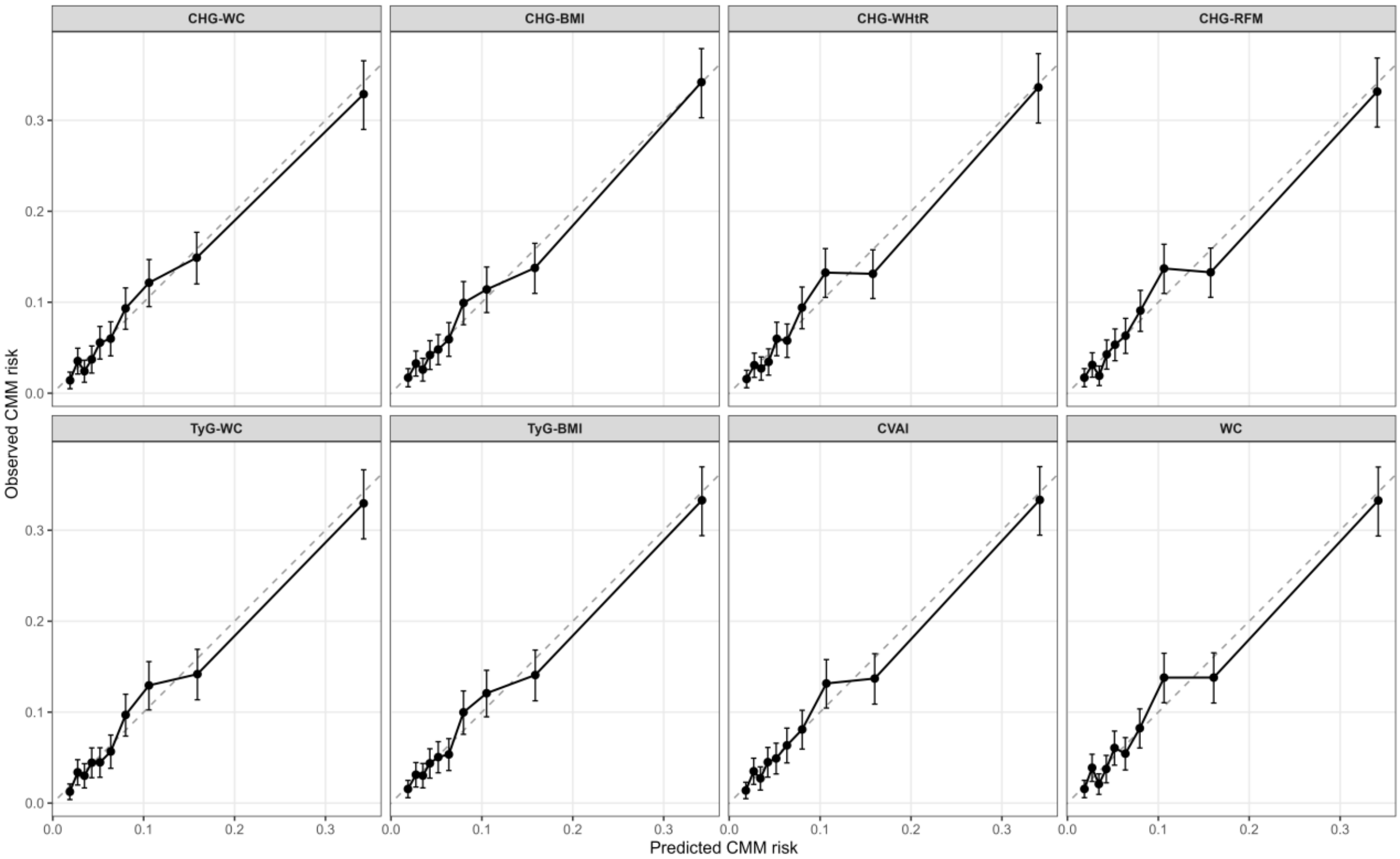

Supplementary Fig. S2. Decile-based calibration plots.

Note. Calibration plots compare the observed 8-year risk and predicted 8-year risk across deciles of predicted risk for representative index-extended models. The diagonal line indicates ideal calibration. This figure was used to evaluate model calibration and should not be interpreted as a formal superiority test among indices.

Supplementary Table S6. CKM stage progression associations

Layer	Marker	Family	Any progression, OR (95% CI)	Severe progression, OR (95% CI)	Progressed vs stable, OR (95% CI)	Improved vs stable, OR (95% CI)
More pronounced progression-related signal	CHG-RFM	CHG-related	1.556 (1.331, 1.818)	1.641 (1.288, 2.090)	1.373 (1.171, 1.610)	0.431 (0.359, 0.517)
More pronounced progression-related signal	RFM	Adiposity/body-shape	1.512 (1.282, 1.783)	1.493 (1.165, 1.914)	1.338 (1.131, 1.584)	0.445 (0.367, 0.540)
More pronounced progression-related signal	TyG-RFM	TyG-related	1.508 (1.290, 1.764)	1.669 (1.309, 2.129)	1.349 (1.149, 1.583)	0.471 (0.392, 0.565)
Consistent progression-related signal	CHG-BMI	CHG-related	1.363 (1.245, 1.491)	1.452 (1.259, 1.675)	1.271 (1.160, 1.393)	0.613 (0.547, 0.687)
Consistent progression-related signal	CHG-CVAI	CHG-related	1.371 (1.250, 1.504)	1.446 (1.240, 1.687)	1.286 (1.171, 1.413)	0.649 (0.581, 0.725)
Consistent progression-related signal	CHG-WC	CHG-related	1.362 (1.240, 1.496)	1.368 (1.177, 1.590)	1.259 (1.144, 1.385)	0.581 (0.518, 0.651)
Consistent progression-related signal	CVAI	Adiposity/body-shape	1.345 (1.232, 1.469)	1.388 (1.204, 1.598)	1.268 (1.160, 1.387)	0.668 (0.601, 0.743)
Consistent progression-related signal	TyG-BMI	TyG-related	1.339 (1.224, 1.463)	1.448 (1.258, 1.666)	1.261 (1.152, 1.381)	0.659 (0.590, 0.737)
Consistent progression-related signal	TyG-CVAI	TyG-related	1.352 (1.234, 1.482)	1.428 (1.228, 1.661)	1.275 (1.162, 1.400)	0.674 (0.604, 0.752)
Consistent progression-related signal	TyG-WC	TyG-related	1.326 (1.210, 1.454)	1.362 (1.177, 1.577)	1.242 (1.131, 1.363)	0.638 (0.571, 0.712)
Consistent progression-related signal	CHG-WHtR	CHG-related	1.323 (1.201, 1.457)	1.376 (1.181, 1.605)	1.223 (1.108, 1.349)	0.582 (0.517, 0.655)

Note. Values are adjusted ORs and 95% CIs. Any progression was defined as ΔCKM stage >0. Severe progression was defined as ΔCKM stage ≥+2, with ΔCKM stage ≤0 as the reference. Directional progression was evaluated using multinomial logistic regression, with stable CKM stage as the reference. The CKM progression models adjusted for age, sex, baseline CKM stage, education level, marital status, Hukou status, smoking status, drinking status, hypertension, diabetes, dyslipidemia, and kidney disease. Abbreviations: OR, odds ratio; CI, confidence interval; CKM, cardiovascular–kidney–metabolic. Supplementary Table S6 presents selected representative indices with consistent progression-related associations. Complete results for all 29 candidate indices are shown in Supplementary Fig. S1.

In the mediation analysis, the main model using completed 2015 CKM stage as the mediator showed that 7/10 CHG-related indices, 6/10 TyG-related indices, and 7/9 adiposity/body-shape indices showed positive natural indirect effects and positive proportions mediated, suggesting that CKM stage progression may represent a limited but consistent indirect pathway. Natural direct and indirect effects were estimated and interpreted within the counterfactual framework for mediation analysis [7].

In the main model using completed 2015 CKM stage as the mediator, nine indices did not meet the mediation-support criteria. For CHG, CHG-ABSI, TyG, TyG-ABSI, TyG-WWI, TyG-CI, CI, WWI, and ABSI, the NIE or PM did not show consistent evidence; therefore, these indices were not considered core indicators for the indirect CKM progression pathway.

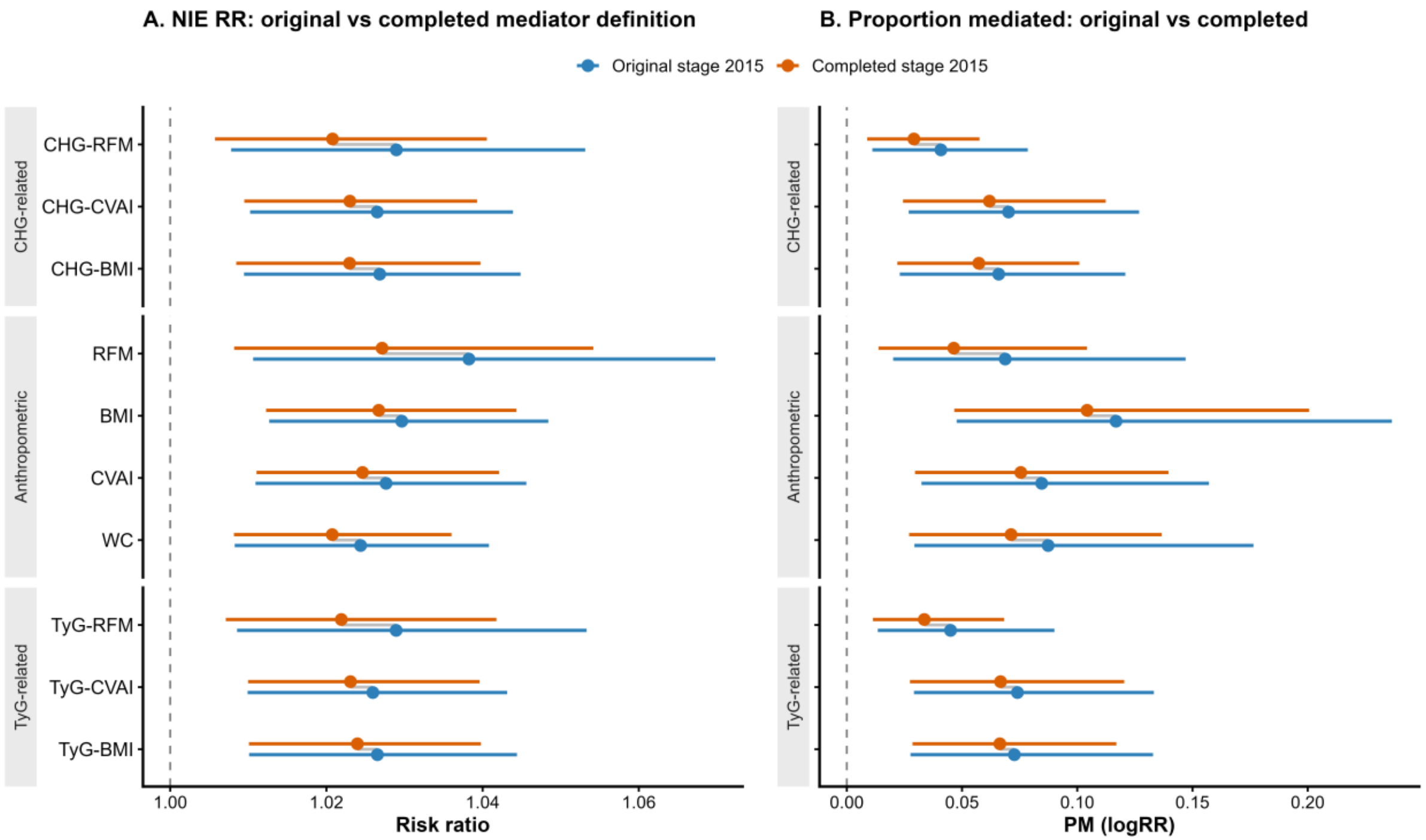

Supplementary Fig. S3. Mediation estimates by CKM definition.

Note. The figure compares mediation estimates obtained using the original and completed 2015 CKM stage definitions. If displayed, TE, NDE, NIE, and PM estimates should be interpreted as sensitivity comparisons across mediator definitions. Confidence intervals were estimated using 1,000 bootstrap resamples.

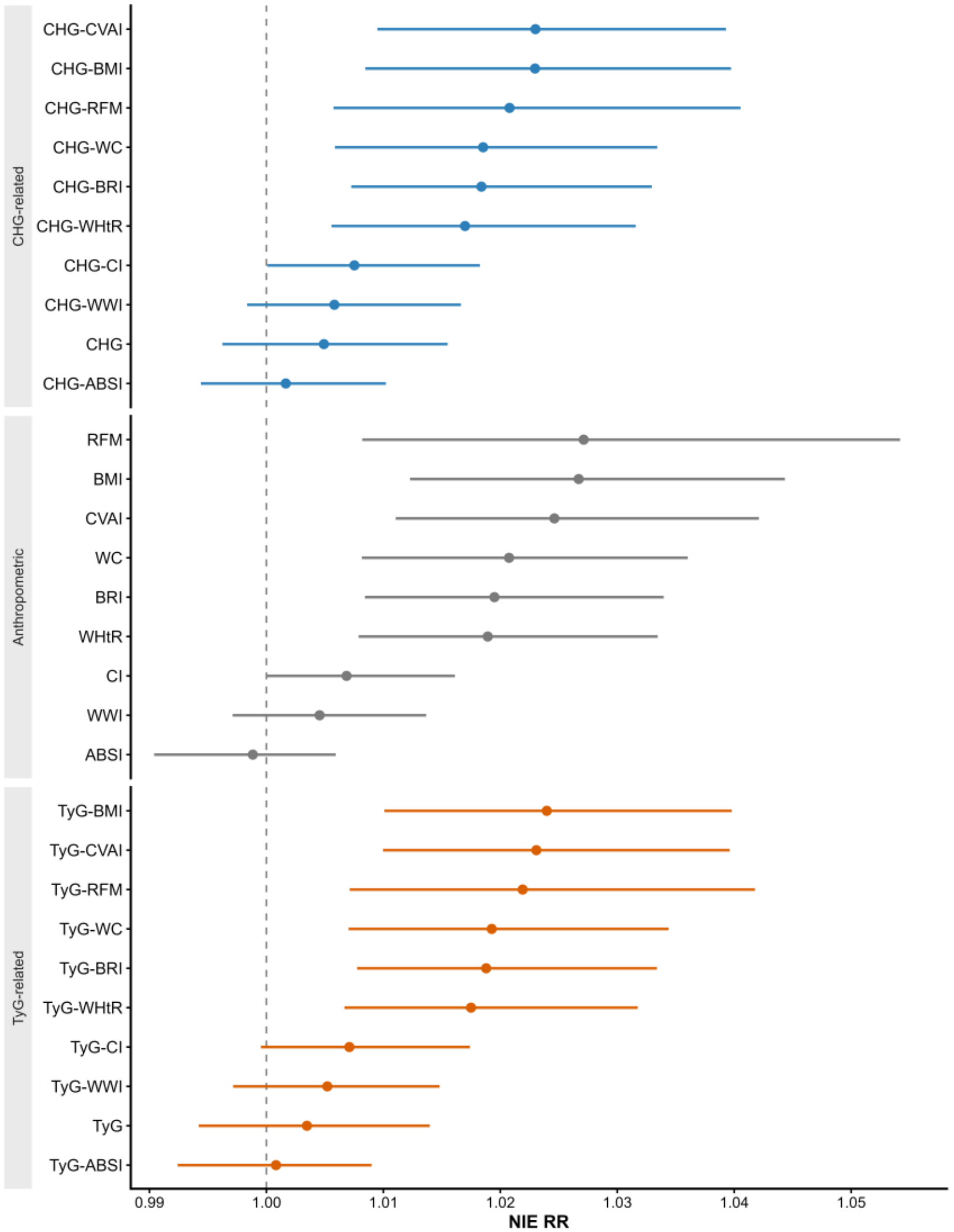

Supplementary Fig. S4. Natural indirect effects for all indices.

Note. Natural indirect effects are presented as RRs and 95% CIs. The completed 2015 CKM stage definition was used as the mediator. Abbreviations: NIE, natural indirect effect; RR, risk ratio; CI, confidence interval; CKM, cardiovascular–kidney–metabolic. Natural indirect effects were interpreted within the counterfactual natural-effect framework [7].

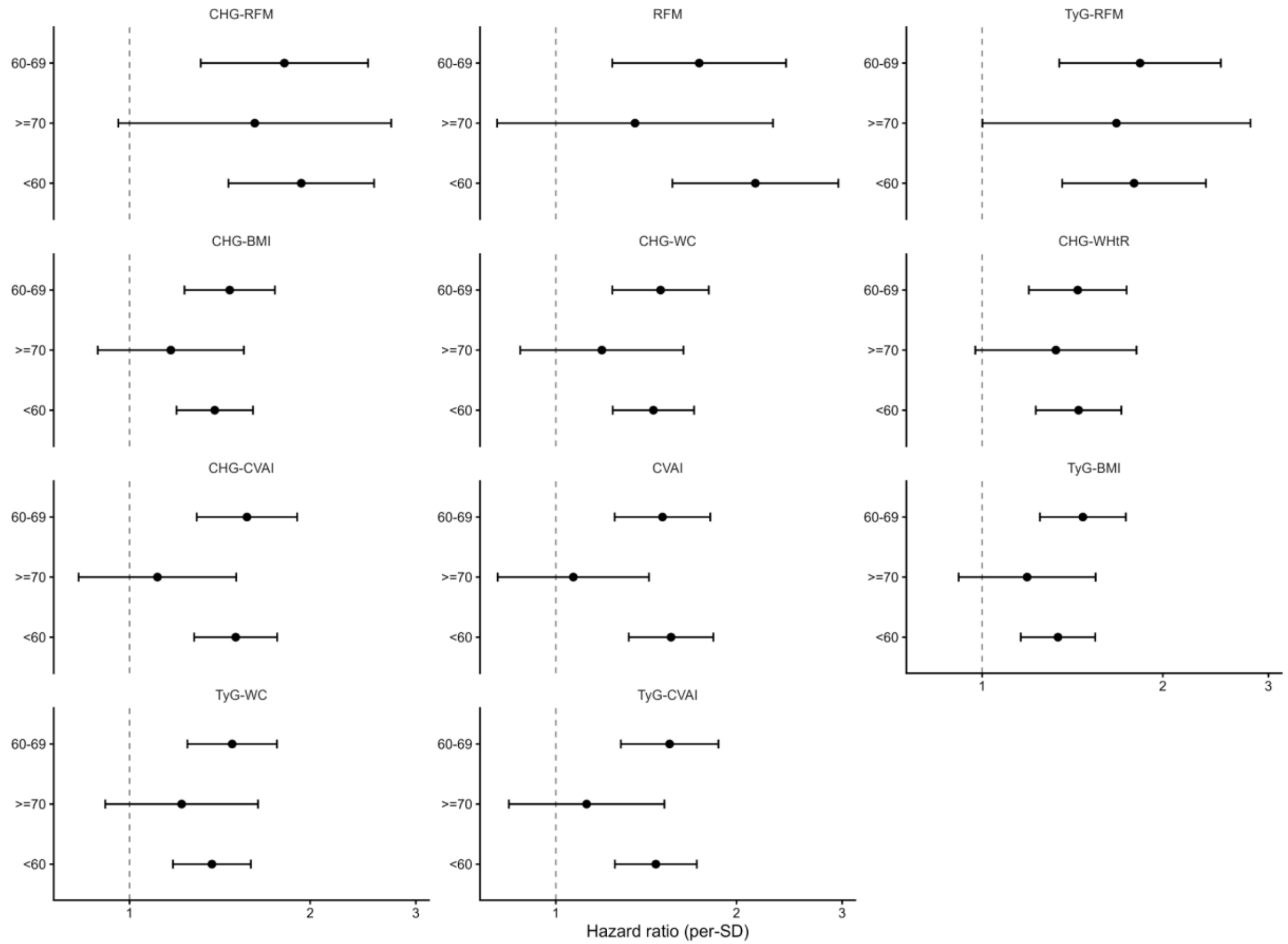

Supplementary Fig. S5. Age-stratified associations.

Note. Estimates are presented as HRs and 95% CIs and should be interpreted as descriptive stratified results rather than confirmatory evidence of effect modification. Abbreviations: HR, hazard ratio; CI, confidence interval; CMM, cardiometabolic multimorbidity.

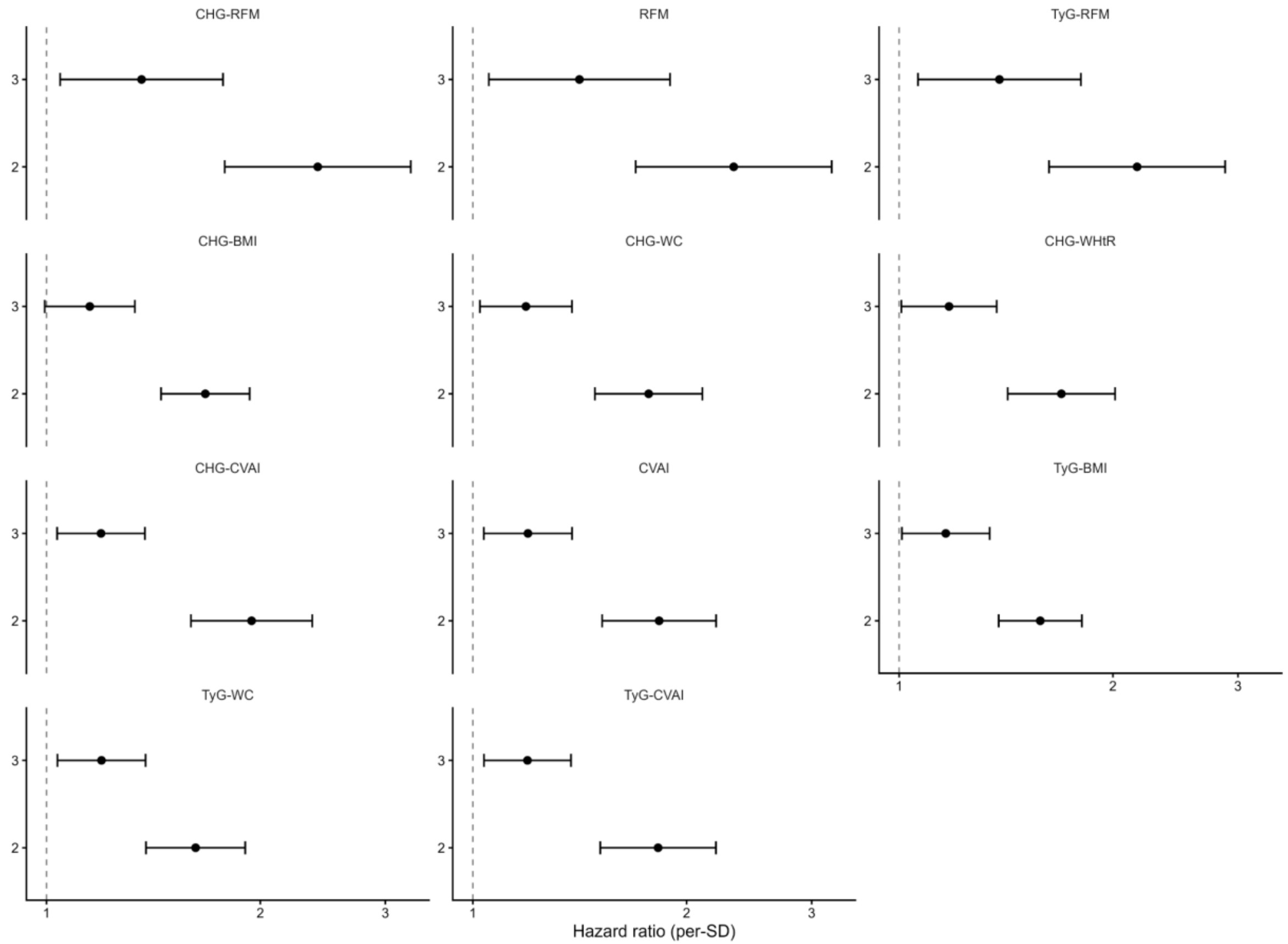

Supplementary Fig. S6. CKM stage-stratified associations.

Note. Estimates are presented as HRs and 95% CIs and should be interpreted as descriptive stratified results rather than confirmatory evidence of effect modification. Abbreviations: CKM, cardiovascular–kidney–metabolic; CMM, cardiometabolic multimorbidity; HR, hazard ratio; CI, confidence interval.

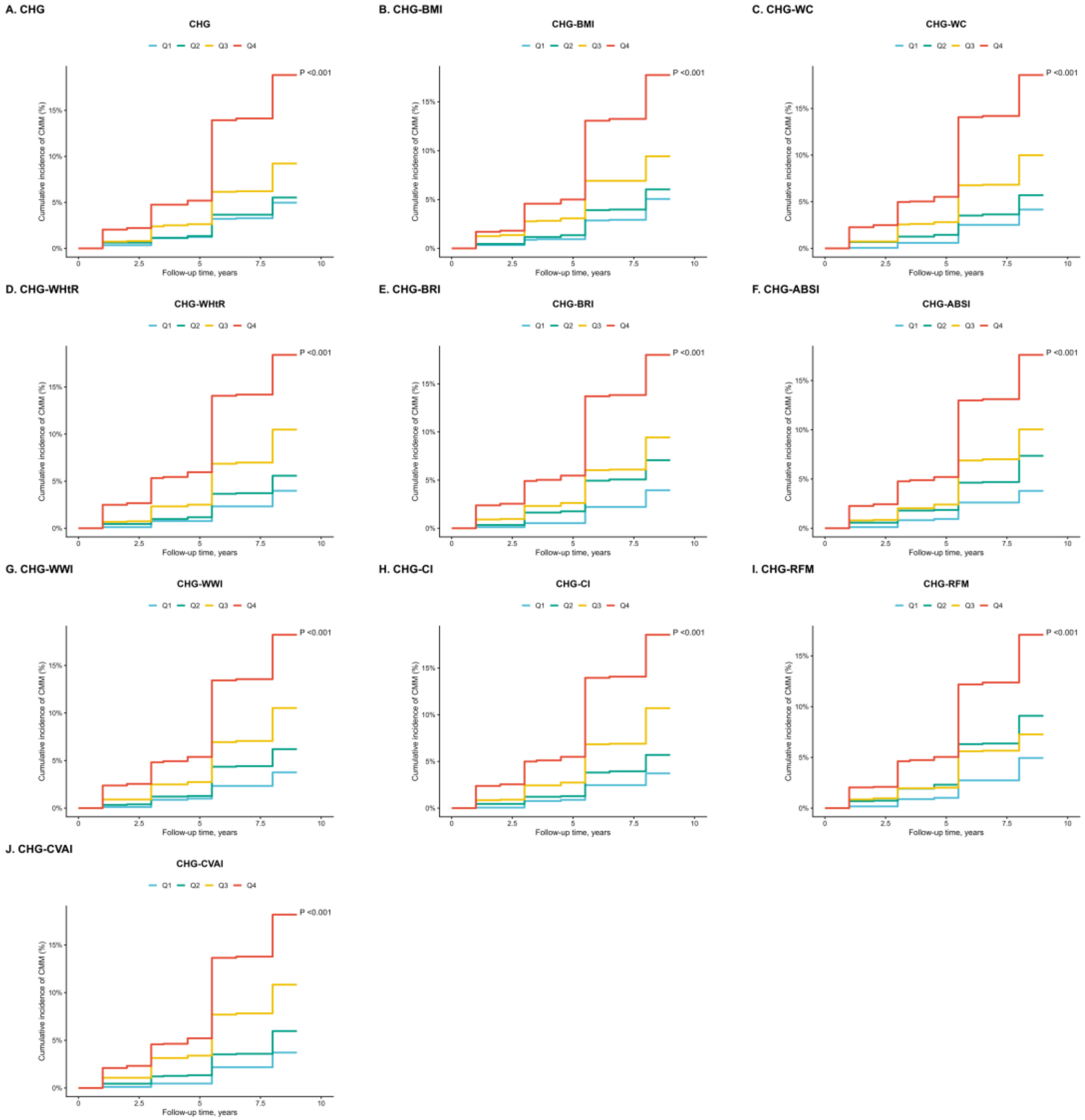

Supplementary Fig. S7A. Kaplan–Meier curves for CHG-related indices.

Participants were categorized into quartiles according to the baseline distribution of each index. Curves were used to provide a descriptive display of cumulative event differences during follow-up and were interpreted alongside adjusted Cox models.

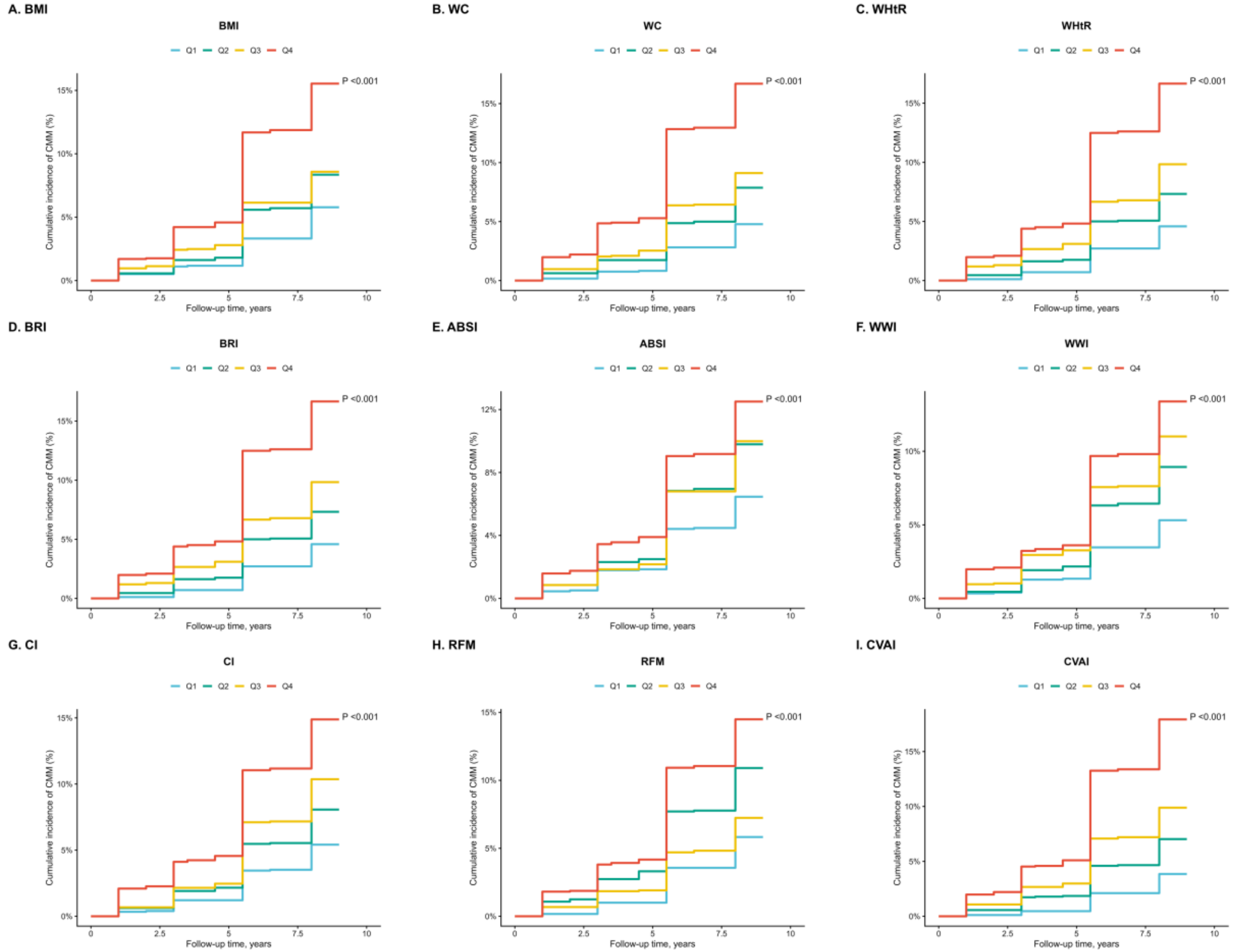

Supplementary Fig. S7B. Kaplan–Meier curves for adiposity/body-shape indices.

Participants were categorized into quartiles according to the baseline distribution of each index. Curves were used to provide a descriptive display of cumulative event differences during follow-up and were interpreted alongside adjusted Cox models.

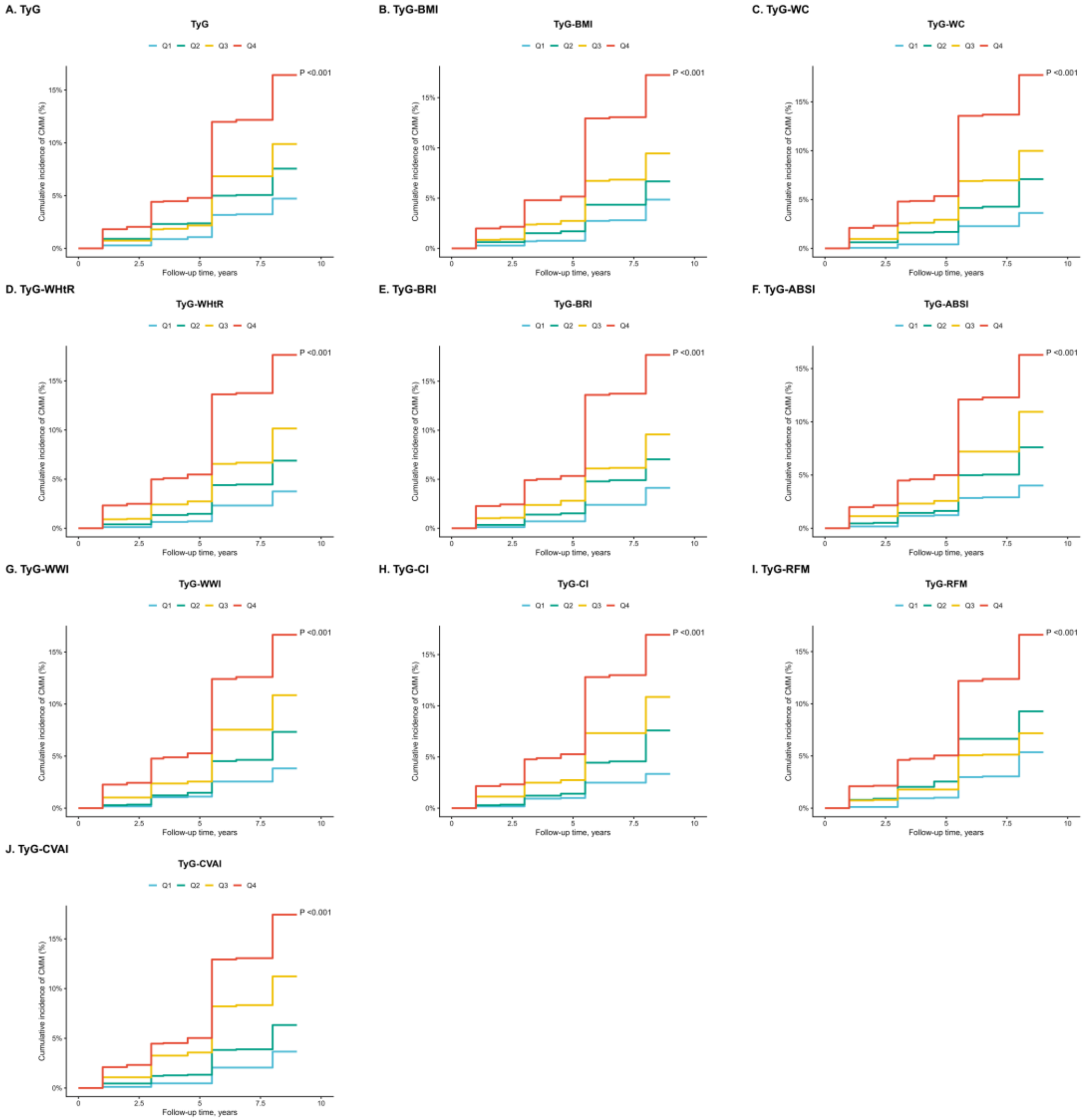

Supplementary Fig. S7C. Kaplan–Meier curves for TyG-related indices.

Participants were categorized into quartiles according to the baseline distribution of each index. Curves were used to provide a descriptive display of cumulative event differences during follow-up and were interpreted alongside adjusted Cox models.

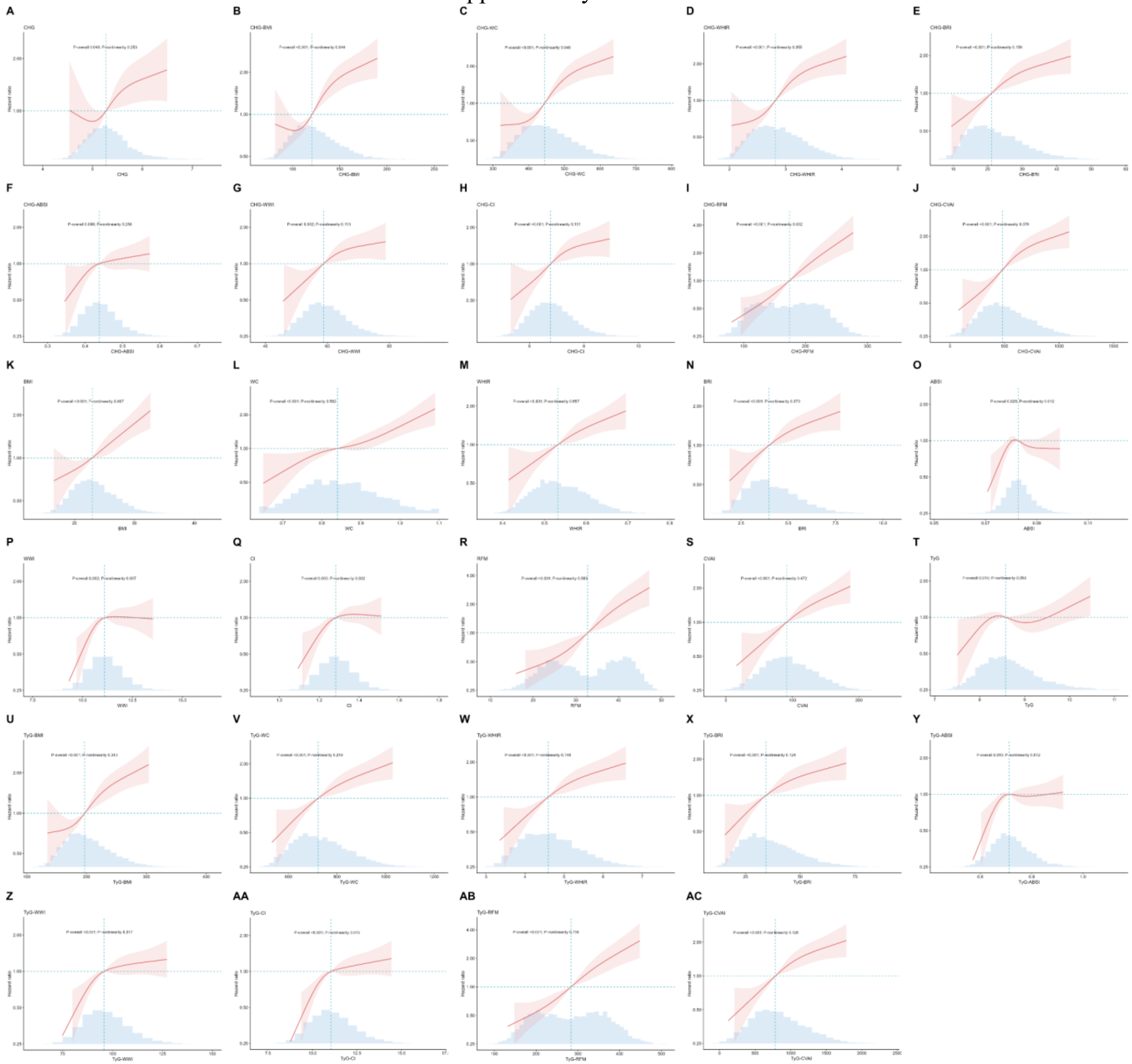

Supplementary Fig. S8. Dose–response curves for all indices.

Solid lines indicate HRs and shaded areas indicate 95% confidence intervals. Curves were fitted using the extended clinical-adjustment Model 4, with knots placed at the 5th, 35th, 65th, and 95th percentiles and the median value of each index used as the reference. The y-axis represents the adjusted HR for incident CMM. Panels A–AC correspond to CHG, CHG-BMI, CHG-WC, CHG-WHtR, CHG-BRI, CHG-ABSI, CHG-WWI, CHG-CI, CHG-RFM, CHG-CVAI, BMI, WC, WHtR, BRI, ABSI, WWI, CI, RFM, CVAI, TyG, TyG-BMI, TyG-WC, TyG-WHtR, TyG-BRI, TyG-ABSI, TyG-WWI, TyG-CI, TyG-RFM, and TyG-CVAI, respectively.

Abbreviations: CMM, cardiometabolic multimorbidity; CHG, cholesterol–HDL-C–glucose index; TyG, triglyceride–glucose index; BMI, body mass index; WC, waist circumference; WHtR, waist-to-height ratio; BRI, body roundness index; ABSI, a body shape index; WWI, weight-adjusted waist index; CI, conicity index; RFM, relative fat mass; CVAI, Chinese visceral adiposity index; HR, hazard ratio.

S7. Model 3 proportional-hazards diagnostics

Supplementary Table S7A. Model 3 Schoenfeld proportional-hazards diagnostics for five representative indices

Index	N	Events	Exposure-specific PH P	Global PH P	Exposure-specific violation
RFM	7014	569	0.069	0.002	No
CHG-RFM	7014	569	0.066	0.009	No
TyG-RFM	7014	569	0.046	0.007	Yes
BMI	7014	569	0.003	0.002	Yes
WC	7014	569	0.002	0.002	Yes

Note. Each representative index was entered separately in Model 3, adjusted for age, sex, education, marital status, Hukou, smoking, drinking, hypertension, diabetes, dyslipidemia, and kidney disease. Exposure-specific P values refer to the index term; global P values assess the model as a whole. An exposure-specific $P<0.05$ prompted a time-varying coefficient analysis in Supplementary Table S7B. Abbreviations: PH, proportional hazards; CMM, cardiometabolic multimorbidity; CHG, cholesterol–HDL-C–glucose index; TyG, triglyceride–glucose index; RFM, relative fat mass; BMI, body mass index; WC, waist circumference.

Supplementary Table S7B. Model 3 time-varying associations for indices with exposure-specific proportional-hazards departures

Index	N	Events	HR at year 1 (95% CI)	HR at year 4 (95% CI)	HR at year 8 (95% CI)	P for index × log(time)
TyG-RFM	7014	569	2.06 (1.60–2.65)	1.70 (1.43–2.01)	1.54 (1.28–1.85)	0.031
BMI	7014	569	1.56 (1.30–1.87)	1.32 (1.22–1.43)	1.21 (1.09–1.35)	0.038
WC	7014	569	1.70 (1.38–2.11)	1.36 (1.25–1.49)	1.22 (1.09–1.37)	0.014

Note. Time-varying Model 3 included the standardized index, index×log(time), and the Model 3 covariates. $HR(t)=exp[\beta index+\beta time\times log(t)]$. Results indicate that associations were strongest earlier and attenuated over time; the conventional Cox HR should therefore be interpreted as an average over follow-up rather than a constant effect. Abbreviations: HR, hazard ratio; CI, confidence interval; CHG, cholesterol–HDL-C–glucose index; TyG, triglyceride–glucose index; RFM, relative fat mass; BMI, body mass index; WC, waist circumference.

Supplementary Table S8. Fine–Gray competing-risk analysis

Variable	Events, n (%)	crude sHR (95% CI)	crude P value	adjusted sHR (95% CI)	adjusted P value
CHG (continuous variable per SD)	568 (8.1)	1.72 (1.61–1.83)	<0.001	1.16 (1.02–1.33)	0.029
CHG-BMI (continuous variable per SD)	568 (8.1)	1.69 (1.58–1.81)	<0.001	1.36 (1.24–1.50)	<0.001
CHG-WC (continuous variable per SD)	568 (8.1)	1.81 (1.69–1.95)	<0.001	1.40 (1.26–1.56)	<0.001
CHG-WHtR (continuous variable per SD)	568 (8.1)	1.85 (1.73–1.99)	<0.001	1.38 (1.24–1.54)	<0.001
CHG-BRI (continuous variable per SD)	568 (8.1)	1.70 (1.59–1.82)	<0.001	1.30 (1.19–1.43)	<0.001
CHG-ABSI (continuous variable per SD)	568 (8.1)	1.70 (1.59–1.82)	<0.001	1.11 (1.00–1.24)	0.050
CHG-WWI (continuous variable per SD)	568 (8.1)	1.79 (1.67–1.91)	<0.001	1.20 (1.08–1.35)	0.001
CHG-CI (continuous variable per SD)	568 (8.1)	1.80 (1.68–1.92)	<0.001	1.22 (1.10–1.37)	<0.001
CHG-RFM (continuous variable per SD)	568 (8.1)	1.64 (1.50–1.80)	<0.001	1.75 (1.45–2.10)	<0.001
CHG-CVAI (continuous variable per SD)	568 (8.1)	1.76 (1.65–1.89)	<0.001	1.40 (1.25–1.56)	<0.001
BMI (continuous variable per SD)	568 (8.1)	1.45 (1.35–1.56)	<0.001	1.29 (1.19–1.40)	<0.001
WC (continuous variable per SD)	568 (8.1)	1.59 (1.47–1.73)	<0.001	1.32 (1.20–1.45)	<0.001
WHtR (continuous variable per SD)	568 (8.1)	1.60 (1.49–1.73)	<0.001	1.31 (1.19–1.44)	<0.001
BRI (continuous variable per SD)	568 (8.1)	1.56 (1.46–1.68)	<0.001	1.28 (1.17–1.41)	<0.001
ABSI (continuous variable per SD)	568 (8.1)	1.24 (1.15–1.33)	<0.001	1.04 (0.96–1.14)	0.338
WWI (continuous variable per SD)	568 (8.1)	1.39 (1.30–1.49)	<0.001	1.13 (1.03–1.24)	0.010
CI (continuous variable per SD)	568 (8.1)	1.42 (1.33–1.53)	<0.001	1.15 (1.05–1.25)	0.002
RFM (continuous variable per SD)	568 (8.1)	1.39 (1.27–1.52)	<0.001	1.77 (1.44–2.17)	<0.001
CVAI (continuous variable per SD)	568 (8.1)	1.72 (1.59–1.85)	<0.001	1.39 (1.25–1.55)	<0.001
TyG (continuous variable per SD)	568 (8.1)	1.63 (1.52–1.74)	<0.001	1.13 (1.01–1.27)	0.032
TyG-BMI (continuous variable per SD)	568 (8.1)	1.63 (1.52–1.75)	<0.001	1.34 (1.23–1.47)	<0.001
TyG-WC (continuous variable per SD)	568 (8.1)	1.76 (1.64–1.88)	<0.001	1.36 (1.23–1.50)	<0.001
TyG-WHtR (continuous variable per SD)	568 (8.1)	1.78 (1.66–1.91)	<0.001	1.34 (1.22–1.49)	<0.001
TyG-BRI (continuous variable per SD)	568 (8.1)	1.66 (1.55–1.78)	<0.001	1.30 (1.18–1.42)	<0.001
TyG-ABSI (continuous variable per SD)	568 (8.1)	1.61 (1.51–1.72)	<0.001	1.10 (1.00–1.21)	0.044
TyG-WWI (continuous variable per SD)	568 (8.1)	1.70 (1.59–1.82)	<0.001	1.18 (1.07–1.30)	0.001
TyG-CI (continuous variable per SD)	568 (8.1)	1.72 (1.60–1.84)	<0.001	1.19 (1.08–1.32)	<0.001
TyG-RFM (continuous variable per SD)	568 (8.1)	1.58 (1.45–1.73)	<0.001	1.70 (1.43–2.03)	<0.001
TyG-CVAI (continuous variable per SD)	568 (8.1)	1.74 (1.62–1.86)	<0.001	1.38 (1.24–1.53)	<0.001

Abbreviations: sHR, subdistribution hazard ratio; CMM, cardiometabolic multimorbidity; CKM syndrome, cardiovascular kidney-metabolic syndrome; CHG, cholesterol, high-density lipoprotein cholesterol, and glucose index; TyG, triglyceride-glucose index; BMI, body mass index; WC, waist circumference; WHtR, waist height ratio index; BRI, body roundness index; ABSI, a body shape index; WWI, weight-adjusted waist index; CI, conicity index; RFM, relative fat mass; CVAI, Chinese visceral adiposity index; SBP, systolic blood pressure; DBP, diastolic blood pressure; HDL-C, high-density lipoprotein cholesterol; LDL-C, low-density lipoprotein cholesterol; eGFR, estimated glomerular filtration rate; UA, uric acid; BUN, blood urea nitrogen; Hb, hemoglobin; MCV, mean corpuscular volume; PCV, packed cell volume; WBC, white blood cell count.

Note. Death was treated as a competing event, and subdistribution hazard ratios were estimated using Fine–Gray competing-risk regression [5]. The competing-risk model adjusted for age, sex, education level, marital status, Hukou, smoking status, drinking status, hypertension, diabetes, dyslipidemia, kidney disease, baseline CKM stage, SBP, DBP, HDL-C, LDL-C, eGFR, UA, BUN, Hb, MCV, PCV, platelet count, and WBC.

S9–S13. Multiplicity, inter-index overlap, and sensitivity analyses

Supplementary Table S9. Benjamini–Hochberg FDR-adjusted per-SD Model 3 associations across 29 candidate indices

Family	Index	HR per SD	95% CI	Nominal P	BH q-value	q<0.05
CHG-related	CHG	1.20	1.08–1.32	<0.001	<0.001	Yes
CHG-related	CHG-BMI	1.35	1.24–1.47	<0.001	<0.001	Yes
CHG-related	CHG-WC	1.37	1.25–1.51	<0.001	<0.001	Yes
CHG-related	CHG-WHtR	1.37	1.25–1.51	<0.001	<0.001	Yes
CHG-related	CHG-BRI	1.33	1.22–1.45	<0.001	<0.001	Yes
CHG-related	CHG-ABSI	1.16	1.06–1.27	0.002	0.002	Yes
CHG-related	CHG-WWI	1.24	1.13–1.37	<0.001	<0.001	Yes
CHG-related	CHG-CI	1.25	1.14–1.38	<0.001	<0.001	Yes
CHG-related	CHG-RFM	1.72	1.47–2.03	<0.001	<0.001	Yes
CHG-related	CHG-CVAI	1.35	1.23–1.48	<0.001	<0.001	Yes
Adiposity/body-shape	RFM	1.85	1.52–2.25	<0.001	<0.001	Yes
Adiposity/body-shape	CVAI	1.36	1.24–1.50	<0.001	<0.001	Yes
Adiposity/body-shape	WHtR	1.34	1.22–1.46	<0.001	<0.001	Yes
Adiposity/body-shape	BRI	1.31	1.20–1.43	<0.001	<0.001	Yes
Adiposity/body-shape	ABSI	1.05	0.96–1.15	0.245	0.245	No
Adiposity/body-shape	WWI	1.16	1.05–1.28	0.003	0.003	Yes
Adiposity/body-shape	CI	1.17	1.07–1.28	<0.001	<0.001	Yes
Adiposity/body-shape	BMI	1.30	1.20–1.41	<0.001	<0.001	Yes
Adiposity/body-shape	WC	1.34	1.23–1.46	<0.001	<0.001	Yes
TyG-related	TyG	1.11	1.01–1.23	0.033	0.036	Yes
TyG-related	TyG-BMI	1.32	1.21–1.44	<0.001	<0.001	Yes
TyG-related	TyG-WC	1.34	1.22–1.46	<0.001	<0.001	Yes
TyG-related	TyG-WHtR	1.33	1.21–1.47	<0.001	<0.001	Yes
TyG-related	TyG-BRI	1.31	1.20–1.43	<0.001	<0.001	Yes
TyG-related	TyG-ABSI	1.10	1.01–1.21	0.036	0.037	Yes
TyG-related	TyG-WWI	1.18	1.07–1.31	<0.001	<0.001	Yes
TyG-related	TyG-CI	1.19	1.08–1.31	<0.001	<0.001	Yes
TyG-related	TyG-RFM	1.67	1.41–1.98	<0.001	<0.001	Yes
TyG-related	TyG-CVAI	1.33	1.21–1.46	<0.001	<0.001	Yes

Note. Each index was standardized and entered separately in Model 3. Benjamini–Hochberg correction was applied across the 29 primary per-SD P values. Model 3 adjusted for age, sex, education, marital status, Hukou, smoking, drinking, hypertension, diabetes, dyslipidemia, and kidney disease. Abbreviations: FDR, false discovery rate; HR, hazard ratio; CI, confidence interval; BH, Benjamini–Hochberg; CHG, cholesterol–HDL-C–glucose index; TyG, triglyceride–glucose index.

Supplementary Table S10. Spearman correlation matrix among all 29 candidate indices (N=7,062)

Index	CHG	CHG-BMI	CHG-WC	CHG-WHtR	CHG-BRI	CHG-ABSI	CHG-WWI	CHG-CI	CHG-RFM	CHG-CVAI	RFM	CVAI	WHtR	BRI	ABSI	WWI	CI	BMI	WC	TyG	TyG-BMI	TyG-WC	TyG-WHtR	TyG-BRI	TyG-ABSI	TyG-WWI	TyG-CI	TyG-RFM	TyG-CVAI
CHG	1	0.661	0.729	0.708	0.523	0.772	0.755	0.795	0.426	0.647	0.214	0.545	0.354	0.354	0.137	0.217	0.256	0.351	0.377	0.747	0.572	0.624	0.601	0.474	0.594	0.597	0.636	0.365	0.611
CHG-BMI	0.661	1	0.882	0.863	0.822	0.457	0.6	0.628	0.599	0.84	0.501	0.813	0.752	0.752	0.012	0.299	0.345	0.925	0.787	0.531	0.964	0.84	0.815	0.799	0.36	0.517	0.547	0.566	0.823
CHG-WC	0.729	0.882	1	0.943	0.894	0.758	0.815	0.879	0.596	0.929	0.485	0.898	0.813	0.813	0.395	0.54	0.659	0.746	0.898	0.579	0.836	0.95	0.887	0.867	0.651	0.72	0.789	0.558	0.909
CHG-WHtR	0.708	0.863	0.943	1	0.969	0.77	0.899	0.887	0.768	0.906	0.671	0.876	0.9	0.9	0.435	0.686	0.693	0.731	0.834	0.577	0.823	0.9	0.952	0.946	0.676	0.817	0.809	0.733	0.888
CHG-BRI	0.523	0.822	0.894	0.969	1	0.669	0.83	0.802	0.786	0.879	0.744	0.879	0.979	0.979	0.483	0.763	0.758	0.774	0.889	0.446	0.805	0.877	0.949	0.99	0.616	0.785	0.762	0.765	0.87
CHG-ABSI	0.772	0.457	0.758	0.77	0.669	1	0.947	0.974	0.514	0.691	0.371	0.628	0.558	0.558	0.696	0.679	0.742	0.201	0.546	0.597	0.393	0.69	0.698	0.636	0.875	0.837	0.867	0.47	0.666
CHG-WWI	0.755	0.6	0.815	0.899	0.83	0.947	1	0.972	0.705	0.772	0.582	0.719	0.736	0.736	0.639	0.776	0.762	0.384	0.63	0.602	0.546	0.756	0.839	0.801	0.838	0.903	0.879	0.664	0.749
CHG-CI	0.795	0.628	0.879	0.887	0.802	0.974	0.972	1	0.604	0.811	0.467	0.756	0.697	0.697	0.635	0.697	0.764	0.401	0.692	0.622	0.568	0.814	0.818	0.77	0.85	0.863	0.894	0.56	0.786
CHG-RFM	0.426	0.599	0.596	0.768	0.786	0.514	0.705	0.604	1	0.607	0.968	0.591	0.766	0.766	0.344	0.672	0.533	0.539	0.537	0.401	0.594	0.591	0.765	0.784	0.495	0.692	0.592	0.987	0.603
CHG-CVAI	0.647	0.84	0.929	0.906	0.879	0.691	0.772	0.811	0.607	1	0.516	0.99	0.817	0.817	0.389	0.558	0.645	0.736	0.86	0.594	0.831	0.924	0.894	0.872	0.651	0.737	0.784	0.593	0.994
RFM	0.214	0.501	0.485	0.671	0.744	0.371	0.582	0.467	0.968	0.516	1	0.53	0.771	0.771	0.355	0.692	0.541	0.523	0.526	0.241	0.518	0.505	0.693	0.754	0.391	0.604	0.491	0.97	0.521
CVAI	0.545	0.813	0.898	0.876	0.879	0.628	0.719	0.756	0.591	0.99	0.53	1	0.842	0.842	0.406	0.578	0.669	0.755	0.887	0.525	0.818	0.91	0.88	0.88	0.612	0.707	0.752	0.586	0.991
WHtR	0.354	0.752	0.813	0.9	0.979	0.558	0.736	0.697	0.766	0.817	0.771	0.842	1	1	0.501	0.793	0.777	0.774	0.893	0.32	0.753	0.819	0.903	0.98	0.538	0.723	0.689	0.757	0.815
BRI	0.354	0.752	0.813	0.9	0.979	0.558	0.736	0.697	0.766	0.817	0.771	0.842	1	1	0.501	0.793	0.777	0.774	0.893	0.32	0.753	0.819	0.903	0.98	0.538	0.723	0.689	0.757	0.815
ABSI	0.137	0.012	0.395	0.435	0.483	0.696	0.639	0.635	0.344	0.389	0.355	0.406	0.501	0.501	1	0.858	0.917	-0.054	0.454	0.128	0.007	0.402	0.44	0.485	0.707	0.647	0.649	0.342	0.389
WWI	0.217	0.299	0.54	0.686	0.763	0.679	0.776	0.697	0.672	0.558	0.692	0.578	0.793	0.793	0.858	1	0.928	0.267	0.601	0.211	0.3	0.551	0.697	0.769	0.687	0.786	0.711	0.669	0.559
CI	0.256	0.345	0.659	0.693	0.758	0.742	0.762	0.764	0.533	0.645	0.541	0.669	0.777	0.777	0.917	0.928	1	0.307	0.742	0.23	0.34	0.665	0.695	0.759	0.737	0.757	0.767	0.527	0.644
BMI	0.351	0.925	0.746	0.731	0.774	0.201	0.384	0.401	0.539	0.736	0.523	0.755	0.774	0.774	-0.054	0.267	0.307	1	0.808	0.301	0.926	0.743	0.722	0.77	0.16	0.352	0.373	0.527	0.732
WC	0.377	0.787	0.898	0.834	0.889	0.546	0.63	0.692	0.537	0.86	0.526	0.887	0.893	0.893	0.454	0.601	0.742	0.808	1	0.322	0.781	0.898	0.825	0.885	0.509	0.6	0.669	0.525	0.855
TyG	0.747	0.531	0.579	0.577	0.446	0.597	0.602	0.622	0.401	0.594	0.241	0.525	0.32	0.32	0.128	0.211	0.23	0.301	0.322	1	0.618	0.687	0.678	0.488	0.757	0.741	0.775	0.447	0.626
TyG-BMI	0.572	0.964	0.836	0.823	0.805	0.393	0.546	0.568	0.594	0.831	0.518	0.818	0.753	0.753	0.007	0.3	0.34	0.926	0.781	0.618	1	0.875	0.854	0.818	0.416	0.57	0.599	0.601	0.84
TyG-WC	0.624	0.84	0.95	0.9	0.877	0.69	0.756	0.814	0.591	0.924	0.505	0.91	0.819	0.819	0.402	0.551	0.665	0.743	0.898	0.687	0.875	1	0.939	0.893	0.731	0.795	0.864	0.602	0.935
TyG-WHtR	0.601	0.815	0.887	0.952	0.949	0.698	0.839	0.818	0.765	0.894	0.693	0.88	0.903	0.903	0.44	0.697	0.695	0.722	0.825	0.678	0.854	0.939	1	0.969	0.751	0.889	0.878	0.778	0.907
TyG-BRI	0.474	0.799	0.867	0.946	0.99	0.636	0.801	0.77	0.784	0.872	0.754	0.88	0.98	0.98	0.485	0.769	0.759	0.77	0.885	0.488	0.818	0.893	0.969	1	0.647	0.815	0.791	0.785	0.877
TyG-ABSI	0.594	0.36	0.651	0.676	0.616	0.875	0.838	0.85	0.495	0.651	0.391	0.612	0.538	0.538	0.707	0.687	0.737	0.16	0.509	0.757	0.416	0.731	0.751	0.647	1	0.944	0.971	0.526	0.674
TyG-WWI	0.597	0.517	0.72	0.817	0.785	0.837	0.903	0.863	0.692	0.737	0.604	0.707	0.723	0.723	0.647	0.786	0.757	0.352	0.6	0.741	0.57	0.795	0.889	0.815	0.944	1	0.97	0.718	0.758
TyG-CI	0.636	0.547	0.789	0.809	0.762	0.867	0.879	0.894	0.592	0.784	0.491	0.752	0.689	0.689	0.649	0.711	0.767	0.373	0.669	0.775	0.599	0.864	0.878	0.791	0.971	0.97	1	0.618	0.805
TyG-RFM	0.365	0.566	0.558	0.733	0.765	0.47	0.664	0.56	0.987	0.593	0.97	0.586	0.757	0.757	0.342	0.669	0.527	0.527	0.525	0.447	0.601	0.602	0.778	0.785	0.526	0.718	0.618	1	0.605
TyG-CVAI	0.611	0.823	0.909	0.888	0.87	0.666	0.749	0.786	0.603	0.994	0.521	0.991	0.815	0.815	0.389	0.559	0.644	0.732	0.855	0.626	0.84	0.935	0.907	0.877	0.674	0.758	0.805	0.605	1

Note. Values are Spearman correlation coefficients calculated in the fixed baseline cohort. The matrix demonstrates structural overlap among indices and should not be interpreted as evidence that the indices provide independent information. Abbreviations: CHG, cholesterol–HDL-C–glucose index; TyG, triglyceride–glucose index; BMI, body mass index; WC, waist circumference; WHtR, waist-to-height ratio; BRI, body roundness index; ABSI, a body shape index; WWI, weight-adjusted waist index; CI, conicity index; RFM, relative fat mass; CVAI, Chinese visceral adiposity index.

Supplementary Table S11. Paired CHG–TyG dual-entry Model 3 analyses

Paired model	Index	N	Events	HR per SD	95% CI	P value
CHG + TyG	CHG	7014	569	1.22	1.06–1.40	0.005
CHG + TyG	TyG	7014	569	0.97	0.85–1.11	0.703
CHG-RFM + TyG-RFM	CHG-RFM	7014	569	1.87	1.19–2.93	0.007
CHG-RFM + TyG-RFM	TyG-RFM	7014	569	0.91	0.57–1.46	0.706

Note. Both indices in each pair were standardized and entered simultaneously with Model 3 covariates. Because the paired indices share glucose and related metabolic or adiposity components and were highly correlated, the results assess information overlap and mutual attenuation; they are not tests of definitive causal independence or superiority. Abbreviations: HR, hazard ratio; CI, confidence interval; CHG, cholesterol–HDL-C–glucose index; TyG, triglyceride–glucose index; RFM, relative fat mass.

Supplementary Table S12. Sensitivity analysis excluding participants with diabetes at baseline

Index	N	Events	HR per SD	95% CI	P value
RFM	5930	318	1.965	1.520–2.541	<0.001
CHG-RFM	5930	318	2.014	1.592–2.549	<0.001
TyG-RFM	5930	318	1.821	1.437–2.308	<0.001
BMI	5930	318	1.334	1.200–1.484	<0.001
WC	5930	318	1.364	1.216–1.531	<0.001

Note. Participants with baseline diabetes were excluded. Each index was standardized and entered separately in a Model 3 structure adjusted for age, sex, education, marital status, Hukou, smoking, drinking, hypertension, dyslipidemia, and kidney disease; diabetes was omitted because all participants in this analysis were diabetes-free at baseline. Abbreviations: HR, hazard ratio; CI, confidence interval; CHG, cholesterol–HDL-C–glucose index; TyG, triglyceride–glucose index; RFM, relative fat mass; BMI, body mass index; WC, waist circumference.

Supplementary Table S13. Exploratory CKD-inclusive CMM sensitivity analysis for five representative indices

Index	N	Events	HR per SD (95% CI)	P value
RFM	6896	837	1.69 (1.44–1.98)	<0.001
CHG-RFM	6896	837	1.58 (1.38–1.81)	<0.001
TyG-RFM	6896	837	1.55 (1.35–1.79)	<0.001
BMI	6896	837	1.28 (1.20–1.37)	<0.001
WC	6896	837	1.30 (1.21–1.40)	<0.001

Note. The expanded outcome was defined as the coexistence of at least two of diabetes, heart disease, stroke, and CKD. Participants who already met this expanded definition at baseline were excluded from this sensitivity analysis. Each index was standardized and entered separately in Model 3. This analysis was exploratory and did not replace the primary CMM definition. Abbreviations: CKD, chronic kidney disease; CMM, cardiometabolic multimorbidity; HR, hazard ratio; CI, confidence interval; CHG, cholesterol–HDL-C–glucose index; TyG, triglyceride–glucose index; RFM, relative fat mass; BMI, body mass index; WC, waist circumference.

Abbreviations

ABSI, a body shape index; AUC, area under the curve; BMI, body mass index; BRI, body roundness index.

CHG, cholesterol, high-density lipoprotein cholesterol, and glucose index; CHARLS, China Health and Retirement Longitudinal Study.

CKM, cardiovascular–kidney–metabolic; CMM, cardiometabolic multimorbidity; cNRI, continuous net reclassification improvement; CVAI, Chinese visceral adiposity index; GVIF, generalized variance inflation factor.

IDI, integrated discrimination improvement; NRI, net reclassification improvement; RFM, relative fat mass; TyG, triglyceride–glucose index; WHtR, waist-to-height ratio; WWI, weight-adjusted waist index.

Supplementary References

1. Ndumele CE, Rangaswami J, Chow SL, Neeland IJ, Tuttle KR, Khan SS, Coresh J, Mathew RO, Baker-Smith CM, Carnethon MR, et al. Cardiovascular-Kidney-Metabolic Health: A Presidential Advisory From the American Heart Association. Circulation. 2023;148(20):1606–1635.

2. Jin T, Tang X, Han Y, Fan H, Qin Q, Jiang H, et al. Relationship between nine triglyceride-glucose-related indices and cardiometabolic multimorbidity incidence in patients with cardiovascular-kidney-metabolic syndrome stage 0–3: a nationwide prospective cohort study. Cardiovasc Diabetol. 2026;25:36. doi:10.1186/s12933-026-03077-4.

3. Zhao Y, Hu Y, Smith JP, Strauss J, Yang G. Cohort profile: The China Health and Retirement Longitudinal Study (CHARLS). Int J Epidemiol. 2014;43(1):61–68. doi:10.1093/ije/dys203.

4. Pencina MJ, D'Agostino RB Sr, D'Agostino RB Jr, Vasan RS. Evaluating the added predictive ability of a new marker: from area under the ROC curve to reclassification and beyond. Stat Med. 2008;27(2):157–172. doi:10.1002/sim.2929.

5. Fine JP, Gray RJ. A proportional hazards model for the subdistribution of a competing risk. J Am Stat Assoc. 1999;94(446):496–509.

6. Harrell FE Jr. Regression Modeling Strategies. 2nd ed. Cham: Springer; 2015.

7. VanderWeele TJ. Explanation in Causal Inference: Methods for Mediation and Interaction. New York: Oxford University Press; 2015.

8. Blanche P, Dartigues JF, Jacqmin-Gadda H. Estimating and comparing time-dependent areas under receiver operating characteristic curves for censored event times with competing risks. Stat Med. 2013;32(30):5381–5397. doi:10.1002/sim.5958.
